# Supplementary material for: A computational framework to study sub-cellular RNA localization
Source: Nat Commun. 2018 Nov 2;9:4584. doi: 10.1038/s41467-018-06868-w (PMC6214940; doi:10.1038/s41467-018-06868-w)
Supplement: Supplementary file 1 — Supplementary Information [file 41467_2018_6868_MOESM1_ESM.pdf]

Supplementary Information for

A computational framework to study sub-cellular mRNA localization

Supplementary Note 1: Simulation of realistic smFISH images

1.1. OVERVIEW

The overall goal of this project is to provide the methodological framework to quantitatively analyze the spatial distribution of single RNA molecules inside cells from large-scale smFISH image data sets. For this, feature sets capturing the spatial properties of point clouds are designed and used in an unsupervised learning setting in order to identify RNA localization patterns. One major problem with such an unsupervised approach is that in the absence of ground truth it can neither be validated, nor optimized, nor are we able to study its limitations. But all of this is necessary if we want to draw biological conclusions from this type of data sets.

While benchmark datasets exist already for cell segmentation or protein localization[1,2] and even though more and more large-scale smFISH studies are performed[3–5], there is no annotated ground truth dataset for mRNA localization. Here, we present a simulation framework of smFISH images with non-random 3D mRNA localization. We base these simulations on experimental data, providing accurate 3D contours for cells and nuclei. Further, mRNAs are simulated considering realistic variations in their intensity and different experimentally observed localization patterns. Altogether, our approach yields (1) a set of realistic smFISH images providing a benchmark dataset for feature design and machine learning approaches for the classification of mRNA localization patterns and (2) a simulation environment, with which new patterns can be generated according to physical rules of RNA localization.

1.2. DETERMINING 3D SHAPE OF CELLS AND NUCLEI

The first step in the simulation of 3D smFISH images is to obtain realistic cellular and nuclear shapes. To obtain such shapes, generative model approaches have been developed[6,7]. However, for this study, we chose a different approach where these shapes are directly extracted from experimental data, yielding an accurate 3D description of the cells used in our study.

To obtain the 3D cellular volume, we used an approach described in Padovan-Merhar et al.[8]. Here, smFISH against the highly-expressed house-keeping gene *GAPDH* was used to infer the cellular volume. We designed an experiment with 4 different channels in HeLa Kyoto cells as follows (see also Online Methods for more details).

(1) smFISH against *GAPDH* (Cy5) in order to infer the 3D volume of each cell.

(2) Cytoplasmic marker (HCS CellMask™ Deep Red, Molecular Probes) in order to accurately segment the cellular boundaries in 2D.

(3) DAPI for a nuclear stain in order to accurately segment the cell nucleus in 2D.

(4) mock FISH against a GFP sequence (Cy3), which is not expressed in these cells, thus providing a realistic background signal[9].

We used the DAPI and the CellMask™ to perform 2D segmentation of nuclei and cells (Fig S1a) with the open-source software CellCognition[10] using a standard segmentation workflow: Otsu thresholding and a watershed based split for nuclei in the DAPI channel. Each nucleus then serves as a seed for a watershed segmentation to obtain the cells in the CellMask™ channel. We inferred the 3D volume of each cell using the *GAPDH* smFISH experiment (Fig S1b). We localized individual *GAPDH* mRNA molecules with FISH-quant in 3D[11] for each segmented cell. To guarantee that the cell border is correctly considered at the coverslip, we defined the z-position of the coverslip as the minimum z-position of all detected mRNAs. We then randomly sampled 2000 data-points from the 2D CellMask™ segmentation and assigned this

minimum z-value as the corresponding z-position. We then fused these two point clouds (actual *GAPDH* positons and point-cloud defining the coverslip). We then determined the 3D cellular volume (Fig S1c) as the 3D boundary containing this point cloud (MATLAB function *boundary*). One assumption is that *GAPDH* is present in the entire cytoplasmic space. While it is possible that the actual cellular volume is slightly larger, we assume that this approach nevertheless provides a suitable reference volume for the accessible space for mRNAs.

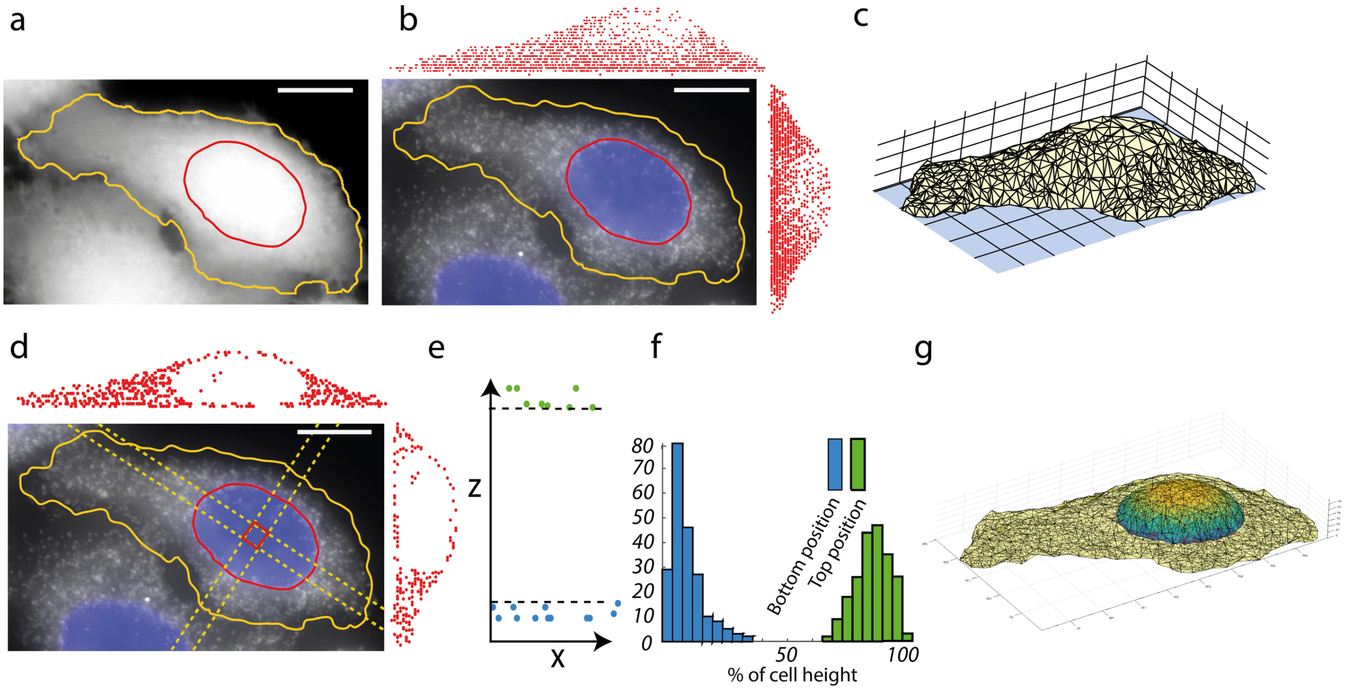

Supplementary Figure 1. Obtaining a realistic 3D cell. a) *HeLa cell with CellMask™ staining. Shown is a maximum intensity projection along z. 2D segmentation of the cell is displayed in yellow, nuclear segmentation in red.* ***b)*** *HeLa cell from a) with smFISH against GAPDH. Projections of the 3D mRNA detection results with FISH-quant are displayed above the image for the xz-plane and on the right for the yz-plane.* ***c)*** *3D volume of the cell obtained by triangulation of the detected GAPDH mRNAs.* ***d)*** *HeLa cell with smFISH against GAPDH. mRNA detected in 3D are displayed as in b) but only for mRNAs within the dashed, yellow rectangles.* ***e)*** *For each cell, mRNAs inside the central part of the nucleus (red box in d) are extracted and are classified into two groups (above (blue points) and below (green points) the nucleus).* ***f)*** *Histogram of the z-positions normalized by the height of the cell for the two categories.* ***g)*** *3D polygon of the cell reconstructed with GAPDH mRNA detection and 3D nucleus modeled by a semi ellipsoid using parameters extracted from f). Scale bars 10 µm.*

We next wanted to obtain an accurate description of the 3D nuclear shape. Here, we used our observation that *GAPDH* – despite its high expression level – is largely excluded from the nucleus (Fig S1d). By manual inspection of the data, we observed that the nucleus is relatively flat at the bottom and round at the top, and can thus be approximated by a semi-ellipsoid in 3D. We estimated the semi-axis of the ellipsoid in xy by fitting a 2D ellipse on the 2D segmentation of the nucleus from the DAPI channel. To determine the average height of a nucleus, we analyzed the 3D positions of mRNAs located at xy-positions in a small squared region close to the center (Fig S1d-e). For this, we first removed occasional detections inside the nucleus (potentially corresponding to mRNAs not yet exported to the cytoplasm) by ignoring mRNAs with fewer than 3 detections present in the same z-slice. The remaining mRNAs where classified into two groups using k-means clustering on the z-coordinate, yielding the bottom and top group (Fig S1e). Finally, we defined the lower position of a nucleus as the highest z-value from the bottom group, and the upper position as the lowest z-value from the top group (Fig S1e). We estimated these two parameters as a percentage of the total height of the cell for each cell to account for differences in cell

height. When averaging these estimates, we determined that the bottom-position of the nucleus is at 7% of the cell height, and a top position at 88%. For each cell, we then simulated a nucleus according to these parameters.

Finally, we visually checked every cell to detect potential segmentation errors leaving us with a library of more than 300 cells. For each of these cells, we have the 3D shape of the nucleus, the 3D shape of the cell (Fig S1f).

1.3. SIMULATIONS OF REALISTIC 3D SMFISH IMAGES

We next implemented a way to simulate realistic smFISH images. The individual mRNAs are smaller than the diffraction limit[11] and can hence be described by a Point Spread function (PSF) (Fig S2a). Different mathematical models to simulate realistic PSFs for different optical systems have been developed and many are available in the ImageJ plug-in PSFGenerator[12]. We used the Richard and Wolf model with a wavelength of 550 nm and a NA of 1.4. In agreement with published literature[11,13], we found that the simulated PSFs were slightly smaller than experimental PSF. To correct for this difference, we increased the pixel-size in xy by 1.3 and z by 1.6, respectively.

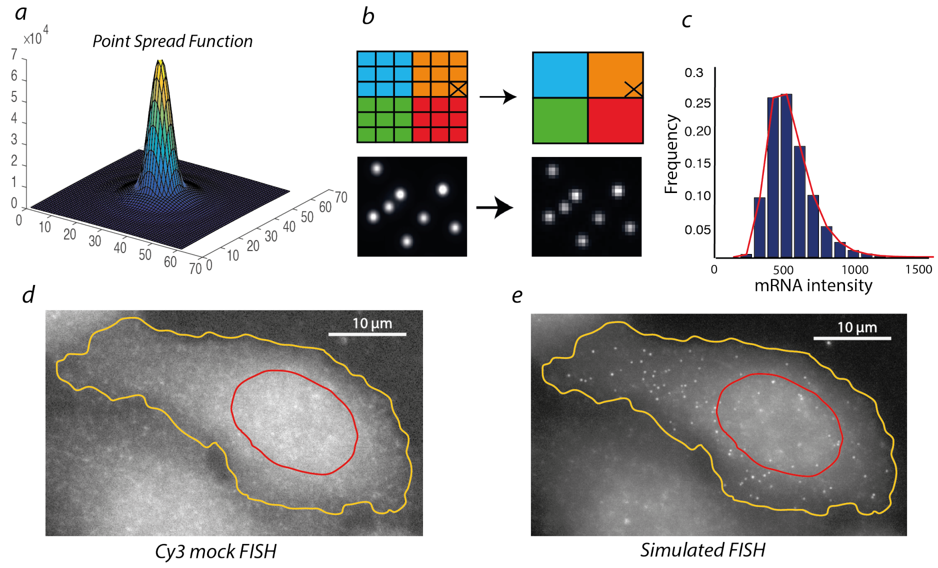

Supplementary Figure 2. Simulating realistic smFISH images. ***a)*** *3D plot of PSF simulated with PSFGenerator.* ***b)*** *Illustration of the subpixel placement of mRNAs. mRNAs are first placed on a large grid (left) which is then binned into a smaller one (right).* ***c)*** *mRNA intensity distribution extracted from experimental data (KIF1C). The histogram is fitted with a skewed normal distribution (red line).* ***d)*** *Image of HeLa cell with negative control experiment of smFISH used in the simulations (scale bar 10µm)* ***e)*** *Simulated FISH image with random localization of mRNA. Individual mRNAs are (1) placed as illustrated in (b), (2) normalized according to the intensity distribution shown in (c), and (3) added to the background smFISH image in d). We show image (d) with a different scaling than image (e) in order to highlight the aspect of the background signal.*

In order to reach sub-pixel localization and heterogeneity in pixel-intensity of each mRNA, we simulated the PSFs on a finer grid than the actual image and then binned the simulated image to obtain the final resolution (Fig S2b). In real smFISH experiments, a large variation in signal intensity can be observed, which is due to probe accessibility and difference in hybridization efficiency[11]. We modeled this variability by fitting the observed intensity distribution from real experimental data (Fig S2c). For each mRNA, a random intensity is drawn from this distribution. 3D images are now simulated by placing individual mRNA molecules in the available 3D cellular space as defined by the 3D cellular shape. Specific mRNA localization can be obtained by enforcing simple rules as explained further below. The

final image is then obtained by binning the higher-resolution image of the individual molecules. A prominent feature in real smFISH images is the non-specific background stemming from non-specific binding of the FISH probes[9]. We considered this in our simulations by using a realistic FISH background from the mock FISH experiment. The final simulated image is thus obtained by adding the simulated mRNA localization image to the realistic background image (Fig S2d).

1.4. SIMULATION OF DIFFERENT MRNA LEVELS

One of the key parameters in the simulation of realistic FISH images is the expression level and its variation from cell-to-cell. Even if these variations are currently not fully understood, it has been recently shown that one of the principal regulators of gene expression is the cellular volume[8,14]. More precisely, many genes tend to maintain a constant mRNA concentration and larger cells have thus more mRNAs. In our simulations, we can define a certain target mRNA concentration, and for each cell the actual number of mRNAs was determined based on this concentration. To add further variation, we added a Poisson noise term with zero mean and standard deviation of 10 (i.e. $\varepsilon=100 - \eta(100)$, where η is drawn from a Poisson distribution with mean and variance 100). We simulated four different scenarios, ranging from low expressing cells to highly expressing cells (Fig S3). Having these different expression levels either separately (Fig S3a-d) or pooled together (Fig S3e), is essential to develop robust methods to infer mRNA localization patterns without influence of mRNA levels.

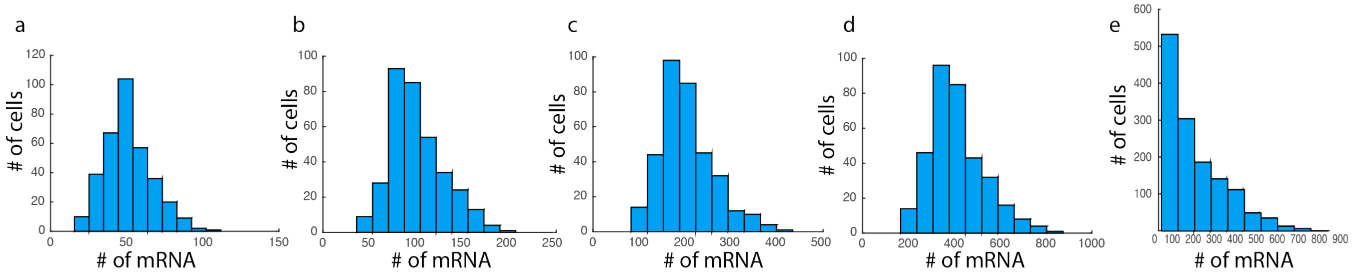

Supplementary Figure 3. Simulating different expression levels. a-d) *Histogram showing the distribution of mRNA per cell for different simulated densities. Average mRNA number per cell is 40 in a), 100 in b), 200 in c), and 400 in d).* ***e)*** *Distribution of mRNA number per cell when pooling a)-d).*

1.5. SIMULATION OF DIFFERENT MRNA LOCALIZATION PATTERNS

Manual inspection of our experimental data shows a number of different mRNA localization patterns (see **Supplementary Note 5** for examples). We therefore developed sets of rules that were capable of reproducing these patterns. In addition, we implemented another set of rules resulting in patterns, which are difficult to detect manually, but which can still make sense from a biological point of view. Each pattern can be described by 1 to 3 different parameters, which control how extreme a pattern will be. For each pattern, we determined 3 different degrees – weak, moderate, and strong. The moderate pattern corresponds to a pattern that could be typically observed in a cell.

For most patterns - and unless indicated otherwise - a parameter ***p*** specifies the proportion of mRNAs showing this particular pattern, while the other mRNAs will be simulated as randomly localized.

Random

Positions are randomly placed in the 3D volume of the cell. Positions inside the nucleus are not considered. Such a simulation process creates a point cloud with complete spatial randomness. Note that this

randomness applies to the available volume in the cytoplasm. As the height of the cell is variable and the nuclear region excluded, this is not identical to a random distribution in 2D in the image.

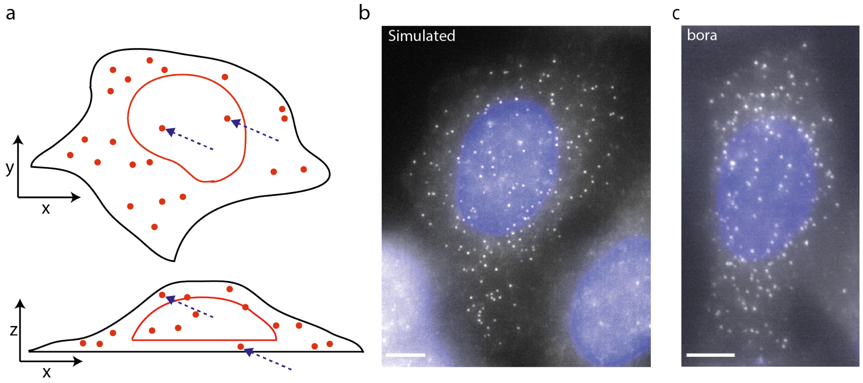

Supplementary Figure 4. ***a)*** *mRNAs are simulated randomly in 3D in the cytoplasm but are excluded from the nucleus. Note that some mRNAs appear to be in the nucleus but this only depends on the view, e.g. the two mRNAs indicated with an arrow appear to be in the nucleus in a xy-view (upper plot), but are above or below the nucleus in the xz-plane (lower plot).* ***b)*** *Simulated image with random mRNA levels.* ***c)*** *real smFISH image with random mRNA localization. Scale bars 5µm.*

Localization with respect to the nucleus

We implemented two different localization patterns with mRNAs in proximity of the nuclear envelope.

Nuclear envelope 3D

An mRNA is considered to be localized at the nuclear envelope if it is below a fixed distance (800 nm) from the envelope (Fig S5a). This pattern is difficult to distinguish from other nuclear patterns by inspection of 2D projections (Fig S5b). The different degrees were simulated by changing the parameter p (low: p = 0.6, moderate: p = 0.7, strong: p = 0.9).

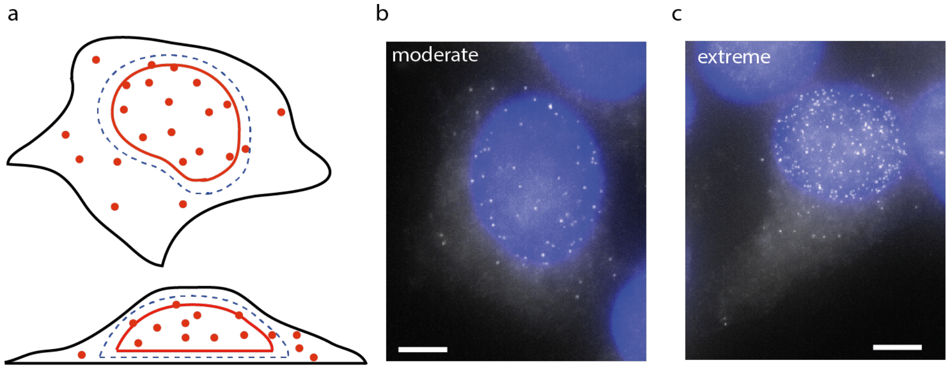

Supplementary Figure 5. ***a)*** *mRNAs are simulated close to the nuclear envelope by placing them between the dashed blue line and the simulated nuclear envelope.* ***b-c)*** *Examples of simulated images. Shown are maximum intensity projections along z. Scale bars 5µm.*

Nuclear envelope 2D

This pattern was motivated by observations from experimental data. We observed localization patterns, where mRNA remain close to the nuclear envelope in 2D, often enriched on one side of the nucleus. The resulting patterns (Fig S6d) looked strikingly different from the simulations shown in Fig S5b-c. For this pattern, RNAs did not localize uniformly on the entire nuclear envelope, but rather selectively to some parts.

mRNA positions for this pattern are simulated using spherical coordinates with the origin at the center of the nucleus. The nucleus is fitted with an ellipse to get the two main axes (Fig S6a). The *polar angle* is picked from a normal distribution centered at one of the four possible nucleus axes directions with a fixed standard deviation. We choose the standard deviation such that the mRNAs localize at one half of the

nucleus, which correspond to the observed pattern. The *azimuth angle* is picked from a normal distribution with a low mean to force the mRNA to locate at the periphery of the nucleus. The radius is picked so that the mRNA is closer to the nuclear envelope than a fixed distance (800nm). The three different degrees of the pattern were simulated with different values of the parameter p (low: p = 0.5, moderate: p = 0.6, strong: p =0.7; Fig S6b-c).

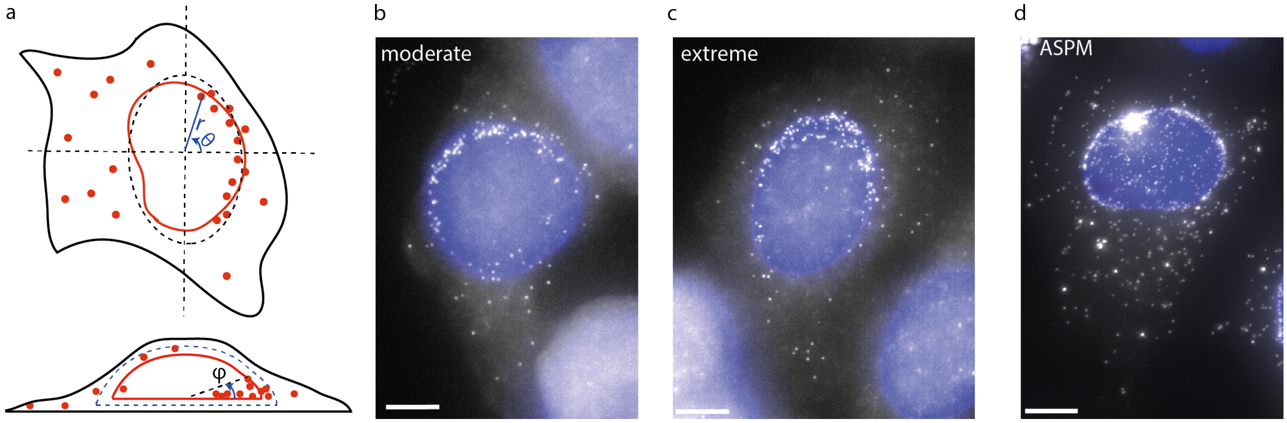

***Supplementary Figure 6**. **a)** mRNAs are simulated close to the nuclear edge by enriching them (i) on one side of the nucleus, and (ii) not covering the entire nuclear envelope in 3D. **b-c)** Examples of simulated images. **d)** Experimental data showing. Note that the bright spot in the nucleus is presumably an active site of transcription. Scale bars 5µm.*

Localization with respect to the cell membrane

For localization at the cellular membrane, we implemented two different patterns.

Cell membrane 3D

An mRNA is considered to localize at the cell membrane if it is below a fixed distance (800 nm) from the cell membrane in 3D. The three different degrees of the pattern were simulated with different values of parameter p (weak: p = 0.7, moderate: p = 0.8, strong: p =0.9). An intriguing feature of this pattern is that it is very difficult to be spotted from 2D projections, which can be appreciated from Fig S7: it is easy to confound this pattern with random localization inside the cytoplasm

***Supplementary Figure 7**. **a)** mRNAs are enriched along the cellular membrane. Dashed lines in upper panel show cross-section that is shown in lower panel. **b-c)** Examples of simulated images as in Fig 4. Scale bars 5µm.*

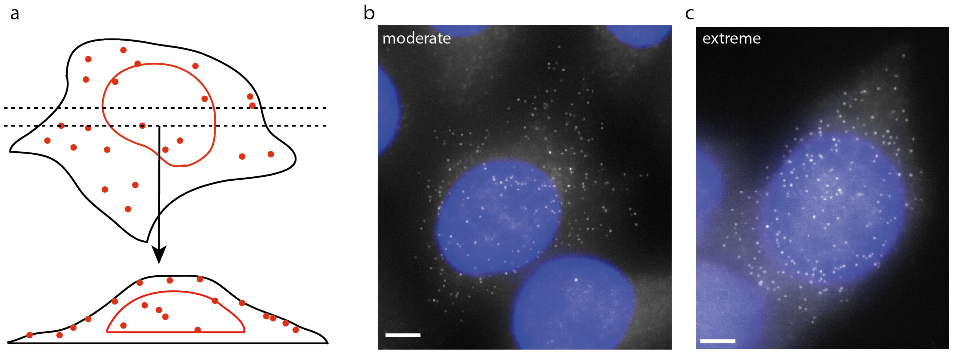

Cell membrane 2D

Here, the mRNAs show localization towards the edge of the cell as seen in 2D. Positions are simulated with polar coordinates. The polar angle is picked from a uniform distribution between 0 and 2pi, and the radius is chosen such that the mRNAs are closer to the cell membrane than a fixed distance (800 nm). The

three different degrees of the pattern were simulated with different values of parameter p (weak: p = 0.4, moderate: p = 0.5 strong: p =0.6)

***Supplementary Figure** 8. **a)** mRNAs are enriched along the cellular edge. mRNAs are placed between the dashed blue line and the cellular outline. **b-c)** Examples of simulated images. Scale bars 5μm.*

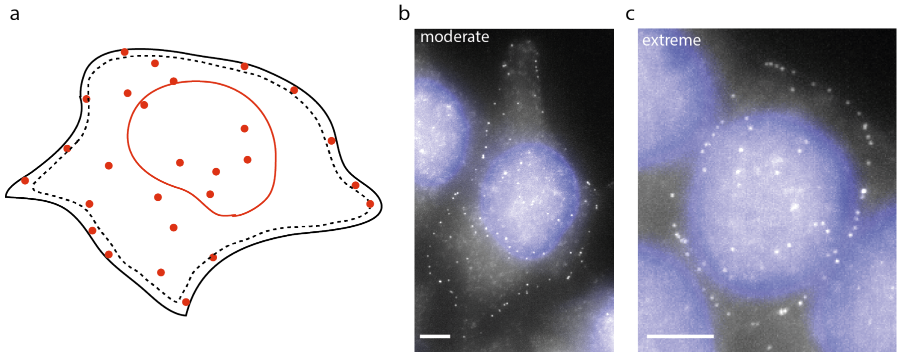

Polarized mRNA localization

In this pattern, mRNAs occupy preferentially a certain part of the cytoplasm. The cell is fit with an ellipse in order to get the two main axes. mRNA positions are simulated using polar coordinates with origin at the centroid of the cell. The polar angles are drawn from a normal distribution centered at one of the four possible main axes with a standard deviation determined by the user. The smaller this standard deviation is, the stronger the pattern will be. The three different degrees of the pattern used in our study were simulated with different values of parameter σ (weak: $\sigma = \pi/10$, moderate: $\sigma = \frac{3\pi}{10}$, strong: $\sigma = \pi/2$).

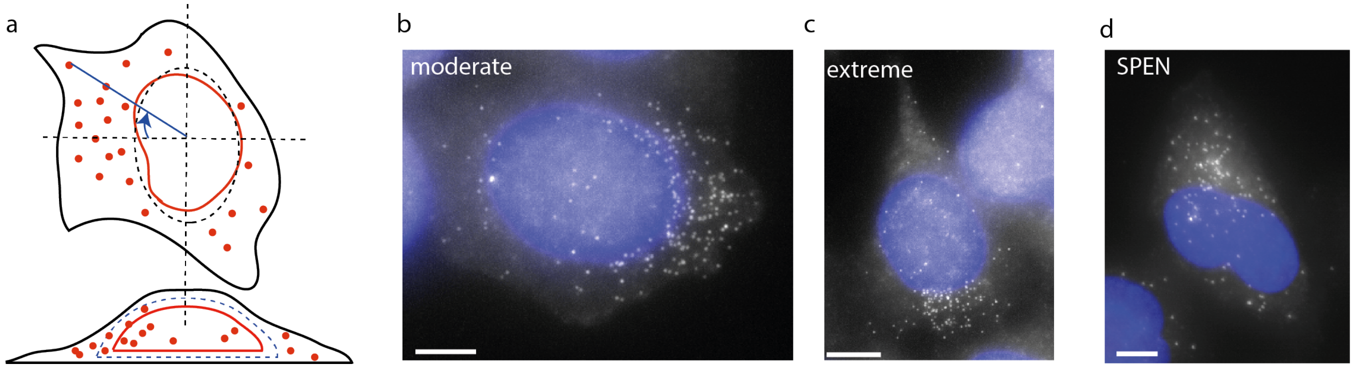

***Supplementary Figure** 9. **a)** mRNAs are polarized, e.g. preferentially in one part of the cytoplasm. **b-c)** Examples of simulated data. **d)** Experimental data showing a similar localization pattern. Scale bars 5μm.*

Cellular extension

We found that some mRNAs are enriched in cellular extensions. There is no formal definition of "cellular extension", but it is straightforward to give examples for extensions from image data. Hence, in order to model this pattern in a biologically meaningful way, we decided to annotate cellular extensions in our data set and to simulate this pattern as an accumulation in these annotated locations.

More precisely, we extracted the annotated part of the cell border and we calculated the distance to the center of the cell. We considered the pixel on the border with largest distance to be the tip of annotated cellular extension. In some cells, several possible extensions were annotated. For the simulation, we randomly pick a maximum of three extensions for a given cell. We then simulated this localization pattern as uniform RNA distribution around the cellular extension tip within a fixed radius r_{tip} (2000nm). The user specifies the ratio R between RNA densities at the cellular extension and the rest of the cytoplasm respectively. The simulation then proceeds as follows: first, a number of RNA locations are drawn from a uniform distribution inside the cells. Second, additional mRNAs are localized in the cellular extensions

in order to satisfy the density ratio. The three different degrees of the pattern were simulated with different values of parameter R (weak: $R = 10$, moderate: $R = 15$, strong: $R = 20$)

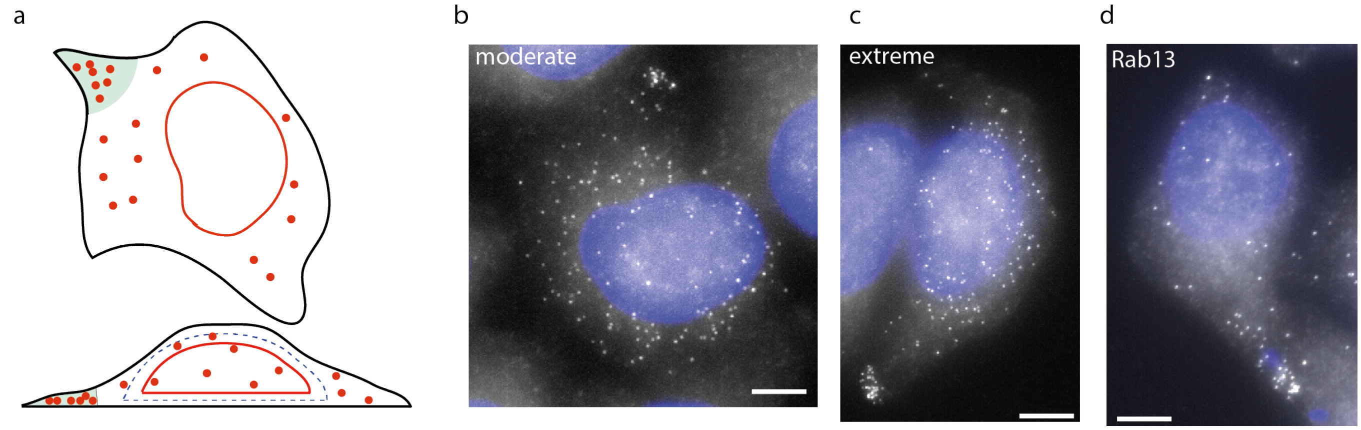

***Supplementary Figure 10**. **a)** mRNAs are enriched in manually annotated cellular extensions (indicated in green. **b-c)** Examples of simulated images. **d)** Experimental data showing a similar localization pattern. Scale bars 5µm.*

mRNA foci

For some genes, the corresponding transcripts aggregate and thus form foci, i.e. small areas of very high RNA density, where single RNA molecules can no longer be resolved (see also Note 2). We extracted from experimental data (*DYNC1H1*) the distribution of foci number per cell and the number of mRNAs per foci (using the GMM method detailed in Note 2). In the simulation, we choose randomly from these distributions how many foci and how many mRNAs per foci are simulated. The spatial extent of these foci was set to a size-range that visually corresponded to experimental data (radius of the sphere picked between 500-1000 nm). For more details on the simulation on foci, we refer to Note 2. The foci were randomly placed in the cytoplasm in 3D. Cells simulated with the parameters extracted from *DYNC1H1* were considered as strong. For the moderate pattern, we reduce both the number of foci and the number of mRNAs per foci by 25% compared to the strong case. For the weak pattern, we reduced both numbers by 50%.

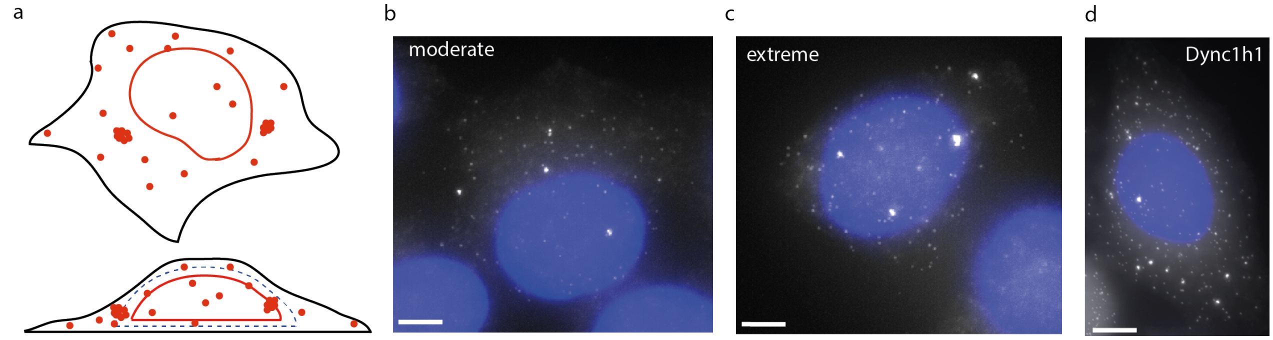

***Supplementary Figure 11**. **a)** mRNAs are enriched in small foci. **b-c)** Examples of simulated images. **d)** Experimental data showing a similar localization pattern. Scale bar 5µm.*

1.6. SUMMARY

In this section, we described how we simulated realistic smFISH images. First, we used experimental data to establish a dataset of more than 300 cells with realistic 3D shapes of both the cell membrane and nuclear

envelope. Second, individual mRNA molecules are modeled with realistic shape (sub-pixel PSF) and intensity (based on measured intensity distributions) and are added on measured smFISH background. Third, mRNA levels were simulated with four different densities to obtain different expression levels. Fourth, motivated by experimental data, we implemented 8 different mRNA localization patterns, described by a set of parameters (Table S1).

For each pattern (except random), we defined three different levels - strong, moderate, and weak. For each condition (one expression level and one localization pattern), we simulated 100 different cells. In total, we simulated 8800 cells representative for a large range of parameter combinations, namely the strength of the pattern and the number of RNAs.

**MAIN Parameters**
**RNA density (**mean and Poisson noise**)**
**Pattern strength** (1 for each pattern): how strong a pattern will be<br>• **Nuclear envelope 3D**: percentage of localized RNAs<br>• **Nuclear envelope 2D**: percentage of localized RNAs<br>• **Cell membrane 3D**: percentage of localized RNAs<br>• **Cell membrane 2D**: percentage of localized RNAs<br>• **Cellular extension**: enrichment in extension compared to cytoplasm<br>• **Polarized**: standard deviation of polarization angle<br>• **mRNA foci**: modulating factor for reference pattern

**Additional fixed parameters**
**Nuclear envelope 3D**: distance to membrane (default 800nm)
**Nuclear envelope 2D**: distance to membrane (default 800nm)
**Nuclear envelope 2D**: phi angle (azimuth angle in spherical coordinate of the position of the mRNAs with a default mean of 0.15 rad and a sigma of 0.1 rad)
**Nuclear envelope 2D**: theta angle (polar angle in spherical coordinate of the position of the mRNAs with a default sigma of 1 rad around the mean defined by the nucleus orientation)
**Cell membrane 3D**: distance to membrane (default 800nm)
**Cell membrane 2D**: distance to membrane (default 800nm)
**Cell extension**: maximum distance from cell membrane where RNAs will be placed.
**Polarized:** percentage of localized RNAs
**RNA foci**: number of RNAs in foci for reference pattern (mean, sigma, kurtosis, skewness)
**RNA foci**: number of RNAs per cell for reference pattern (mean, sigma, kurtosis, skewness)
**RNA foci**: size range of foci (min/max) for reference pattern

Supplementary Table 1*. List of parameters used to simulate images. The main parameters can be used to introduce heterogeneity in the data by influencing how many RNAs are placed in a cell (RNA density) or how strong a pattern will be (pattern strength). The fixed parameters are not altered and define the general behavior of certain patterns.*

Supplementary Note 2: Quantification of mRNA foci

2.1. OVERVIEW

In smFISH, several fluorescent probes target the RNA of interest, and individual molecules appear as bright spots under the microscope. The RNAs of some genes are non-randomly distributed and form dense foci of individual molecules (Fig S12a shows an example of mRNA foci in the cytoplasm, for another example of nuclear foci of lncRNA see Cabili et al.[15]).

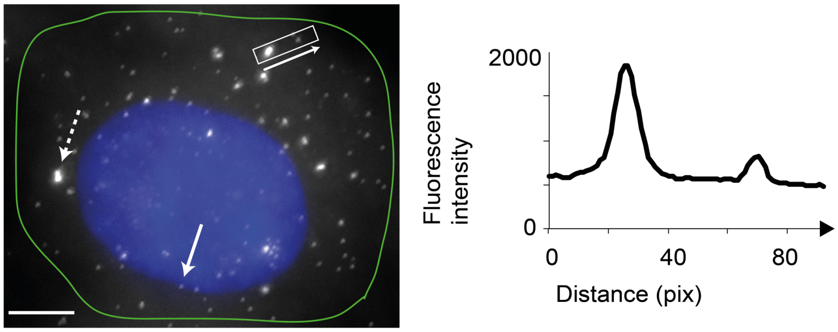

Supplementary Figure 12. *FISH image of a cell displaying both individual mRNA molecules (example highlighted with a white full arrow) and mRNA foci (white dashed arrow). Inset shows intensity profile through the region indicated by the white rectangle along the arrow. Nucleus is stained with DAPI and shown in blue. Scale bar 5 µm.*

The accurate description of these foci as point clouds of mRNA molecules is important to study RNA localization, but adequate quantification methods – especially for large data sets - are currently missing. Here we describe a method based on Gaussian Mixture models (GMM), specifically tailored to smFISH images. We carefully validate the accuracy of our approach with realistic simulations. We further provide a variant of this method with ten to hundred-fold decrease in computation time, with only a modest loss of accuracy. Such methods are particularly important for High Content Screening, where tens of thousands of experiments need to be analyzed in a single project.

2.2. IMAGING AND DETECTION OF INDIVIDUAL MRNA MOLECULES

The image of an mRNA – such as many other labeled biomolecules - is diffraction limited in size, and can therefore be described by the point spread function (PSF) of the microscope. As a simplification, a PSF can be described by a Gaussian function [13]. The intensity of each voxel I_{ijk} is then described as a Gaussian integrated over the voxel volume:

$$I_{ijk} = B + \frac{1}{V}\int_{x\in Voxel(ijk)} A\, G(x,x0)dx, \qquad (1)$$

where $G(x,x0)$ is a Gaussian centered in $x0 \in \mathbb{R}^3$

$$G(x,x0) = e^{\frac{-(x-x0_x)^2+(y-x0_y)^2}{2\sigma_{xy}}} e^{\frac{-(z-x0_z)^2}{2\sigma_z}}, \qquad (2)$$

where B is the image background, A the amplitude of the Gaussian, V the volume of the voxel, $x0$ denotes the center coordinates in xyz, σ the standard deviation in xy and z. The integral in Eq. (1) is (up to a simple normalization) the well-known error function, implemented in almost all important statistical software packages (function *erf* in Matlab).

Detection of individual mRNA molecules can be performed with standard spot detection tools such as FISH-quant[11], Aro[16], TrackMate[17]. The basic workflow encompasses different steps and is performed in 2D or 3D. The typical analysis steps in our software tool FISH-quant[11] are (1) A filtering step with the goal to facilitate spot detection. Among the most commonly used filters are the Laplacian of Gaussian

(LoG), providing an approximation of the second derivative of the image, or the Difference of two Gaussian filters, calculating the difference between two filtered versions of the image with different bandwidths, the larger one removing background and the smaller enhancing the signal of small objects. (2) The detection of spot candidates. This is commonly done in the filtered images by either determining its local maxima (local maxima method) or by applying a threshold and identifying regions of connected pixels in the thresholded image (connected component method). (3) Fitting a 3D Gaussian function to the spot signal in the raw image. This workflow yields the number and positions of individual mRNA molecules but it requires that these molecules are sufficiently spatially apart to be readily distinguished.

2.3. MRNA FOCI AND GAUSSIAN MIXTURE MODEL

We define mRNA foci as a strong local concentration of individual mRNA molecules where the signal of individual molecules cannot be readily distinguished with the above described detection approach. Mathematically we can describe such a foci as an integral over a sum of Gaussians.

$$I_{ijk} = B + \frac{1}{V}\int_{x\in Voxel(ijk)} \sum_{l=1}^{K} A_l G_l(x, x0)\,dx \qquad (3)$$

where G is the Gaussian function from Eq. (2), and K denotes the number of Gaussians. For a given number K of Gaussians, we can estimate the other parameters (locations, amplitudes and background) by solving the following optimization problem:

$$\min_{\boldsymbol{\theta}} \sum_{ijk\in R}(I_{data,ijk} - I_{ijk}(\boldsymbol{\theta}))^2 \qquad (4)$$

$$\boldsymbol{\theta} = (\boldsymbol{x0}, \boldsymbol{A_l}, \boldsymbol{B})$$

where R is the set of voxels in the image, I_{data} is the actual (observed) image, and $I_{ijk}(\theta)$ is the reconstructed voxel *ijk*, as defined in Eq. (3); the summation is performed over all voxels forming the image. We note that the standard deviation of the Gaussians is defined by the PSF and not subject to optimization. The image analysis task now consists in estimating the number *K* of Gaussians, their location and amplitudes for a given mRNA foci.

Thomann et al. [18] proposed a procedure to infer K, which we used as a starting point for the development of our method. Importantly, the amplitudes of the Gaussian are here also subject to optimization. The approach consists in successively adding components (K=2,3,…) and placing them optimally by solving the optimization problem in Equation (4). After each addition, a test based on χ^2 statistics decides whether to stop. This statistical stop criterion is necessary, because the Gaussians in this approach can have arbitrary amplitudes, and hence a higher number of Gaussians always approximates the original data better in terms of error.

We argue that in smFISH images, each mRNA is targeted by the same number of fluorescent probes and has therefore a comparable intensity. Variations nevertheless occur due to differences in hybridization efficiency and bleaching of dyes. But we can estimate a median intensity from experimental images of isolated single mRNAs (Fig S12c). In this approximation, identifying the parameter K of the GMM is equivalent to the estimation of how many mRNA molecules of median intensity best describe the mRNA foci. Since each added Gaussian has the same amplitude, we can simply choose the number of components that minimizes the error after optimizing their positions. As the number of Gaussians is increased, the error term will first decrease, then increase again. We thus stop adding new components when the error term increases. In summary, by fixing the amplitudes of the Gaussian rather than estimating them, we can replace the statistical test procedure by a minimization; the whole algorithm can thus be written as a nested optimization problem:

$$\min_{K} \min_{\theta} \sum_{ijk \in R} \left(I_{data,ijk} - I_{ijk}(\boldsymbol{\theta}) \right)^2 \qquad (5)$$

Before carefully validating this proposed approach, we describe next the data we simulated for benchmarking the methods.

2.4. SIMULATED BENCHMARK DATASETS

For the validation of the algorithms, we generated simulation data of mRNA foci at different levels of complexity. We defined a permissive volume in 3D as a randomly oriented ellipsoid with fixed half-axes (a=1500nm, b=1500nm, c=1200nm), in which a defined number of mRNA molecules were placed at random locations (Fig S13a). Individual mRNA molecules are simulated as described in Supplementary Note 1 with a realistic intensity distribution (Fig S13b) and sub-pixel placement (Fig S13c). To obtain a realistic background, the simulated image can be added to the experimental image of a cell in which no mRNA was expressed to obtain the final image (Fig S13d).

Here, we generated two datasets in order to study the methods in detail:

(1) One data set without background (by omitting the last step explained above) that allows us to study how well the decomposition schemes can work in principle on ideal images. We refer to this data set as DS1.

(2) One data set where we have added a background resulting from non-specific smFISH probes (as described above) in order to simulate realistic data. We refer to this data set as DS2.

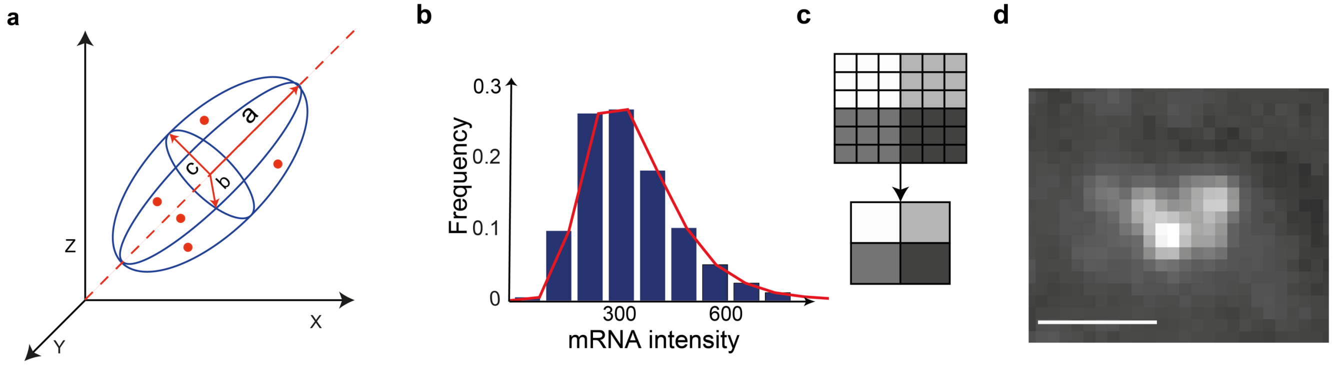

Supplementary Figure 13. Simulated images of mRNA foci. a) *3D ellipsoid defines permissive position for mRNA placements (red dots).* ***b)*** *Intensity distribution of individual mRNAs from experimental data.* ***c)*** *Image is simulated on a pixel-grid three times finer than the actual pixel size. Final image is obtained by binning.* ***d)*** *Example of simulated foci with 8 mRNAs. Shown is a maximum intensity projection along Z. Scale bar 1 μm.*

2.5. RESULTS

Maximal-residual-initialization for new Gaussians

At each iteration of the GMM, a new component has to be placed (Fig S14a). In the original algorithm[18], each position of the preceding iteration was doubled once as an initialization of the center of the next Gaussian component to be placed. Out of these different initializations, the best fit was selected. This choice is not so promising in a case where Gaussians are known not to be at the same place (as it is the case for molecules). Indeed, it seems more logical to initialize the new component such that it has a good chance of explaining the so far unexplained part of the signal. In addition, the traditional approach also requires a large number of fits ($N = 1 + \frac{K(K-1)}{2}$) which grows quadratically with the number of components

K. Here, we propose to initialize the center of the newly added Gaussian at the location $(i_{new}, j_{new}, k_{new})$ where the difference between model and image become maximal:

$$(i_{new}, j_{new}, k_{new}) = argmax(I_{ijk} - I_{data,ijk}) \qquad (6)$$

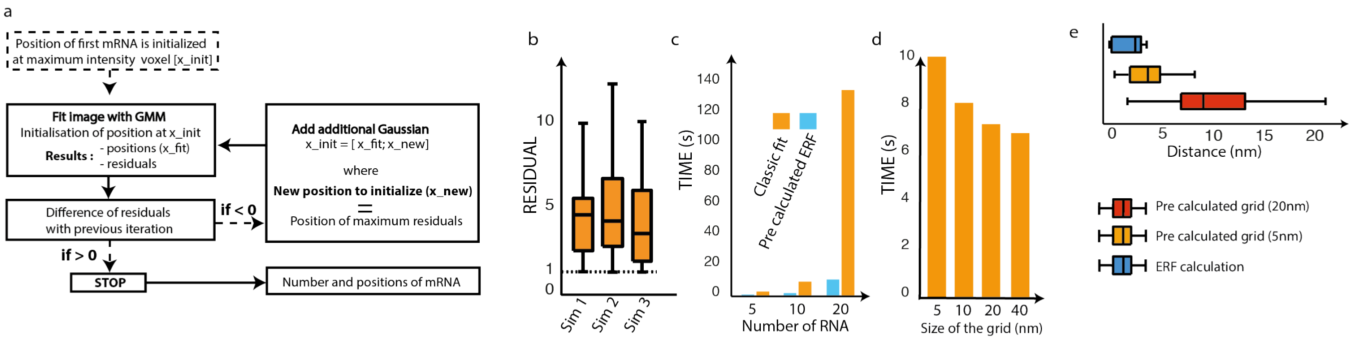

Supplementary Figure 14. ***a)*** *Flowchart of the iterative GMM algorithm.* ***b)*** *Boxplot of residuals from fit with randomized initialization positions normalized by residual from fit with iterative GMM. A value below one means that a random initialization led to a better fit than the iterative GMM. The boxplot shows the median and the Q1 and Q3 quartile. The whiskers go until 1.5*(Q3 – Q1) from Q1 and Q3. Outliers are not shown.* ***c)*** *Comparison of computation time when evaluating the erf function using exact coordinates (orange bars) and when using a lookup table based on approximate coordinates (blue bars).* ***d)*** *Comparison of computation times for different sizes of the pixel grid used to approximate coordinates for a foci with 20 molecules.* ***e)*** *Localization accuracy (defined as distance between estimated and known location of individual mRNA molecule) for single spot detection with explicit calculation of erf calculation, and pre-calculation with two different grid sizes.*

The objective function (5) is not convex in the parameters, and therefore, we are not guaranteed to find the global minimum of (5) and in particular, we cannot exclude that the initialization has an impact on the final result. In order to test whether our iterative GMM algorithm is capable of identifying the true number of RNAs in practice and to investigate the impact of the initialization on the quality of the result, we applied the algorithm to the set DS1 of simulated images described in 2.4. We estimated the size of a median mRNA by fitting simulated isolated mRNA molecules. This analysis yields the estimated number *K* of Gaussians and also the residuals of this fit. We then randomly determined *K* initialization positions and ran the GMM for each of these initializations. We repeated this process 100 times and determined the residuals for each run. When comparing the residuals of the iterative GMM to the randomly initiated GMM (Fig S14b), we found that none of the random initialization resulted in a better fit, suggesting that our algorithm managed to find the global minimum of the objective function.

Faster computation by pre-calculation of Gaussian

Even after reducing the number of initializations for the fit, the algorithm is slow for larger foci and can take several minutes (Fig S14c, orange bars). Such a processing time is prohibitive for large-scale studies. When evaluating computation times, we found that the calculation of the error function to evaluate Eq. (1) (as implemented by the Matlab function *erf)* took 95% of the processing time. This function evaluates the same function each time, but for different positions. We therefore pre-calculated this function on a fine pixel-grid (e.g. 5x5x5nm), providing a 3D matrix which can be used as a look-up table. We modified the objective function of the GMM (Eq. (2)) such that the algorithm uses this look-up table, rather than solving the *erf* function explicitly. The finer grid guarantees that sub-pixel placements are possible, and the final reconstructed image was obtained by binning (akin to the simulations described in section 2.1). Applying this approach to foci leads to ten-fold speed-up in computation time (Fig S14c, blue bars). This speed-up depends only weakly on the size of the pre-calculation grid (Fig S14d). It is therefore preferable to stick to the finer grid. We next tested the accuracy of this method compared to the implementation where *erf* is evaluated each time. Here, we simulated individual mRNA molecules (without background)

and detected them with either approach, obtaining comparable localization precision when using a very fine pre-calculation grid (Fig S14e). Increasing the grid size, leads - as expected - to reduced localization accuracy.

Accuracy of quantification method on realistic images

We next turned to the more realistic case where images contain realistic background signal. Given the sensitivity of the decomposition scheme to the presence of background, we argued that it was necessary to preprocess such images prior to running the GMM in order to: (1) remove background and (2) focus the analysis only on the relevant region. We reasoned that this pre-processing could help to reduce contributions of defocusing patterns of the PSF, which are not described appropriately by the Gaussian function. Indeed, the Gaussian PSF model describes well the central part of the PSF, but it fails to describe the more complex defocusing patterns away from the center. For an individual PSF (or mRNA molecule), this difference is very small. However, when PSFs are stacked above each other, the additive signal of the defocusing pattern becomes stronger and will eventually reach the intensity of individual PSFs. In such a case, the algorithm might place additional Gaussians in this area, leading to an overestimation of the actual number of molecules in this area.

We approximate the image background by blurring the image I_{raw} with a large Gaussian kernel five-times the size of the PSF:

$$I_{filt} = I_{raw} * G_{5\sigma}. \qquad (7)$$

The image I_{filt} is then used for a background subtraction I_{raw}-I_{filt}.

To further reduce the impact of these patterns, we restrict the GMM analysis only on voxels that are as bright as individual mRNAs. Specifically, we obtained 3D connected components by thresholding the prefiltered image with the detection threshold for individual mRNAs. This provides a mask for the GMM analysis, i.e. only the voxels identified in this step will be decomposed by the GMM in the background subtracted image from Eq. (7). We tested our method on images with background (DS2) onto which we placed simulated foci, each with 5 to 50 mRNAs (Fig S15a). The estimates for the number of mRNA molecules for all tested concentrations were within 15% of the simulated number (Fig S15b). The slight overestimation in mRNA numbers likely comes from the above-mentioned defocusing patterns of the PSF. To validate how severe this effect can become, we simulated mRNA foci with no spatial extent, e.g. all mRNAs are placed at the same location. mRNA numbers were overestimated slightly more by the GMM (Fig S15c). However, we did not observe such dense spatially condensed foci (Supplementary Note 1).

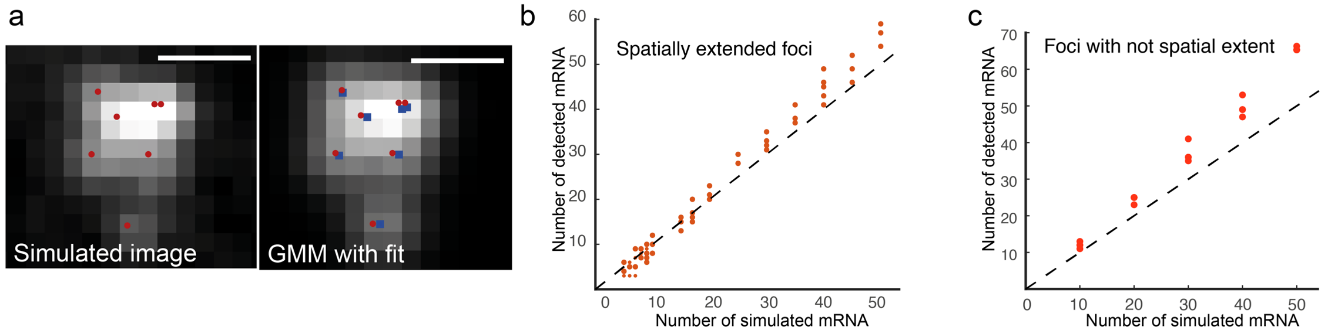

Supplementary Figure 15. Impact of added image background on GMM results. a) *(Left panel): simulated image with true mRNA position (red circles). Images are 3D, but shown after maximum intensity projection along z. (Right panel): results of GMM. with simulated positions (red circle) and fitted positions (blue squares). Images are shown with same intensity scaling (Lower panel): results of GMM*

with position fitting. Scale bars 1 µm. ***b)*** *Number of detected mRNAs as a function of the number of simulated mRNAs.* ***c)*** *Number of detected mRNAs as a function of the number of simulated mRNA for region with no spatial extent.*

Placement without position fitting for faster computation

Despite the reduction in computation time obtained by pre-calculating the *erf* function on a grid, the fitting process is still the bottleneck of our analysis – especially for larger foci. This limits its application to small- or medium-scale data sets. We reasoned that sub-pixel localization accuracy is not necessary for many biological questions, especially if these results are evaluated in the context of an entire cell measuring several tens of µm. We therefore investigated the impact of omitting the fitting step from our algorithm and simply placing mRNA at the position of the maximum residuals (Fig S16a).

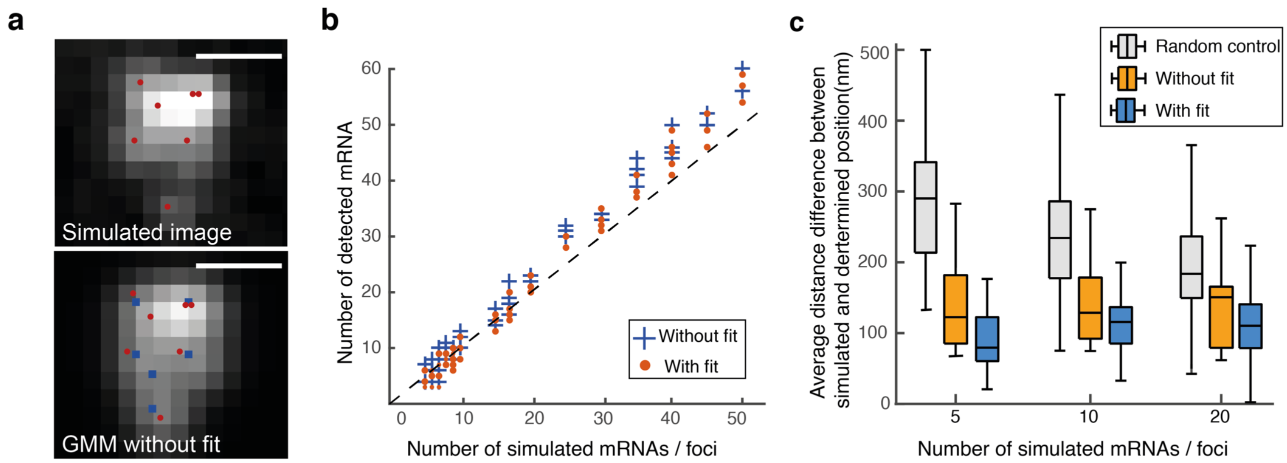

Supplementary Figure 16. Faster GMM by omitting the position fitting. a) *(Upper panel): simulated image with true mRNA position (red circles). Images are 3D, but shown after maximum intensity projection along z. (Middle panel): results of GMM without fitting of positions (blue squares indicate detected positions).* ***b)*** *Number of detected mRNAs as a function of the number of simulated mRNAs. Comparison of analysis with fitted positions (red circles), and without the fitting step (blue crosses).* ***c)*** *Boxplot of the distance between the detected spots and the closest simulated spot. Shown are results for different mRNA numbers with the different quantification approaches. Explanation of boxplots in legend for Fig S14b.*

As expected, this speeds up the computation time by several hundredfold (Table S2). While the estimation of the mRNA number is barely affected (Fig S16b).

Foci size	`erf`: calculated	`erf`: pre-calculated	No fit
10 mRNA	10 s	1.5 s	0.02 s
20 mRNA	130 s	10 s	0.04 s

Supplementary Table 2*. Computation time of the iterative GMM on simulated mRNA foci of different sizes.*

Lastly, we analyzed the estimated positions compared to the known simulated positions (Fig S16c). Not surprisingly, as the density of mRNA increases, the estimated positions become less accurate and closer to a random control. With higher mRNA density, many decompositions of the observed signal are equivalently likely and while the number of RNAs can still be estimated with reasonable accuracy, the positions of the single components (corresponding to multiple molecules) can no longer be distinguished. Also, the positions are less accurately found when omitting the positions fitting step.

Parameter estimation for the isolated spot model from mixture data

Until now, we used the size and amplitude of individual mRNA molecules as a known input parameter for the GMM. One challenge with experimental data is that we do not know the Gaussian function

corresponding to a median mRNA a-priori (Eq. (2)). We therefore tested if we can infer the parameters describing the Gaussian from experimental data in the presence of mRNA foci.

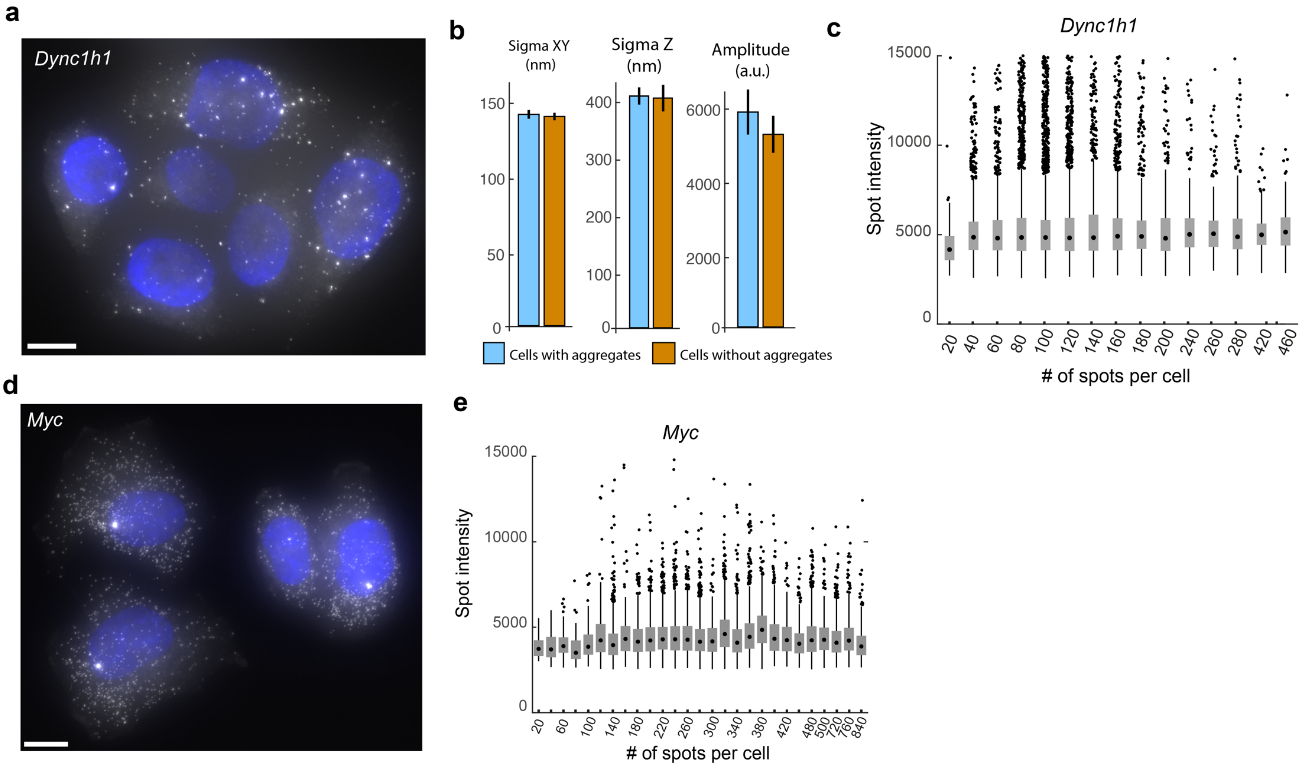

Supplementary Figure 17. ***a)*** *smFISH image against DYNC1H1 shown in white, DAPI in blue.* ***b)*** *Boxplots (explained in Fig 3D) comparing median of estimated amplitude and standard deviation of spots detected in cells with and without foci. Error bars indicate two standard deviations.* ***c)*** *Box plot of spot intensity as a function of the expression level. For each expression level, several cells were pooled together. Explanation of boxplots in legend for Fig S13b.* ***d)*** *smFISH against MYC shown as in a).* ***e)*** *Spot intensities of MYC as a function of expression level shown as in c).*

We speculated that individual mRNA molecules are highly abundant compared to mRNA foci and hence the contribution of foci to median values of the estimates of the 3D Gaussian fit should be negligible. To test this hypothesis, we analyzed data from a gene showing large numbers of very bright foci in most cells (*DYNC1H1*, Fig S17a). We manually split cells in two populations – the ones with and the ones without foci. We then performed a standard spot detection[11] and fitted each detected spot (either mRNA or foci) with a 3D Gaussian function. We found that the median values of the estimated standard deviation and amplitude were comparable for both populations (Fig S17b). This justifies choosing the median values of all fitted spots as an approximation for the individual mRNA molecules. We next tested whether the spot intensity depends on the expression level of the mRNA, and found that this is neither the case for *DYNC1H1* (Fig 6c) nor *MYC*, a gene not showing any foci (Fig S17d-e). This allows us to use parameters either from a single cell or pool results from multiple cells together in case some cells express only few mRNAs hence providing poor statistics. In the actual implementation, the parameters of the Gaussian are thus estimated for each cell independently, except when fewer mRNAs than a user-defined threshold are detected in a given cell (set to 150 mRNAs for this analysis). In this case, the median values of all detected mRNAs in the actual image containing this cell are used.

Application of the automated analysis workflow on simulated data

We next tested how accurate this approach works for simulated data. We simulated cells with 100 randomly distributed mRNA molecules and added two spherical foci with 5-50 mRNAs and a diameter of 500-1000 nm (Fig S18a). The resulting images were analyzed with an automated script that detects all spots, uses the median value of these spots as an approximation of an individual mRNA, and then analyzes all spots with the GMM.

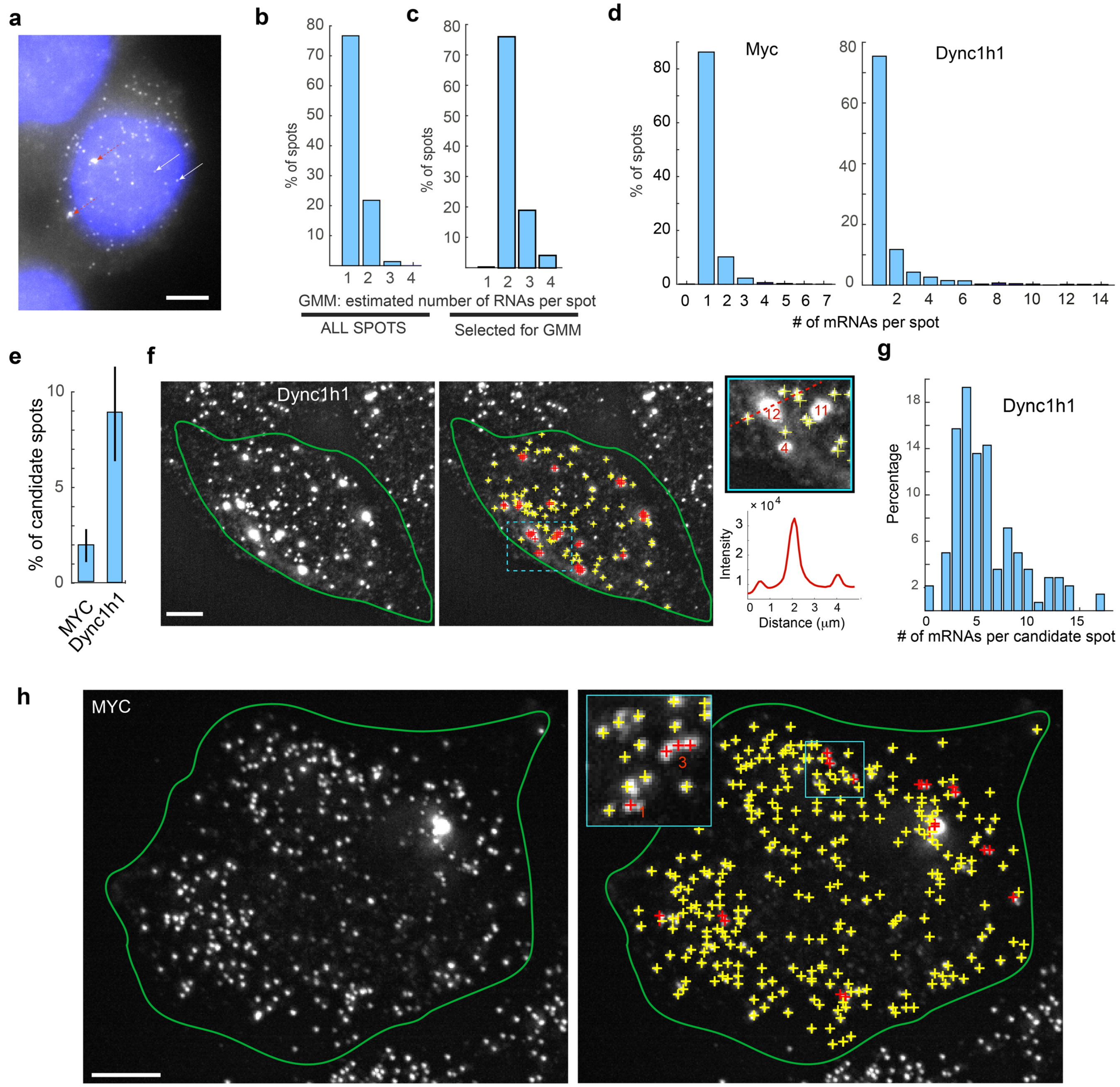

*Supplementary Figure 18. **a)** Simulated cell with 100 single mRNAs and two mRNA foci **b)** Number of mRNAs per detected spot provided by the GMM when applied to all detected spots (the two biggest reconstructed spots corresponding to the foci are not shown). **c)** Percentage of spots that were selected as candidate spots for the GMM based on the number of local maxima and the intensity. **d)** Number of mRNAs*

*per detected spot provided by the GMM when applied to a pre-selection of candidate spots (the two biggest reconstructed spots of each cell are not shown). **e)** Estimated number of mRNAs per detected spot for MYC (left) and DYNC1H1 (right). Error bars indicate two standard deviations. **g)** Percentage of all detected spots selected in the pre-processing as candidates for the GMM **f)** Result of GMM analysis for DYNC1H1. Left image shows background subtracted image. The background image is estimated with a convolution of the original image with a large Gaussian kernel. Green line is manually drawn cellular outline. Middle image shows result of single spot detection (blue crosses) and GMM analysis (red crosses). Zoom-in for region highlighted with yellow rectangle. Numbers in red indicate how many mRNAs per placed by the GMM. Dashed red line indicates position of intensity profile shown intensity plot. **g)** Estimated number of mRNAs per candidate spot for DYNC1H1. **h)** Example of GMM analysis for MYC shown as in h). Scale bars 5 µm.*

Ideally, individual mRNAs will not be separated by the GMM in multiple mRNA molecules, while foci will be. We therefore analyzed how many individual mRNAs are separated by the GMM. We found that 76% of the single mRNAs were not separated in several spots, more than 98% were not separated in more than 2 mRNAs, and more than 99% of the spots were not separated in more than 3 mRNAs (Fig S18b). These separations in multiple mRNA can occur for several reasons. First, the individual mRNAs were simulated with heterogeneous intensity, but will be reconstructed with the properties of a median mRNA. Individual mRNA that are substantially brighter than the median mRNA will then be separated in several mRNAs. Second, some mRNAs can be very close to each other and several mRNA can be detected as one, and then correctly separated by the GMM.

This analysis is time-consuming since every identified spot is processed by the GMM. As a faster alternative, we added a simple pre-processing step selecting a subpopulation of candidate spots for the GMM reconstruction. We empirically determined two criteria for this selection based on intensity and size. These criteria were also validated on experimental data as described below. First, a spot needs to be two times brighter than the median of all spots to undergo the GMM decomposition scheme. Second, only candidates for which there were at least two local maxima inside the candidate region determined by the connected component approach[11] were decomposed by the GMM approach. When applying this pre-processing step to the simulated data, we found that only 8% of the individual spots were selected to be reconstructed by the GMM (Fig S18c), whereas all foci were selected for GMM processing. As before, we estimated the number of mRNAs per candidate with the GMM. Among these 8% of spots (excluding the two biggest of each cell), the majority of spots were separated in 2, and 100% of spots in less than 4. Based on this and given the intrinsic variation of spot intensities, which makes it impossible to differentiate between two very close low-intensity spots and one brighter spot, we decided to add a thresholding step in our analysis in order to reject the results of the GMM when the reconstructed spot is separated in less than 3 mRNAs.

Application of automated analysis workflow on experimental data

We applied our GMM method to real smFISH data. We performed the entire analysis workflow on *MYC* (no visible foci) and *DYNC1H1* (many visible foci). As for simulated data, we first analyzed all identified spots with the GMM. As expected, for *MYC* a large majority of the detected spots (93% of 3478 spots) were not separated into several spots (Fig S18d), therefore correctly identifying them as individual mRNA molecules. Importantly, more than 99% of the spots (3455 of 3478) were separated in at most 3 mRNAs (Fig S18d). This separation of single spots in 2 or 3 mRNAs can be explained by the intrinsic heterogeneity[19] of the smFISH signal, where individual mRNAs are bound by a different number of fluorescent probes, and by individual mRNA molecules that are close by chance. Based on these results, we define a foci to be an accumulation of at least 3 mRNA, as detected by the GMM approach. We then analyzed *DYNC1H1* (Fig S18e). Here, we found that 8% of the detected 1403 detected spots were split by the GMM in more than 3 mRNAs. On average, these foci contained 4.8 mRNAs (Fig S18f).

We then applied the faster analysis pipeline, where spots and potential foci were separated in a pre-processing step detailed above. We found that only 2% of the *MYC* spots were selected for further

processing, while around 9% of the *DYNC1H1* spots were selected (Fig S18g). This is in good agreement with the estimated percentages above. Importantly, visually the identified spot candidates correspond to the brighter foci (Fig S18h). Brighter foci are also separated into larger number of individual spots (Fig S18h), yielding 5.6+/-3.3 mRNAs per foci for *DYNC1H1* (Fig S18i). Moreover, a visual inspection of the automated analysis of *MYC* revealed that the identified candidates for the GMM often correspond to somewhat spatially extended signals stemming from close mRNAs and are then correctly split into a small number of mRNAs, which is lower than the threshold of 3 mRNAs that we defined earlier.

Validation with dual-color smFISH data

As a final validation, we performed dual-color smFISH with two set of probes recognizing the same mRNA (*DYNC1H1*). We synthesize a total of 60 probes, split them into two pools (25 probes labeled with CY3, 35 probes labeled with CY5), and recorded the different channels (Fig S19a). We analyzed each channel separately with the automated workflow described above. We then compared the estimated number of mRNAs for co-localized foci in either channel (Fig S19b). These estimates are highly correlated (Correlation coefficient R = 0.74), confirming the reliability and accuracy of our approach.

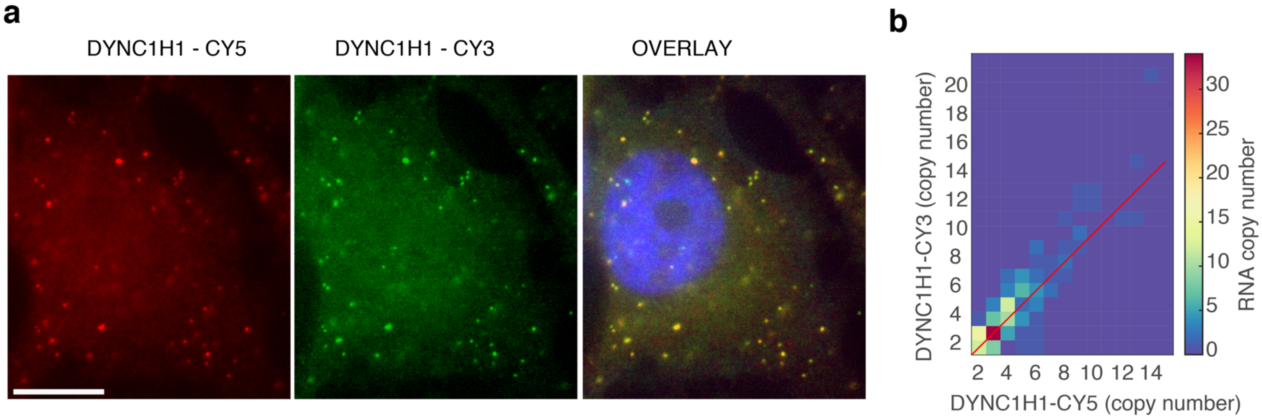

***Supplementary Figure 19. A** Selected focal plane of a HeLa cells with DYNC1H1 mRNA being targeted with two probe-sets labeled with CY5 and CY3. Last plot shows overlay and DAPI stain. Scale bar 10 µm. **B** Quantification results for co-localized foci (N=169). Red line is diagonal with slope 1 indicating perfect correlation.*

2.6. DISCUSSION

We present a carefully validated Gaussian Mixture model adapted to the specific needs to quantify mRNA foci in smFISH images. We found that pre-calculating the 3D Gaussian used to describe individual mRNA molecules substantially speeds up computation time with no loss in accuracy. In general, such a pre-calculation could provide a simple way to accelerate spot fitting, when Gaussians of fixed size and amplitude are used. We further showed that removing the actual position fitting step and placing Gaussians at the positions of the maximum residuals of the previous iteration, results in a very fast algorithm with only slightly reduced localization and quantification accuracy. The loss of accuracy is less pronounced for larger foci, where mRNAs are so dense that individual position can no longer be determined reliably. Here the most relevant quantification parameter is the mRNA count, or equivalently the local mRNA density.

Supplementary Note 3: Description of new localization features

We developed a set of new localization features, which we were able to validate using the synthetic database (See Note 4 for detailed results). Here, we assume that each RNA molecule has been detected and is represented by a single point $p_i=(x_i,y_i,z_i)$ in 3D. Please note that in this section we only provide a definition and justification of these features. Their impact on the classification is investigated in detail in the next section.

3.1. FEATURES DESCRIBING THE 2D DISTANCE BETWEEN MRNAS AND CELLULAR COMPARTMENTS

This family of features is based on the Euclidean distance between individual mRNAs and different parts of the cell (cell membrane, centroid of the cell, centroid of the nucleus, nuclear envelope), calculated in 2D. The distance between an mRNA and a cellular border is defined as the minimum distance between the mRNA and all the points belonging to this border. For a given cell, we compute for each mRNA i:

1. $d_{\text{nuc centroid},i} = \sqrt{(x_{\text{nuc centroid}} - x_{\text{RNA},i})^2 + (y_{\text{nuc centroid}} - y_{\text{RNA},i})^2}$
2. $d_{\text{cell centroid},i} = \sqrt{(x_{\text{cell centroid}} - x_{\text{RNA},i})^2 + (y_{\text{cell centroid}} - y_{\text{RNA},i})^2}$
3. $d_{\text{cell border},i} = \min_{x,y\,\in \text{Cell Border}} \sqrt{(x - x_{\text{RNA},i})^2 + (y - y_{\text{RNA},i})^2}$
4. $d_{\text{nuc border},i} = \min_{x,y\,\in \text{Nuclear Border}} \sqrt{(x - x_{\text{RNA},i})^2 + (y - y_{\text{RNA},i})^2}$

By analyzing all mRNAs in a given cell, we thus obtain 4 distance distributions. We then summarize these distributions by their mean value.

We further computed the quantiles for $\alpha = 0.05$, $\alpha = 0.10$, $\alpha = 0.20$, $\alpha = 0.5$ of the distribution of distances to the cellular membrane, as the mean value might not be sensitive enough to capture localization patterns, for which smaller fractions of the RNAs are localized close to the cell boundary.

5. $Q_{\alpha_{\text{cell border}}} = q_\alpha(\{d_{\text{cell border},i}\})$

These distance values are then normalized to reduce dependency on shape and size of the cell. In a previous study[14], this normalization was performed by division by the square root of the cell area (area normalization). We speculated that this normalization does not take differences in cellular and nuclear geometry into account. We therefore implemented a new normalization, where these distance measurements are renormalized by their expectation under complete spatial randomness (CSR normalization). In order to calculate these expectations, we calculate an average for each of the following distance maps: (1) the geodesic distance inside the cell shape from the centroid of the nucleus, (2) the geodesic distance inside the cell shape from the centroid of the cell, (3) the ordinary distance map, (4) the geodesic distance inside the cell shape from the nuclear region.

To test the impact of this new normalization approach, we selected 14 cells in our cell library with various shapes (Fig S20a). For each cell, we generated images with random mRNA localization and different mRNA levels. We computed the features described above and normalized with both renormalization methods. Ideally, there should be no difference between the renormalized values for the different cells. For some features, we observed larger differences between different cells for the area normalization (see Fig S20b for two examples) suggesting that shape and size of the cell still have a strong influence on the feature values even after this normalization. To quantify this effect more systematically, we calculated the variance ratio VR of the intercellular variance and intracellular variance. With k the number of cells, n_i $(i=1, \ldots, k)$ the number of RNAs inside cell i, y_{ij} the feature value for RNA j in cell i, $\bar{y}_i$ the average feature value for cell i and $\bar{y}$ the average feature value over all cells, we write:

$$VR = \frac{\sum_{i=1}^{k} n_i(\bar{y}_i - \bar{y})^2}{\sum_{i=1}^{k}\sum_{j=1}^{n_i}(y_{ij} - \bar{y}_i)^2}$$

If the cell geometry has a strong impact on the features, the variability between cells of different shapes is large with respect to the variability inside each cell. We can compare the values of this ratio obtained after applying the two normalization schemes. This analysis confirmed the earlier observation, that the CSR normalization reduces the impact of cellular geometry on all feature values compared to the previously used area normalization (Fig S20c), and provides feature values that are less dependent on the shape and size of the individual cells.

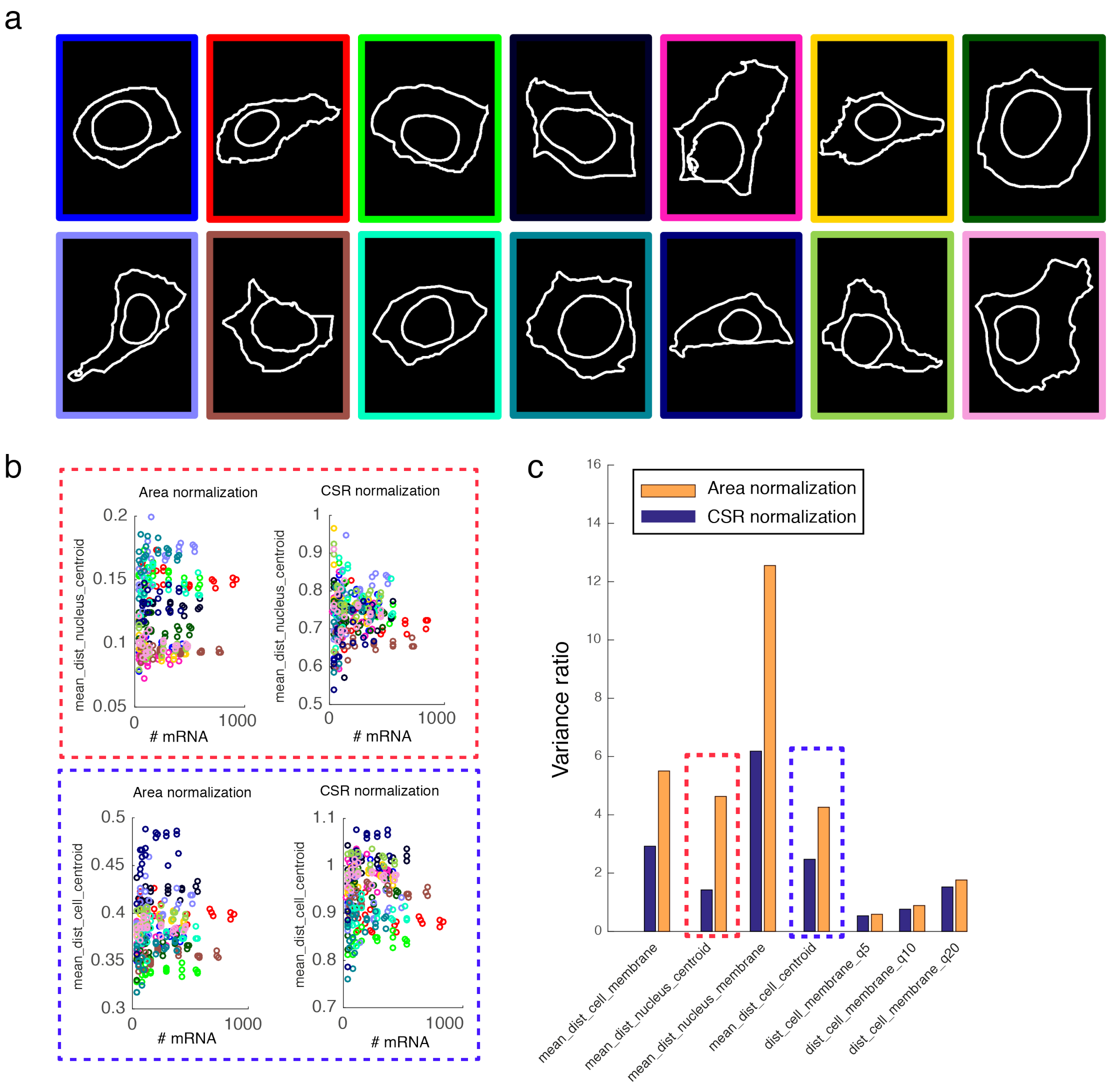

***Supplementary Figure 20**. **Comparison of feature normalization**. **a)** Different cells used to test the two feature normalization approaches. Shown are the 2D outlines of the cytoplasm and the nucleus. Color around each cell correspond to the color code of the plots in c). **b)** Plot of the values of a feature for different simulations of cells with random mRNA distribution. Each value is colored according to the cell shape **c)** Ratio of the variance between and within cells for the two normalizations. The colored dashed lines indicate the example shown in b).*

3.2. FEATURES DERIVED FROM RIPLEY-K FUNCTION

The Ripley-K function allows quantifying the level of aggregation or dispersion of a spatial point cloud by comparing local densities to the expected local densities under the assumption of complete spatial randomness (CSR). It can be computed for a certain distance range *r*:

$$K(r) = \frac{1}{n}\sum_{i=1}^{n} N_{p_i}(r)/\lambda \quad \textbf{(1)}$$

where n is the total number of RNAs in the cell, $N_{p_i}(r)$ the number of mRNA present in a circle of radius r around the ith mRNA and λ the total density of mRNA in the cell. Please note that these calculations are performed in 2D, i.e. after projection of the data into the xy-plane. The K-function can be normalized such that it gives 0 for CSR (See Fig S21b). We refer to this function as L-function:

$$L(r) = \sqrt{\frac{K(r)}{\pi}} - r \quad \textbf{(2)}$$

For a given value of r, values > 0 indicate spatial clustering, while values < 0 indicate spatial dispersion.

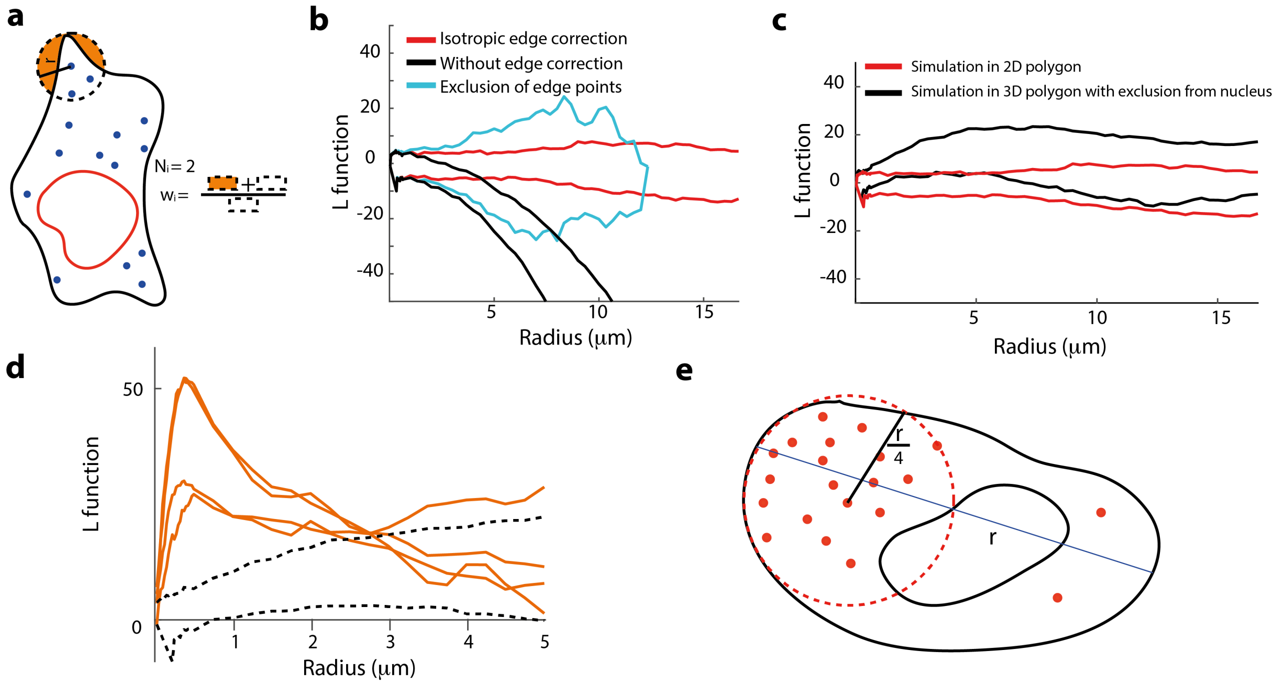

Supplementary Figure 21. Ripley-K function and mRNA localization. a) *Illustration of the computation of the Ripley-K (or L) function with isotropic correction.* ***b)*** *Result of the Ripley-K function for complete spatial randomness. mRNAs are simulated in 2D and confined to an experimental cellular outline. L-function was calculated for different conditions: (black line) with no edge correction, (blue line) after exclusion of mRNAs at the edge, (red line) after isotropic correction. Shown are 5% and 95% percentiles.* ***c)*** *Impact of 3D cellular environment. Red curve from b). Black curve is L-function from mRNAs simulated in a 3D cellular outline, with exclusion from the nucleus. L-function is computed with isotropic edge correction on xy-projected data.* ***d)*** *Comparison of L-function for 3D data of randomly located mRNAs (dashed data), or mRNAs showing aggregation.* ***e)*** *Cartoon illustrating how the L-function is influenced at larger radii when mRNAs show polarized localization.*

One major limitation in this definition is the impact of borders. For points close to the border, the allowed area around a point *i* is typically smaller than in the interior of the region. This can then lead to a systematic underestimation of the number of neighbors, and an artificially low L-function (Fig S21b, black line). We compared two different methods to reduce this bias. First, spots close to the border can simply not be considered in the analysis. While this approach reduced the bias in the L-function, it leads to large increases in the variance of the estimation (Fig S21b, compare blue and black curve), as the estimation is

based on fewer samples. Second, we can compute an isotropic correction factor w_i. This factor is defined as the ratio of the area of the intersection of a circle with radius r around each point p_i with the cellular regions S and the area of the circle (Fig 1a). This factor will be 1 for mRNAs with a distance of at least r to the cell boundary, and >1 for mRNA that are closer than r to the border

$$w_i = \frac{\#\{u_j \mid u_j \in S \wedge \|u_j - p_i\| \leq r\}}{\pi r^2}$$

$$K'(r) = \frac{1}{n}\sum_{i=1}^{n} w_i N_{p_i}(r)/\lambda \quad \textbf{(3)}$$

From this definition, we can calculate the corresponding L' function as defined above. We speculated that this function, evaluated for different values of r, may provide a signature to identify the occurrence of mRNA foci. We therefore calculated the L-function profiles for simulated cells with either random mRNA localization, or mRNA foci (Fig S21d). Cells with foci show a distinct peak of the L-function for small values of r. We summarized this behavior with a number of simple features, where the parameter D is set to be larger than the size of the observed aggregation structure (here we set D to 4µm).

1. **Maximum value of the L-function** in the [0;D] interval:

$$max_{\mathrm{L}} = \max_{r<D} L(r)$$

2. **Maximum value of the gradient of L-function** in [0;argmax$_{r<D}$(L)]:

$$max_{\mathrm{grad(L)}} = \max_{r<argmax(L)} grad(L(r))$$

   The Ripley function is first smoothed with moving average of 4 pixels before gradient computation.

3. **Minimum value of the gradient of L** in [argmax(L);D]:

$$min_{\mathrm{grad(L)}} = \min_{argmax(L)<r<D} grad(L(r))$$

   The Ripley function is first smoothed with a moving average of 4 pixel computing the gradient.

4. **Monotony of the L-function in the [0;D] interval**: described with Spearman correlation L_{corr} between the L-function and the distance r.

We next speculated that the L-function may provide also information about mRNA aggregation on a larger scale, for instance for polarized cells. A polarized cell has by definition a non-uniform mRNA density, but the non-uniformity is observed at a much larger scale than it is the case for foci patterns. If a cell with a length L is polarized so that only one half of it contains mRNA, the effect on the Ripley function will be most visible at a scale around $L/4$ length (Fig S21e). We therefore designed a simple feature capturing this case

5. **L-function at $L/4$,** where the length of the cell L is calculated as the maximum distance between two points on the polygon defining the cellular border.

3.3. FEATURES DESCRIBING LOCALIZATION AT THE CELLULAR MEMBRANE IN 3D

In one of the simulated mRNA localization patterns, we placed mRNAs close to the cellular membrane in 3D. This localization pattern is challenging to detect since in typical experimental data, only information

about the 2D outline of the cell (e.g. by staining with CellMask^{TM}) is available. This is also due to the fact that the preferred image acquisition mode for smFISH data is wide-field microscopy, making the 3D segmentation of the cell membrane very complicated and error-prone even if a membrane marker is used. So, we can generally assume that in most smFISH data sets, information on the cell membrane in 3D is normally not available. Therefore, it is not possible to compute a 3D distance between mRNAs and the cell membrane, which would be the most direct feature to detect this localization pattern. Further, this localization pattern is also difficult to detect by manual inspection of the data (which is often performed in maximum intensity projection along z), since the information about the z-position is lost.

We therefore aimed at implementing a feature which can be readily computed from typical smFISH data and allows inferring if mRNAs are localized towards the cellular membrane in 3D. We based this feature on the observation that after z-projection (maximum, or median), the unspecific FISH signal appears brighter in the center of the cell than at the periphery of the cell, which suggests that there is some correlation between the intensity of the FISH background signal and the cell height. This can be explained by the presence of FISH probes inside the cell, whose number should correlate with the available volume, and thus the local height of the cell. We therefore hypothesized that we can use the intensity of the background smFISH signal to estimate the height of the cell.

We therefore wanted to experimentally test this hypothesis. Owing to the way in which we generated the virtual cell environment for simulation (see Note 1d), we can rely on both smFISH background signal, as measured from mockFISH experiments and precise measurements of the actual cell height, as measured from the localization of GAPDH mRNA. In real data, we do not have direct access to the smFISH background, but only to the entire smFISH data, containing both background and signal. We therefore simulated cells with randomly localized mRNAs (Fig S22a).

The first problem is to remove the mRNA signal in order to obtain some approximation of the smFISH background. This can be achieved by a series of filtering operations. First, we applied a morphological opening to the maximum projection of the image stack to remove the signal of the mRNAs. We fixed the size of the structural element to 10 pixels, enough to remove even larger mRNA foci. Second, we smoothed the image with a Gaussian Kernel (7*7 pixels) (Fig S22b-c). We then asked if this processed smFISH background image could be used as an approximation of the actual cell height (Fig S22d). We found that height and smFISH background intensity were highly correlated ($r^2 = 0.96$), illustrating that the non-specific FISH signal can be used a proxy for cell height. This strategy is particularly interesting, as it does not require any change in the experimental setup nor an additional marker: the 3D information is present in virtually all smFISH screens. The underlying hypothesis is that it is possible to remove RNAs and to distinguish them from the background, which might be more challenging for genes with very high expression level.

We next asked if the approximated cell height can be used to determine localization of mRNAs at the cellular membrane. To obtain a qualitative impression, we compared a cell with random localization (Fig S22f) to the same cell with localization towards the membrane (Fig S22g). For visualization, we renormalized the image approximating the cell height to values between 0 and 1. We then plotted the estimated z-position against the approximated cell height. When comparing the plots for these two localization patterns (Fig S22f and S22g), it appears that the cells with a membrane localization shows more correlation between these two measurements. We therefore calculated two features to exploit this observation

1. $Corr_{\text{zcell}} = corr(z_i, \text{BG}(x_i, y_i))$, where BG*(x,y)* is the background approximation obtained as described above at point *(x,y)*.

2. $r^2_{zcell} = R^2$, i.e. the coefficient of determination, where *BG(x,y)* is the predictor and *z* the predicted variable.

When calculating these two features for 100 cells, we found that indeed the two populations are well separated (Fig S22h).

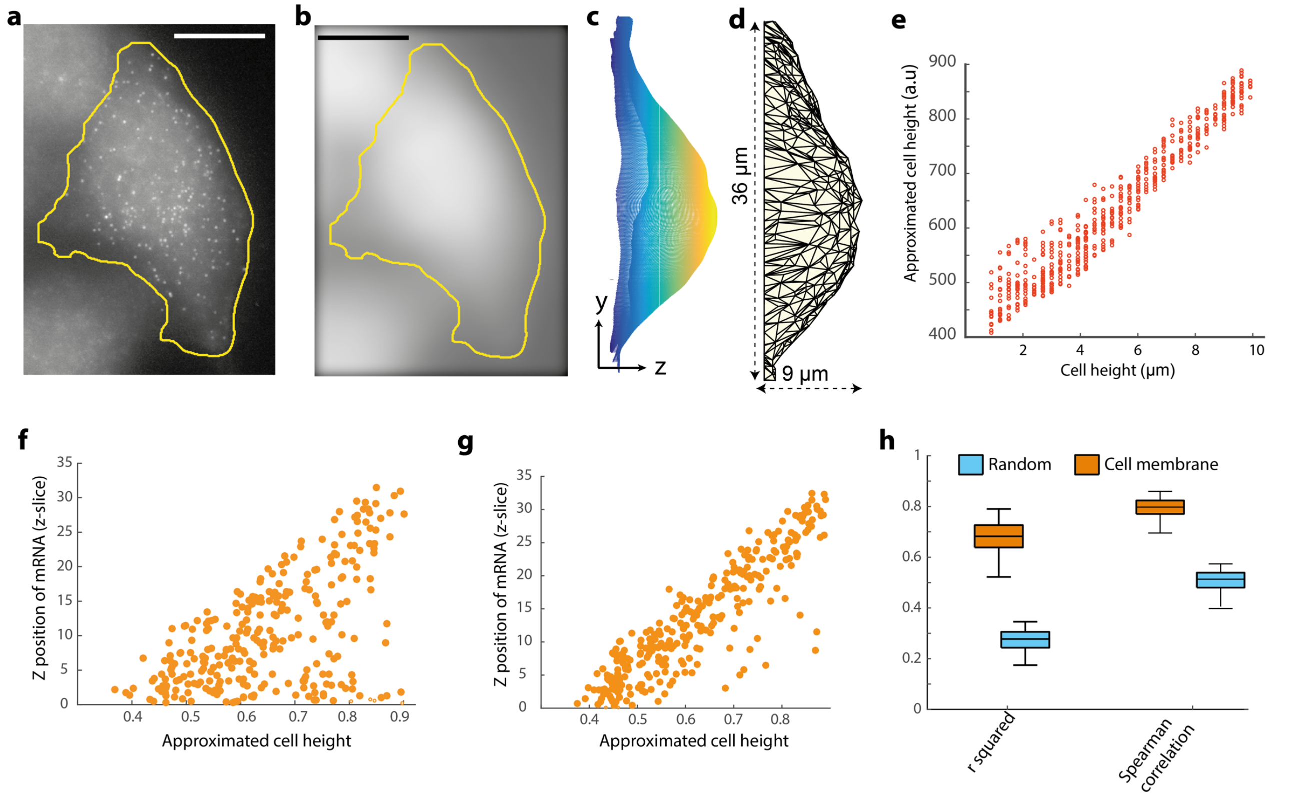

Supplementary Figure 22. a) *Simulated image with random mRNA localization. Image is 3D but shown as a maximum intensity projection along z. Yellow line shows cell outline.* ***b)*** *Image after morphological opening and Gaussian filtering.* ***c)*** *3D polygon of the cell resulting from the detection of the GAPDH mRNA as detailed in Note 1. Shown is a yz -side-view.* ***d)*** *Relative height approximation of the cell. Plot shows the intensity value of b) plot as a surface plot and displayed along the yx-plane.* ***e)*** *Plot of cell height approximation as a function of the actual cell height.* ***f)*** *Plot of the z-position of the detected mRNA (randomly localized) in a) against the approximated cell height (intensity values of b)* ***g)*** *As in f), but for a cell with mRNAs localized towards the cellular membrane.* ***h)*** *Distribution of the Spearman correlation and the r^2 for cells with random localization (blue) versus cell with localization towards the membrane (orange). Scale bars 10 µm.*

3.4. FEATURES BASED ON MORPHOLOGICAL OPENING

We found that some mRNAs are specifically enriched in cellular extensions. None of the features we mentioned so far specifically captures this localization pattern, as it is intimately related to morphological properties of the cell. We therefore reasoned that informative features could be retrieved by partitioning the image region according to morphological criteria in line with the definition of cellular extensions. Informative features can then be defined from the resulting spatial histograms (i.e. the relative percentages of mRNA in each of the regions). Here, we applied successive morphological openings to the 2D mask of the cell (Fig S23). Each of those opening separated the cell in two parts corresponding to the pixels that belong interior of the cell and the parts that were cut off. For each opening step ai, we compute the ratio of number of mRNAs in the part of the cell that was removed to the total number of mRNAs.

$$spot\_ratio_{\mathrm{ai}} = \frac{\#\{spot\ opening_{\mathrm{ai}}\}}{\#\{spot\}},$$

The useful sizes of these openings depend on both the morphological properties of the cell line used and the spatial resolution of the microscope. In order to be more generally applicable, we can calculate these ratios for a large series of values. For our cells, we found that openings of 30 and 45 pixels were most discriminative between random localization and localization in extensions, and so we kept only these two values.

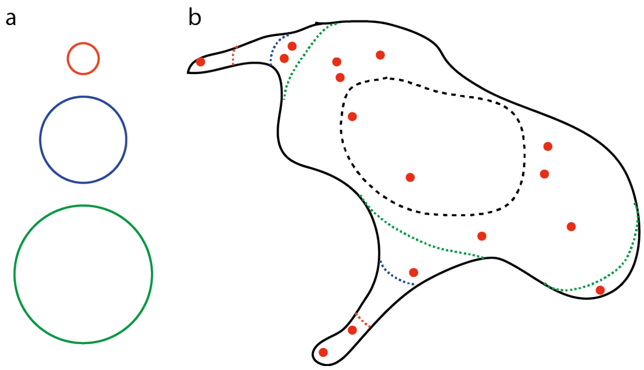

***Supplementary Figure 23**. **a)** Structural element of different size indicated with circles of different colors. **b)** Cartoon to illustrate the results of a morphological opening with the structural elements from a). Dashed lines indicate which part of the cell will be cut with each of the structural elements.*

Supplementary Note 4: Analysis of mRNA localization in simulated images

To validate the methodological framework to analyze the spatial distribution of single RNA molecules, we analyzed the simulated data described in Note 1. We simulated 8 different localization patterns and a total of 8800 cells representative for a large range of parameter combinations, namely the strength of the pattern and the number of RNAs.

4.1. OVERVIEW OF ANALYSIS METHODS

In order to visualize these high-dimensional feature sets, we performed a t-SNE projection[20] into a 2D space. This allows a qualitative inspection of how well cells with different localization patterns are separated and points to patterns for which distinction is still challenging. We also performed k-means clustering on the full feature space with the number of classes corresponding to the number of simulated localization patterns. Inference of the number of classes in an unsupervised learning problem is often a challenge by itself; we will specifically address this point later for both simulated and experimental data. In a first analysis however, we want to test whether the set of features do in principle allow us to separate the known patterns by an unsupervised learning method. We therefore perform a first series of tests in the favorable case where the number of classes is known a priori. A convenient way to show the results of such a classification is a confusion matrix. The automatically identified classes correspond to rows, and the ground truth patterns correspond to columns. Each number (i,j) of the matrix corresponds to the percentage of cells simulated with pattern i placed in class j by the clustering algorithm, where the percentage is calculated with respect to the true number of cells in each class. As there is no clear rule of which automatically defined cluster corresponds to which a priori class, we arrange clusters in such a way that the maximal value falls on the diagonal of the confusion matrix. Hence, for a perfect classification, only the diagonal is populated. Off-diagonal values are misclassifications. We further calculate the rand index, which quantifies the level of agreement between two clustering results. It computes among all the pairs of cells, the proportion of pairs for which the two classifications agree. Agreement is obtained either if in both classifications (the automatic classification and the ground truth), the cells are attributed to the same cluster, or if in both classifications both cells are attributed to different clusters. A value of 1 for the rand index means that the two classifications agree on all the pairs of cells. Here, we calculated the rand index between the obtained k-means results and the known ground truth, e.g. where cells with the same localization pattern are in the same cluster.

4.2. ANALYSIS BASED ON PUBLISHED MRNA DETECTION AND LOCALIZATION FEATURES

We implemented a framework based on the seminal paper of Battich et al[5]. In short, we performed mRNA detection in 2D (after a maximum intensity projection along z) with a deblending algorithm[5] to separate close mRNAs. From these detection results, we calculated the proposed 32 localization features, which can be summarized in several groups:

- **Distance calculations between mRNAs.**
  - For each mRNA, the mean and standard deviation of distances to all other mRNAs is calculated. A given cell is then described by the mean and standard deviation of these distances for all mRNAs in this cell.
  - For each mRNA, the ratio of spots contained in a neighborhood of a fixed radius r (for different values of r) are calculated. The mean and standard deviations for each considered radius are used as a feature.

- For each mRNA, the radius of a circle centered in this mRNA is determined such that a defined fraction of mRNAs falls into the circle. This gives a distribution of radii, which can be represented by mean and standard deviation. These two features (mean and standard deviation) are calculated for different fractions.
- Mean and standard deviation of the distances between mRNAs and cellular compartments (centroid of the nucleus or cell, and cellular membrane).

Classification of cells with constant mRNA density

We first analyzed cells with one mRNA density (average of 200 mRNAs per cell). The t-SNE analysis shows that among the eight simulated localization patterns, four could be readily distinguished: cell membrane 2D, nuclear envelope 2D, nuclear envelope 3D, and polarized (Fig S24a).

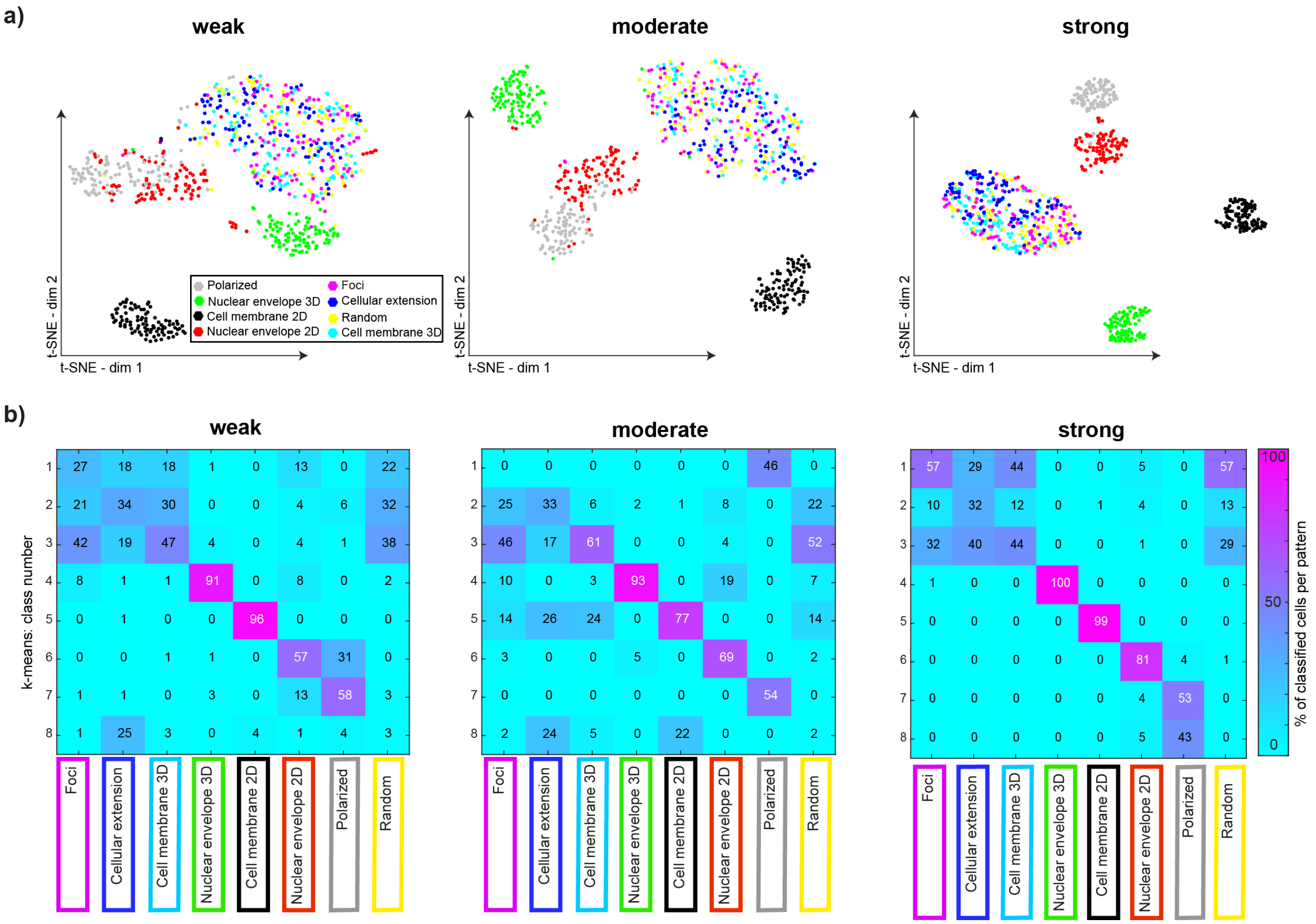

Supplementary Figure 24. **a)** *t-SNE projection of the localization features for simulated smFISH images with different mRNA localization patterns. Each dot corresponds to one cell and is colored according to its mRNA localization pattern. Three different pattern strengths were simulated and analyzed separately.* ***b)*** *Confusion matrix showing the results of the k-means classification with 8 classes of the data in a). The classification was performed in the original feature space.*

As expected, increased pattern strength leads to a clearer distinction. The remaining four patterns (mRNA foci, cellular extension, cell membrane 3D, random) are in the same cluster and cannot be separated. The k-means classification shows similar results (Fig S24b), with the same four localization patterns that cannot be distinguished in the t-SNE being grouped together. Additionally, it also shows how with weaker pattern strength, some localization patterns that are close in the t-SNE (nuclear envelope 2D and polarized)

are also frequently confused. This first analysis shows that additional localization features are needed to better separate all simulated localization patterns. We would like to note that these results do not question the results obtained in the original paper[5] but suggests that potentially more localization classes could be extracted with an improved analysis.

4.3. IMPACT OF MRNA DETECTION IN 3D AND GMM ANALYSIS OF MRNA FOCI

Before developing new features, we investigated the impact of an alternative mRNA detection method that could provide more information and hence had the potential to improve the descriptive power of the localization features. In the previous analysis, detection was performed in 2D with a deblending algorithm. An analysis in 2D speeds up computation time and reduces storage space, but information about the 3D localization of mRNAs is lost. Further, the used deblending algorithm helps to separate close mRNAs but was not developed to decompose very densely packed mRNA foci. We therefore analyzed the same data with a 3D mRNA detection approach combined with the GMM analysis (Supplementary Note 2) and calculated the same features as in Fig S24.

The most obvious difference in this new analysis (Fig S25) is that cells with mRNA foci (pink dots in the t-SNE) start to be better separated for strong patterns. However, they remain partly mixed with other localization patterns, and especially for weak and moderate pattern strength they are not in a separate class. Nuclear envelope 2D and polarized localization are also somewhat better separated but remain frequently confused for weaker pattern strength. Lastly, three patterns (random, cellular extension, and cellular membrane) remain within the same cluster.

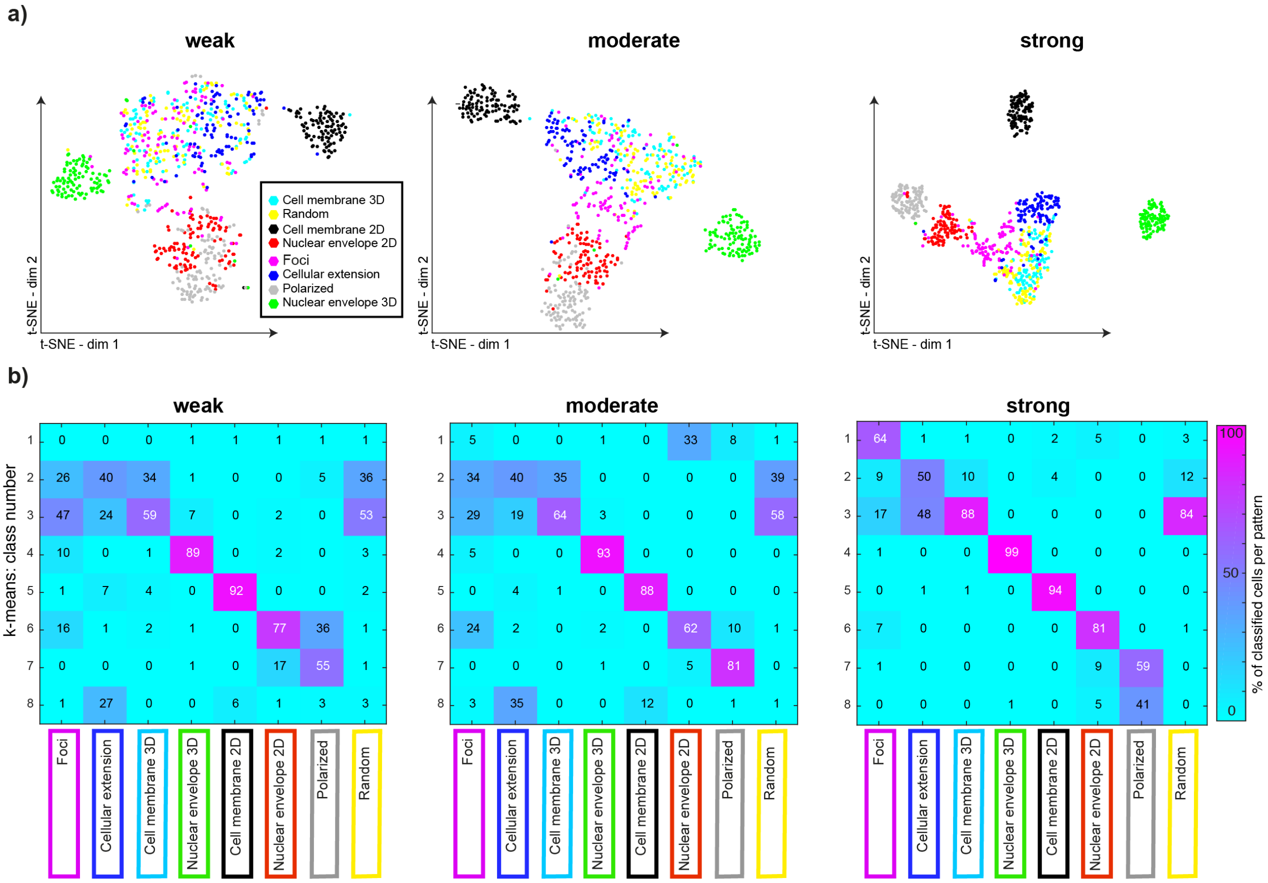

Supplementary Figure 25. *t-SNE and confusion matrix calculated and displayed for the feature set used in Fig S24, but based on a mRNA detection in 3D with a GMM analysis to decompose mRNA foci.* **a)** *t-SNE projection of the localization features for simulated smFISH images*

with different mRNA localization patterns. Each dot corresponds to one cell and is colored according to its mRNA localization pattern. Three different pattern strengths were simulated and analyzed separately. ***b)*** *Confusion matrix showing the results of the k-means classification with 8 classes of the data in a). The classification was performed in the original feature space.*

4.4. NEW SET OF LOCALIZATION FEATURES

The analysis shown in section 4.3 revealed that our newly developed 3D detection including a powerful decomposition scheme did not increase the overall accuracy dramatically. Indeed, even though both 3D information and decomposition of tight RNA clusters are essential to describe the properties of some of the patterns, proper detection alone is not enough to lead to a real increase in performance. The relevant properties of the underlying spatial distributions need to be represented by new features, which we have newly introduced and described in detail in Supplementary Note 3. In short, we calculate features

- … describing mean distances between mRNAs and cellular compartments using the CSR normalization.
- … based on Ripley's L-function, which measures spatial homogeneity on different length scales.
- … using morphological image processing to obtain localization in cellular extensions.
- … relevant for the positions with respect to the cell membrane in 3D using an approximation of the cellular height.
- … calculating the mean percentage of number of mRNA molecules in the nucleus.
- … described in a recent paper[21] which capture polarized mRNA localization by calculating a dispersion and polarization index.

Finally, we removed the features calculated as the standard deviation of the various distances (both inter-point and distance to cellular compartments). As the purpose of this study is to study mRNA localization patterns, it is desirable that features are independent from mRNA abundance. In the contrary case, the final localization patterns might not represent different localizations, but different expression levels. However, in practice such a dependence cannot be excluded if the number of RNAs is particularly low. In this case, the estimation of each of the mentioned features relies on few sample points and can thus lead to a different result at the single cell level. But, we can at minima require that features should be defined in such a way that on average they are independent from the number of RNAs. For instance, the average distance between points is obviously dependent on the number of points in the volume/area of interest and should therefore not be used as a feature. On the other hand, the percentage of points falling into a circle of radius r around each point, averaged over all points, does not depend on the number of points – on average (over all cells). However, the variance of the percentage of points falling into these circles, does depend on the number of points: it is systematically larger for fewer points; this is also true if we average over many cells. Here, the dependence is not a result of the uncertainty of estimation, but it is intrinsically related to the definition of the descriptor.

We calculated these features for the 3D spot detection with GMM used before. The t-SNE projection showed very distinct clusters for each of the 8 localization patterns for the two moderate and strong localization patterns (Fig S26a). The k-means clustering is also dramatically improved with strong diagonals for moderate and high pattern strength, indicating low misclassification rates.

Analysis of pooled pattern strengths

Next, we pooled all pattern strengths (Fig S26b). In the t-SNE visualization, most cells are grouped together based on their localization pattern. However, some localization patterns are less well separated. This is also further confirmed by k-means clustering, where some misclassifications occur, most notably for cell membrane 3D and random localization, which are in the same class. Interestingly, these two

classes are, however, largely not overlapping in the t-SNE space, which indicates that this is rather a problem of clustering than that of feature representation. It must be noted, that the weak patterns really represent many borderline cases, where even manual annotation would be far from accurate. Nevertheless, we felt that improvement on clustering the weak patterns would still be possible by testing other clustering methods.

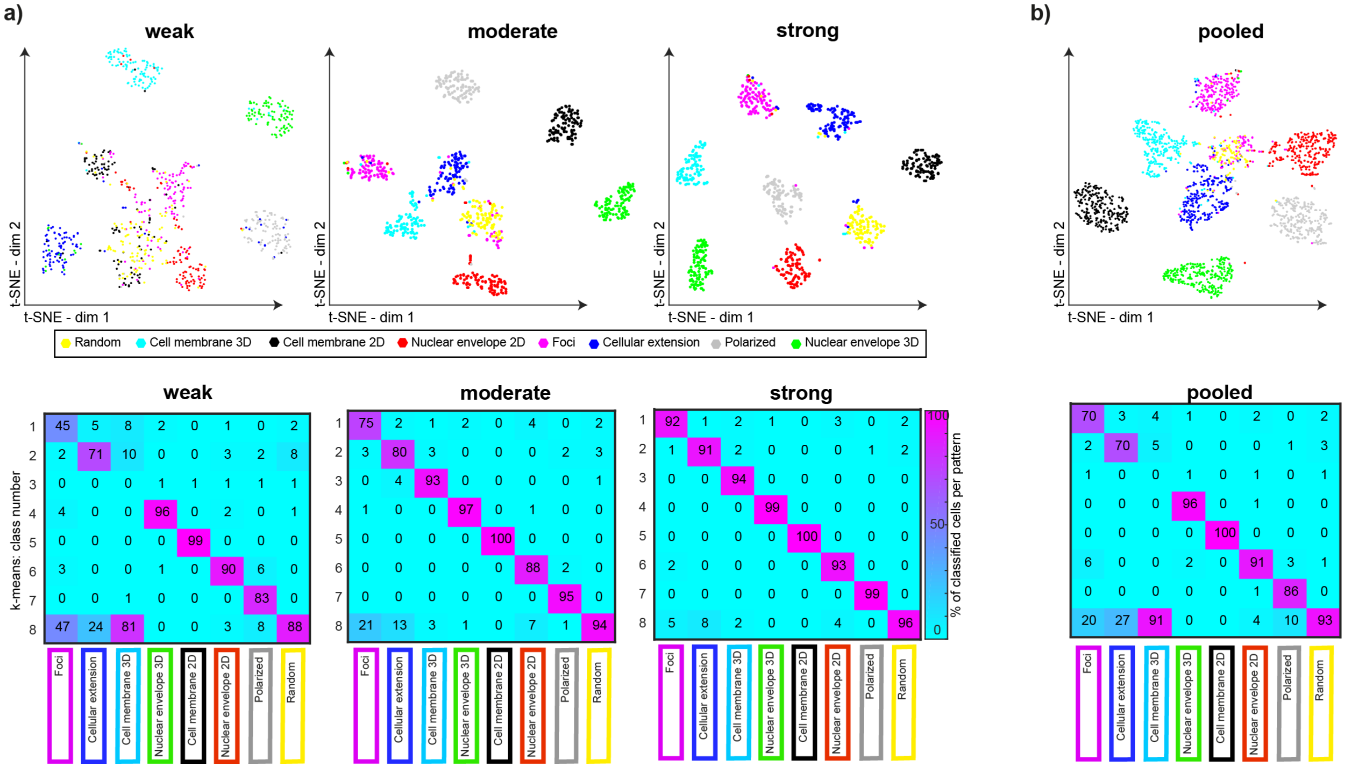

__Supplementary Figure 26.__ t-SNE projection and confusion matrix for analysis with new features. __a)__ Separate analysis of the different pattern strength. __b)__ Analysis results for pooled pattern strengths.

Analysis of pooled patterns strengths and expression levels

All preceding analysis was done with only one expression level. In practice however, expression levels vary between different cells. One important requirement of the mRNA localization analysis is therefore robustness to changes in expression levels of different genes and between different cells. Different genes should be grouped together because they are similar in their mRNA localization and not because they have similar expression levels. We therefore analyzed the entire simulated cell population (with pooled expression levels and pattern strengths).

The t-SNE projection reveals that cells are – as desired – largely grouped by their localization pattern and not their expression levels (Fig S27a), even if these groups are not as clearly separated as in the analysis before. The increased heterogeneity also results in more misclassifications by the k-means clustering (Fig S27b-c). This indicates that the classification results for some patterns can be affected by heterogeneity in expression level and pattern strength. However, we also observed that despite this increased misclassification rate, most cells with a given localization pattern are close in t-SNE space. This has two important implications. First, a visual inspection of the t-SNE projection can be informative before

clustering is performed. Second, we speculate that a pre-processing of the feature space with t-SNE could be beneficial for the clustering accuracy.

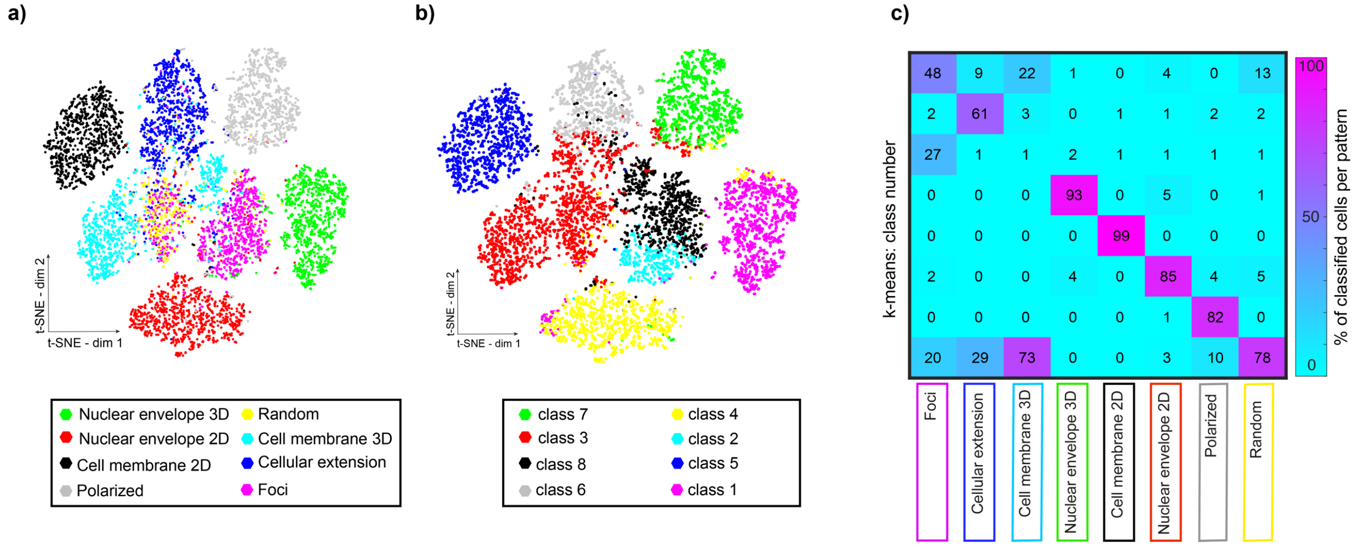

Supplementary Figure 27. *t-SNE projection and confusion matrix for analysis with new feature set.* ***a)*** *t-SNE projection of a cell population with 4 different mRNA densities and three different pattern strengths. Each point is a cell colored according to its localization pattern.* ***b)*** *t-SNE projection of a) colored according to k-means classification results.* ***c)*** *Confusion matrix resulting of the k-means classification on the data shown in b).*

Analysis of very high expression levels

We lastly investigated how robust the analysis is towards ever higher mRNA levels. Analysis of highly expressed genes is in general challenging for smFISH, because the high local densities makes individual mRNAs difficult to separate. But such high densities will also impact some of the localization features, most notably Ripley's L-function which measures spatial clustering and dispersion. To more rigorously test this, we performed additional simulations with high an expression level (on average 800 mRNAs per cell, but with some cells having up to 1500-2000 mRNAs , Fig S28a). We pooled these simulations with a second data set with an average of 200 mRNAs per cell. The confusion matrix for this pooled data-set reveals that correct clustering was achieved for almost all localization patterns, despite the wide range of expression levels (Fig S28b). However, and as speculated, foci were commonly misclassified as random localizations. Closer inspection of these results revealed that: (i) the mis-classified cells were those with high expression levels, (ii) they were mixed with random since the characteristic foci-signature of the Ripley-features disappeared. These simulations show that the expression level indeed impacts the detection accuracy of some localization patterns, but also that most patterns are not affected.

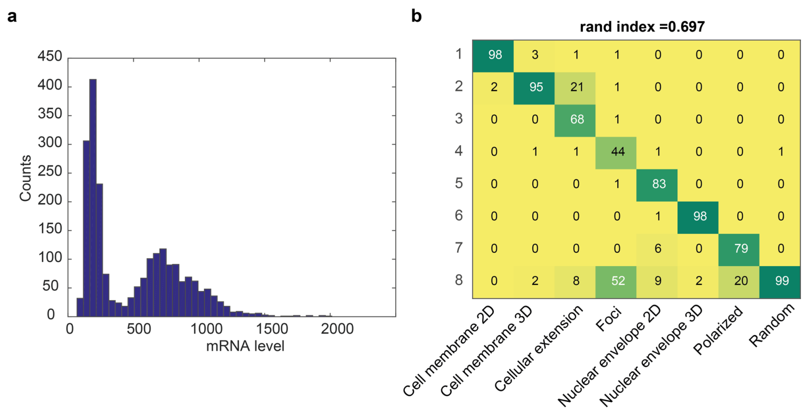

***Supplementary Figure 28**. **a** Histogram of mRNA levels after pooling simulations with an average of 200 and 800 mRNAs per cell. **b** Confusion matrix of analysis of pooled cell population.*

4.5. TEST OF DIFFERENT CLASSIFICATION METHODS AND PRE-PROCESSING

In order to test different classification methods, we analyzed all pattern strengths and expression levels separately and also different pooled combinations. For each analysis, we performed k-means classification and computed the rand index as a measure of the quality of the classification (Fig S29a and Fig S30a). We then used those results as a reference to judge possible improvement of the classification accuracy. We first tested other non-supervised classification methods (spectral clustering and Gaussian mixture model) and did not find an overall improvement compared to the k-means clustering (Fig S29b-c).

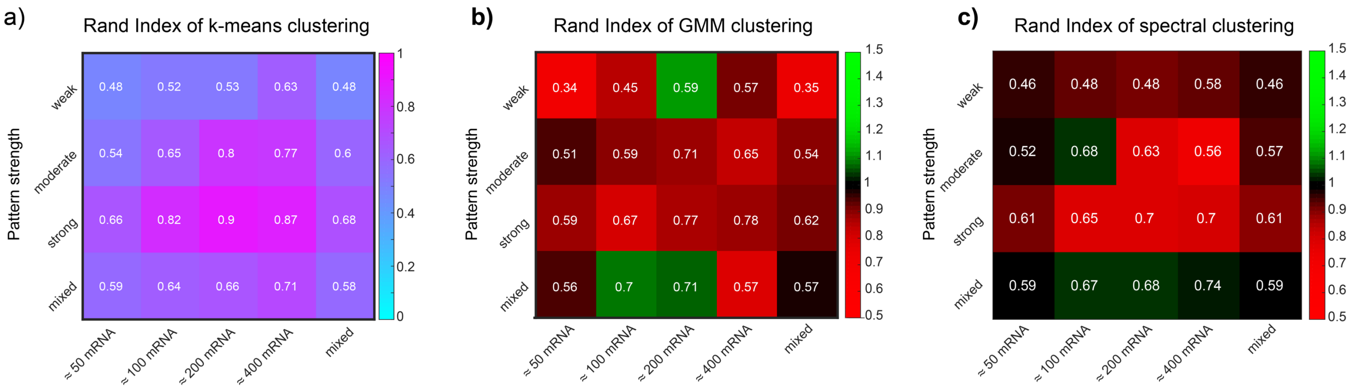

***Supplementary Figure 29**. **a)** Results of the k-means classification with 8 classes for different combinations of mRNA densities and pattern strengths. The rand index is displayed as a measure for the classification quality, and the matrix is colored according to its value. **b)** Results of GMM clustering for different combinations of mRNA densities and pattern strengths. **c)** Results of spectral clustering for different combinations of mRNA densities and pattern strengths.*

We next pre-processed the feature space either with a principle components analysis (PCA) or t-SNE. We then applied k-means clustering to these pre-processed data. For PCA, we found that only moderate improvements can be obtained even if an increasing number of dimensions are used (Fig S30b). In contrast, we found that by pre-processing with t-SNE, the classification results improved substantially (Fig S30c). By combining pre-processing with t-SNE and k-means clustering, we could obtain almost perfect classification – even when pooling cells with vastly different expression levels and different localization pattern strengths (Fig S30d).

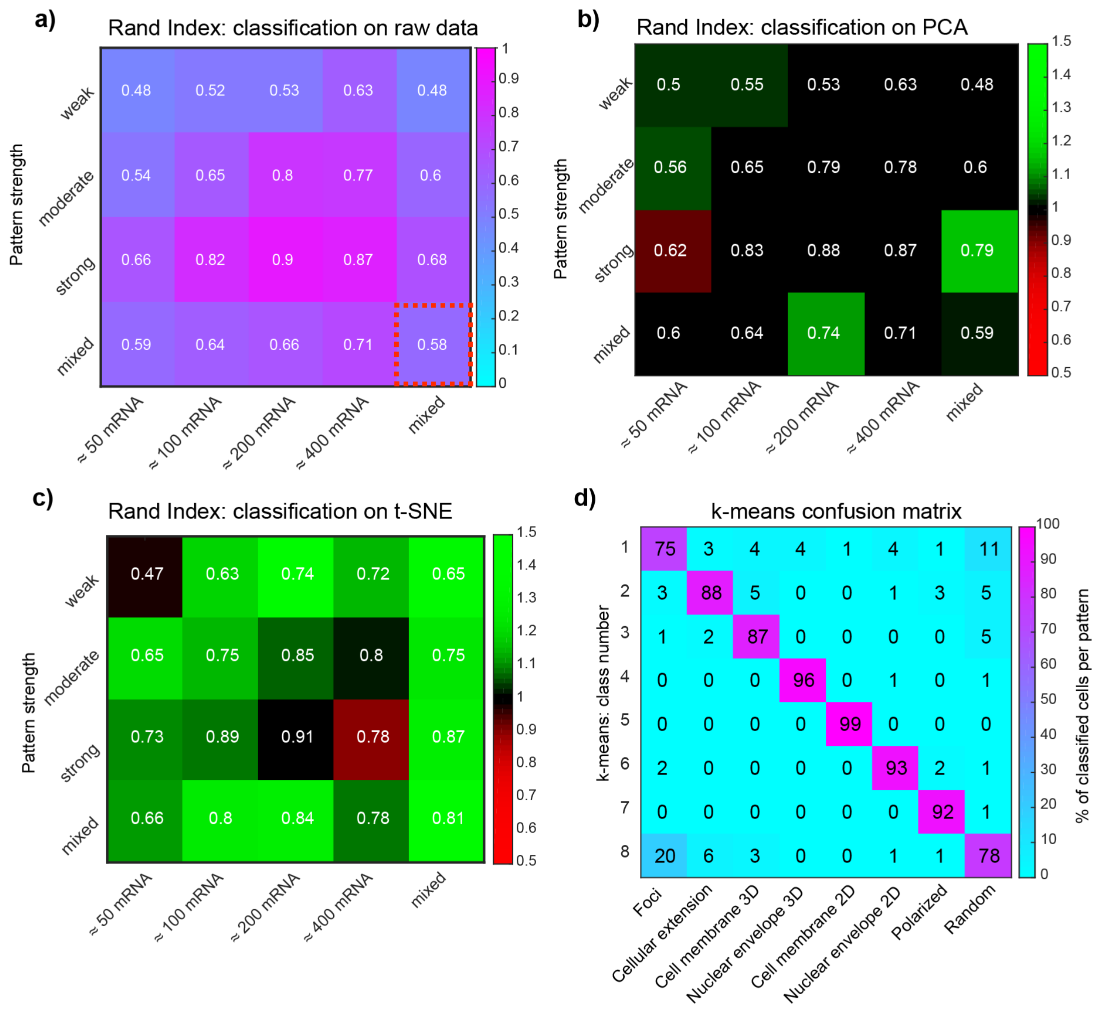

***Supplementary Figure 30**. **a)** Results of the k-means classification with 8 classes for different combinations of mRNA densities and pattern strengths. The rand index is displayed as a measure for the classification quality, and the matrix is colored according to its value. **b)** Results of the k-means classification with 8 classes for different combinations of mRNA densities and pattern strengths. The classification is performed on the 6 first components of a PCA. The rand index of this classification is shown as a value in the matrix while the matrix is colored according to the ratio between the rand index displayed and the results of the classification on the raw data shown in a). Ratio values above 1 are displayed in green indicating an improvement over the results in a, while values smaller than 1 are displayed in red and indicate a deterioration of the classification. **c)** Same as b) with a classification performed on a t-SNE projection on 6 components.). d) confusion matrix corresponding to the analysis of the pooled cell population (all pattern strengths and expression levels) after pre-processing with t-SNE.*

4.6. AUTOMATED DETERMINATION OF THE NUMBER OF CLASSES

One important question in unsupervised clustering is the determination of the number of clusters. In the preceding analysis, we used the known number of localization patterns as the number of classes in order to carefully validate the localization features. However, in the analysis of experimental data, this information is usually not available. There are several methods that can provide an indication on the number of classes and we tested two methods on our simulations: the silhouette score and the within-class-variability.

The silhouette score computes a score for each data point (here, a data point corresponds to the feature vector of one individual cell) the difference between the average distance of the data point to the

points of its own cluster and the average distance of the data point to the points of the closest among all other clusters, normalized to the maximum of these two distances.

The value of this score, calculated for each data point, is between -1 and 1. A score close to 1 means that the dissimilarity of the data point to the other members of its own cluster is much smaller than the dissimilarity of the data point to the members of the closest of all other clusters. A value of 1 therefore means that the clustering decision for this data point was sensible. For a score of 0, the average dissimilarity with the assigned cluster is equal to the average dissimilarity to the closest among the other clusters, which means that the point lies between two clusters, and attribution to one of them might be difficult. A score of -1 means that the clustering decision was badly chosen, as there is a cluster with much lower average dissimilarity to the data point than the one that was chosen.

For a clustering result for the entire data set, the scores of all data points are averaged to get a single score. By computing the score for different numbers of classes, the most suitable number of classes is the one with the highest score.

Another way to have an indication for the number of classes is to compute the within-class-variability. This metric is the weighted sum of the variability inside each cluster. This value decreases with increasing number of classes. A good indication for the suitable number of classes – according to the elbow method – can be found by analyzing the curve that describes the within-class-variability as a function of the number of classes. A suitable number of classes corresponds to the kink of the curve, i.e. the point beyond which the within-class-variability does not decrease very much anymore.

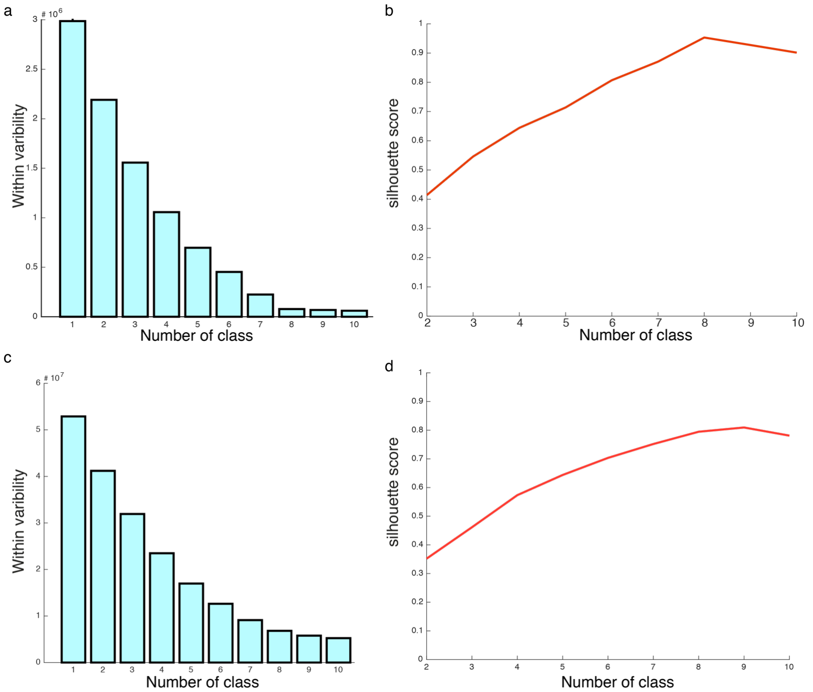

Supplementary Figure 31. Determining the number of classes for un-supervised classification. a) *Within-class-variability plotted for a cell population with pooled mRNA densities and pattern strength (data from Fig S28)* ***b)*** *Silhouette score plotted for the same cell population as a).* ***c-d)*** *As in a-b, but for data with only strong localization pattern and high expression level. Correct number of classes is 8 in either case.*

We first analyzed data where the best clustering results were obtained, i.e. with cells showing only strong localization patterns and large mRNA density (see Fig S30a for the highest rand index). Both, the within-class-variability and the silhouette score correctly yielded 8 classes (Fig S31a-b). When we analyzed the pooled simulated data, the results were more ambiguous. For either method, no clear recommendation for using 7, 8 or 9 classes was obtained (Fig S31c-d). This illustrates that with increasing heterogeneity in the data, determining the underlying number of classes is more challenging. But we also see that we get an estimation, which is reasonably close to the true number of classes, for both criteria.

4.7. PATTERN STRENGTH AND DETECTION ACCURACY

We can use our simulation framework to analyze the impact of the pattern strength on the outcome of the analysis. In particular for drug experiments, it might be interesting to get an intuition which pattern differences are distinguishable with the proposed framework. In order to illustrate this, we simulated 3 non-random patterns in varying strength:

- Nuclear envelope 2D (1000 samples, 10 different pattern strengths)
- Cell membrane 2D (996 samples, 10 different pattern strengths)
- mRNA foci (1997 samples, 20 different pattern strengths)

The pattern strength controls how many mRNAs contribute to the pattern. The other mRNAs are placed randomly in the cytoplasm. In addition, we simulated 100 random patterns.

We then generated t-SNE and PCA projection of the data, where we color coded the pattern strength (Fig S32a-b). In both representations, we see that the strongest patterns localize far away from the random patterns. We also observe that for the weakest patterns, there is some overlap with random localization (Fig S32b). In agreement with our previous analyses, we observe that t-SNE leads to better cluster separation, but also moves some cells with random localization (< 5%) towards regions dominated by non-random localization. Overall, the results are similar between the two projection methods. We then asked how many cells with non-random localization would have been correctly identified as being non-random. For this, we determined the centroid of the random population, and the 90%, 95% and 100% percentiles of the distribution in projection space. As expected, we find that the percentage of cells with distance larger than these thresholds depends on localization pattern and pattern strength (Fig S32c): it is lowest for the weakest patterns, indicating large overlap with the random localization class and increases with pattern strength reaching 100% for stronger patterns. The overlap is class dependent: we see that for the localization at the cell membrane, the curves rapidly reach 100% and even for the weakest pattern strength simulated, a fraction of cells is different from the controls (depending on the percentile threshold used). We believe that this is due to the fact that statistically, it is highly unlikely that mRNAs localize in close proximity to the cell membrane and consequently, the feature signatures indicate this pattern even if only a small fraction of mRNAs localizes at the cell membrane. For foci in contrast, we observe that – while strong patterns reach also 100% - the curve is less steep and moderately weak patterns still tend to overlap with random localization. We assume that foci can appear easier for purely statistical reasons, and the measured distances in feature space are therefore smaller for weak and moderate patterns. For each pattern, we selected some points in the curve (orange lines in Fig S32c). We show examples for these points (two cells per pattern, Fig S33). This gives an impression on the pattern differences that can still result in a measurable signal.

We next turned to supervised analysis, where we first generate simulated ground truth data and then learn from the samples with known ground truth. In order to analyze the effect of pattern strength on the accuracy of trained classifiers, we considered binary classification between random and each of the non-random localizations in turn (Random Forests: 300 trees, a maximal depth of 20). In a first setting, we considered the case where we train a classifier on extreme cases (strongest pattern). This mimics a popular strategy for manual annotation, where we would annotate only those cells for which we are very confident. It also covers another strategy of ground truth generation, where we generate extreme patterns experimentally, for instance by using a high dose of a drug. In our in-silico experiment, we learn a classifier from the strongest patterns and classify the cells with all other pattern strengths. Then, we analyze the accuracy depending on the pattern strength (Fig S32d, magenta). The True Negative rate for these curves is 100% (no random sample is detected as being non-random). This can be achieved, because the class dependent feature distributions are well separated for the strongest patterns.

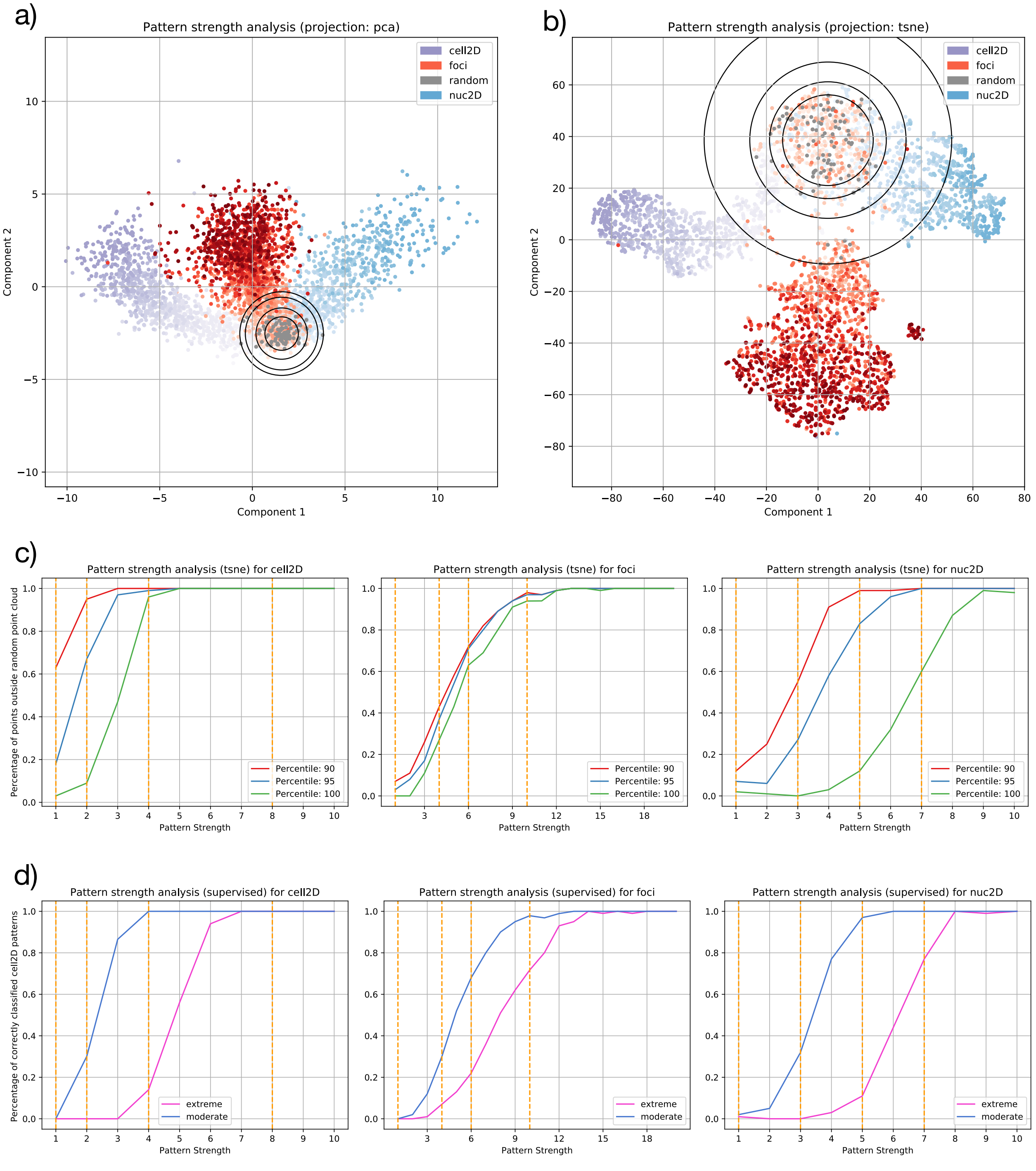

Supplementary Figure 32. Analysis of pattern strength. ***a)*** *PCA projection of simulated cells of random and 3 non-random localization classes. The color shade corresponds to pattern strength. For random localization, there is no pattern strength parameter defined. Circles correspond to 70, 90, 95 and 99 percentiles of random localization.* ***b)*** *t-SNE projection. Color code and circles are defined as in a).* ***c)*** *Pattern strength analysis: Percentage of cells with non-random localization pattern falling outside the normal distributions for the t-SNE projection. A cell is considered to be outside the random distribution, if its distance to the random centroid exceeds the 90, 95 or 100 percentile, as calculated from the random class. Orange lines indicate pattern strengths visualized in Fig S33.* ***d)*** *Percentage of correctly classified non-random patterns depending on pattern strength and depending on the training setting: for extreme, the strongest pattern and the random class has been used for training. For moderate patterns with strength larger than 3 have been used. Classes were balanced. Testing was performed on all samples not used for training. Orange lines indicate pattern strengths visualized in Fig S33.*

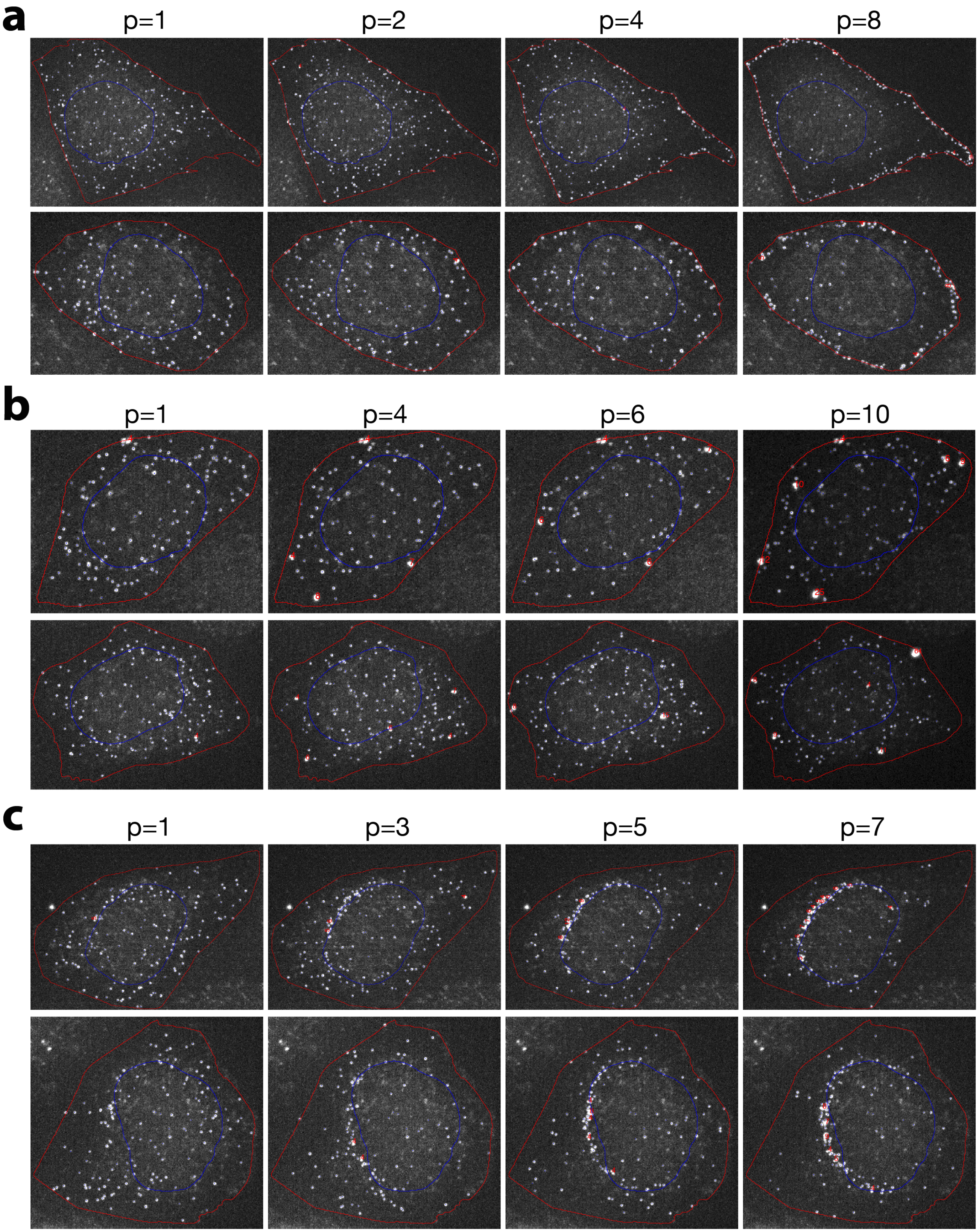

Supplementary Figure 33 *– Simulated cells with localization patterns of different strengths.* ***a)*** *Localization to the cellular membrane,* ***b)*** *Localization in mRNA foci,* ***c)*** *Localization at the nuclear envelope. Pattern strength as indicated at the top of each panel.*

From Fig S32d, we see that while we reach high accuracies for strong patterns, many weak and moderate patterns are classified as being random. Indeed, we did not provide the classifier enough examples to learn the distinction between more subtle forms of the pattern and random localization. This changes when we include weaker patterns in the training set. In Fig S32d, we show the pattern strength dependent performance, when the training set was drawn randomly from cells with pattern strength > 3 (we drew 100 samples to match the size of the random class; samples in the training set were not used for testing). As expected, the accuracy increases for weaker patterns, while the accuracy for random patterns remains high (99-100%). These results are not surprising: it is a good illustration of how to generate a training set: if the training set only contains clear cut examples, more subtle differences between patterns are not properly learned, and it is likely that the system underperforms when confronted to intermediate cases. A good training set therefore needs to represent the variability and subtlety of the test data the classifier is ultimately applied to. This includes more borderline cases.

Altogether, we have shown how to use the simulation framework for sensitivity analysis, providing us with an intuition on the differences between patterns that can still be distinguished. Such an application could prove particularly useful in the case of perturbation studies, where we want to analyze the effect of a drug or a gene silencing experiment, and we therefore need to understand how well subtle pattern differences can be distinguished.

4.8. SUMMARY OF PROPOSED METHOD

In this section, we describe in detail an analysis method that allows the identification of localization patterns from smFISH data sets in an unsupervised way. We carefully validated existing localization features and designed new features to allow the identification of localization patterns that were otherwise missed. We found that projecting the high-dimensional feature vectors by t-SNE considerably increases the classification accuracy. A challenge can remain the identification of the number of classes in a fully non-supervised fashion – especially with increased heterogeneity of the data. Here, methods such as the silhouette score or the within-class-variability can provide good indications. However, the most relevant number of classes has also to be verified by critical inspection of the data, e.g. by using visualization tools such as the t-SNE approach.

Supplementary Note 5: Analysis of mRNA localization in experimental data

To validate and further develop the analysis workflow presented in the preceding section, we analyzed experimental data from 10 different genes with manually annotated mRNA localization patterns (Table S3 and Fig S34) at the gene level (however without annotation at the cell level).

Gene	Observed localization pattern
MYO18A	Random
KIF20B	Random
PAK2	Random
RAB13	Cellular extension
KIF1C	Cellular extension
BUB1	mRNA foci
DYNC1H1	mRNA foci
SPEN	Intra- and perinuclear
ATP6AP2	Intranuclear and polarized against the nuclear envelope
CEP192	Intranuclear

Supplementary Table 3*. List of genes and manual annotated mRNA localization pattern (at the gene level). In the analysis, 150-400 cells per gene were considered, with a total of 2600 cells.*

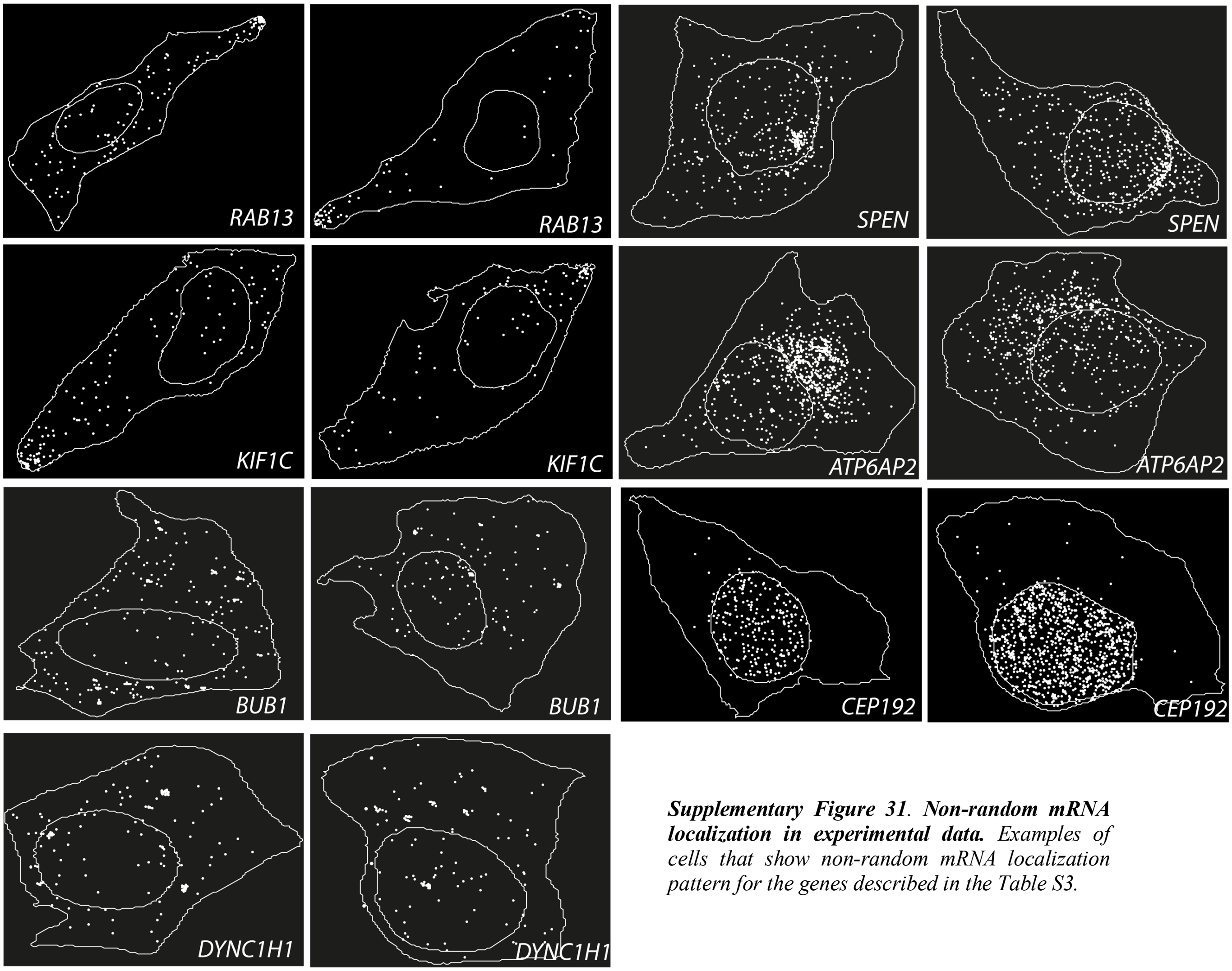

Supplementary Figure 31*.* ***Non-random mRNA localization in experimental data.*** *Examples of cells that show non-random mRNA localization pattern for the genes described in the Table S3.*

We performed automated segmentation of nuclei and cells with deep neural networks (see Online Methods for more details). mRNA detection and localization feature calculation were performed with the same Matlab functions used in the analysis of simulated data (see Online Methods for more details). For the subsequent analysis, we removed cells with fewer than 30 mRNAs. We further excluded mRNAs resulting from the GMM decomposition inside the nucleus, since these are most likely actively transcribing genes.

5.1. T-SNE VISUALIZATION OF EXPERIMENTAL DATA

First, we visualized the cellular feature vectors measured from the experimental data by t-SNE (Fig S35), where each point corresponds to one cell and the color to the RNA identity.

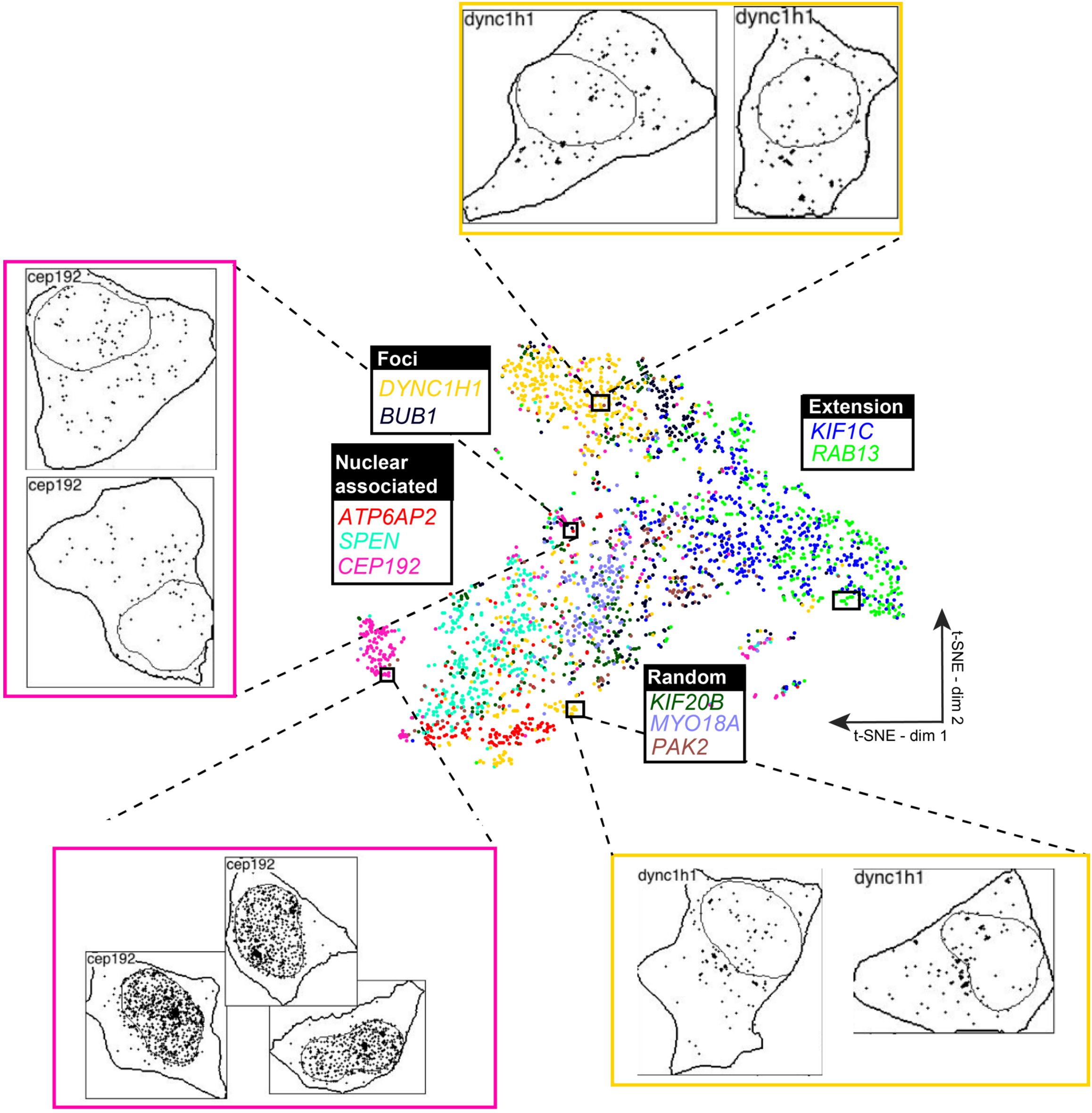

Supplementary Figure 35. *t-SNE projection of cells from experimental data. Each data-point is a cell colored according to the identity of the visualized mRNA.* ***(Black rectangles)*** *Parts of the deepzoom image generated from the t-SNE projection. The entire image can be found at* *https://muellerflorian.github.io/locFISH_deepzoom/#results/tsne_exp*

We observed that according to the RNA identity, the cells populated different regions in the feature space. Moreover, cells with different RNA identities grouped globally together in agreement with the manually annotated localization patterns at the gene level (Table S3):

- Random localizations are located in the central part (*KIF20B*, *MYO18A* and *PAK2*).
- Localization towards the cellular extensions are towards the right (*RAB13* and *KIF1C*),
- mRNA foci are at the top (*DYNC1H1* and *BUB1*),
- Nuclear associated localizations are located at the bottom (*CEP192*, *SPEN* and *ATP6AP2*).

We found that t-SNE plot is an excellent tool for an exploratory inspection of the data. We therefore provide detailed instruction for how to create large zoom-able images (deepzoom) of the t-SNE plots. In these images, each data-point is represented by a thumbnail showing (i) the outline of the cell and its nucleus, (ii) the positions of the detected mRNAs, (iii) the name of the gene (For examples, see Fig S35). This visualization provides an excellent tool to appreciate the complexity of these data as illustrated with a few examples. First, not all cells of the same targeted mRNA have preferential mRNA localization. For instance, most of the *CEP192* cells form a clearly separated cluster with a quasi-exclusive localization of mRNAs in the nucleus, while a few *CEP192* cells do not show this localization pattern and are grouped together with genes showing a random localization pattern. Second, some mRNA localization patterns – as we will explore later - are the mixture of pure patterns. Transcripts of *DYNC1H1* form mRNA foci, which are in general displayed in the upper part of the t-SNE plot. However, in some cells these foci are located towards the nuclear envelope, and these cells are in close proximity with other cells displaying nuclear localization.

Taken together, this exploratory analysis shows that our analysis workflow is capable of correctly extracting quantitative information about mRNA localization from experimental data. It also reveals interesting heterogeneity features that can occur on two different levels: (1) on the population level where for a given gene some cells show a pattern, while others do not; (2) the single-cell level, where a cell can show a mixture of different localization patterns.

5.2. ANALYSIS OF A DIFFERENT CELL TYPE

We next tested the analysis workflow on a different cell line C2C12 (immortalized mouse myoblast cell line), while HeLa cells are human epithelial cells derived from an adenocarcinoma. Thus, these two lines are different in terms of morphology and size (Fig S36).

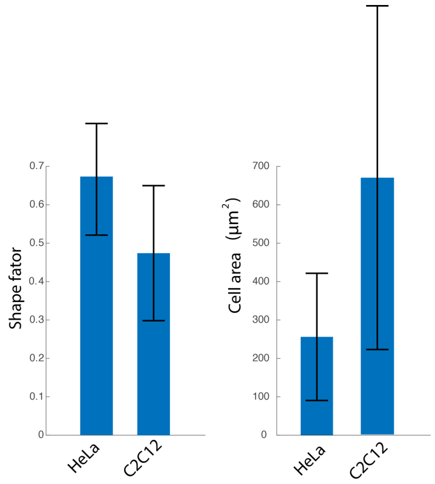

Supplementary Figure 36. *Comparison of size and shape of HeLa and C2C12 cells. Parameters were estimated from 2D segmentation masks. The shape factors gives an indication about the objects shape, it is calculated as 4*pi*area/(perimeter^2). A perfect circle has a value of 1, while a thin thread-like object has the lowest shape factor approaching 0. Error bars indicate two standard deviations.*

We performed smFISH against 3 genes with different annotated localization patterns: *DYNC1H1* (foci), *KIF1C* (cellular extension), *ACTN1* (random). We analyzed the data with the exact some analysis workflow as used for HeLa cells. t-SNE analysis (Fig S37) shows that depending on the localization patterns, the cells are projected to different regions in the t-SNE space. We intended ACTN as a random, as we had manually checked that localization was random. After our quantitative analysis, we observed that there was indeed a large subpopulation with random localization (Fig S37), but there was also another subpopulation showing polarized localization, which has then been validated by manual inspection (Fig S37). These results demonstrate that the established workflow is well suited to distinguish localization patterns in other cell lines than HeLa.

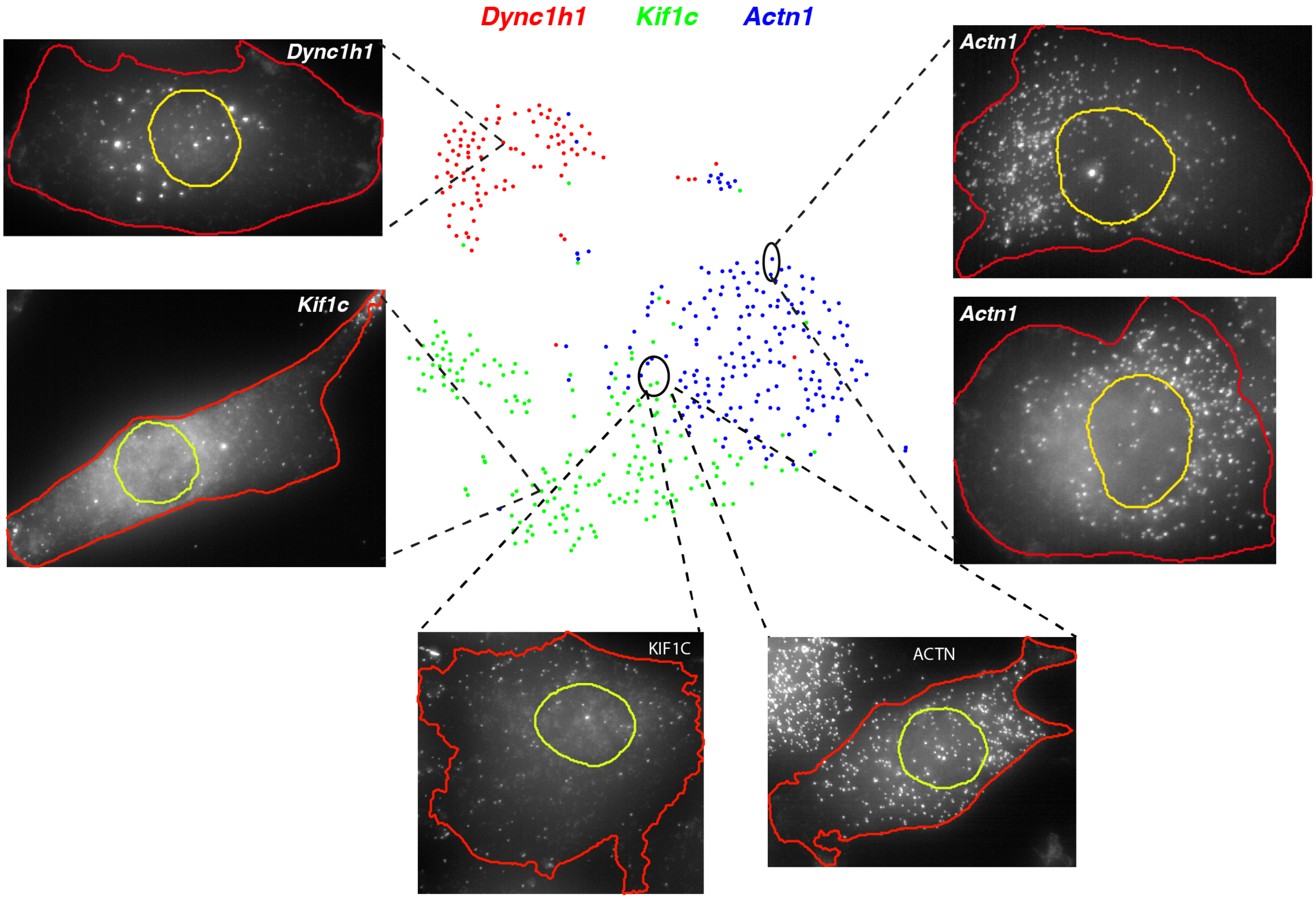

Supplementary Figure 37. *t-SNE projection of experimental data for C2C12 cells. Each data-point is a cell colored according to the identity of the visualized mRNA.*

5.3. UNSUPERVISED CLUSTERING OF EXPERIMENTAL DATA

We next performed unsupervised clustering, to see if these approaches can identify the annotated localization classes despite the above-mentioned heterogeneity. Before performing k-means clustering, we pre-processed the feature space with a 6-dimensional t-SNE projection. In order to determine the best number of classes, we computed the silhouette score and the within-cluster-variability. The silhouette score gave almost similar values for 3 and 4 classes (Fig S38a), while the within-classes-variability still shows an important decrease between 3 and 4 classes (Fig S38a). We therefore chose 4 classes in a k-

means clustering, which correctly grouped the genes with similar annotated localization pattern (Fig S38b-c).

These clustering results also show some limitations of k-means, in particular with respect to strongly unbalanced and/or non-spherically distributed data. An example in our data are *CEP192* cells with a strong intranuclear localization. Despite being well separated from all other cells in the t-SNE projection, the *CEP192* cells are not identified as a separate cluster by k-means. We therefore performed spectral clustering, an unsupervised clustering approach, which is related to k-means but less sensitive to cluster size. With this approach, we could clearly detect the cluster of *CEP192* cells, but at the cost of grouping together random cells and the other nuclear localization (Fig S38d). Increasing the number of clusters did not solve this issue. Indeed, while different regions seem to correspond to different localization patterns, the clusters are not necessarily clearly separated, which is a pre-requirement for many clustering algorithms.

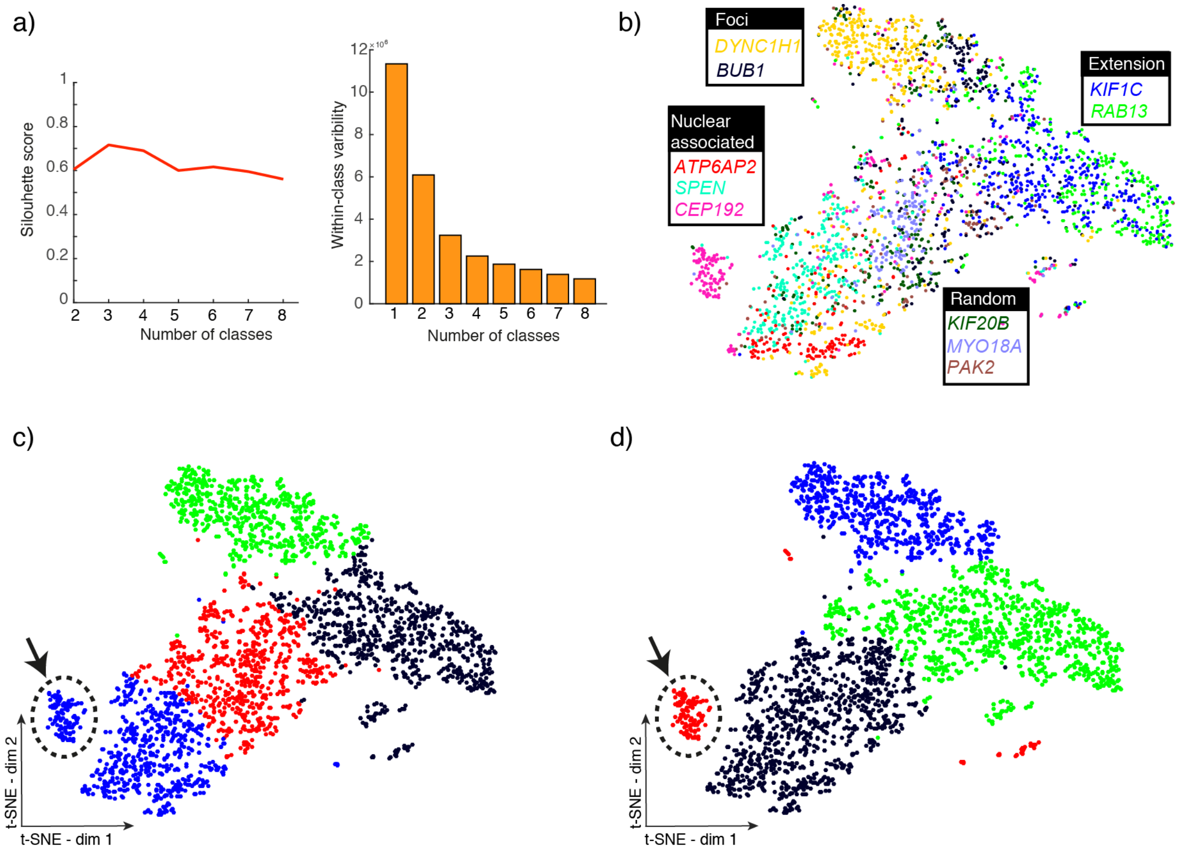

Supplementary Figure 38. Unsupervised clustering of experimental data. a) *Plot of the within-cluster-variability and silhouette score for clustering with different numbers of classes.* ***b)*** *t-SNE projection experimental data. Each point is a cell colored according to the gene. Please note that the t-SNE projection is not deterministic which explains the difference to Fig S35a.* ***c)*** *Same as b) but data-points are colored according to the clustering results. Please note that shown is a 2D t-SNE projection, while the clustering was performed on a 6D t-SNE projection.* ***d)*** *Results of spectral clustering of data in b).*

These results illustrate that unsupervised methods can be used to analyze experimental data, but their limitations have to be considered, i.e. we found that k-means clustering correctly identifies the main localization classes, while spectral clustering is better suited to find classes with strong localization signatures, clearly separated from other classes.

To further analyze the k-means classification, we determined for each gene the distribution of cells in the 4 identified classes (Fig S39a). This shows that genes with the same manual annotation, have largely similar profiles. This heatmap also revealed genes with a combination of localization classes. One example is *DYNC1H1,* which we annotated to have mRNA foci. However, a small proportion of *DYNC1H1* cells where found in class 4 (nuclear localization). Inspection of these cells revealed that these cells display mRNA foci in proximity of the nuclear envelope (Fig S39b). This analysis confirms the localization heterogeneity observed in the t-SNE plots. However, it is important to point out that k-means performs a hard-assignment, i.e. one cell is attributed to one localization class and is not described by a mixture of patterns. We explore the possibilities of a true multi-label assignment in a next section on supervised learning, which will allow us to explore localization heterogeneity in more detail.

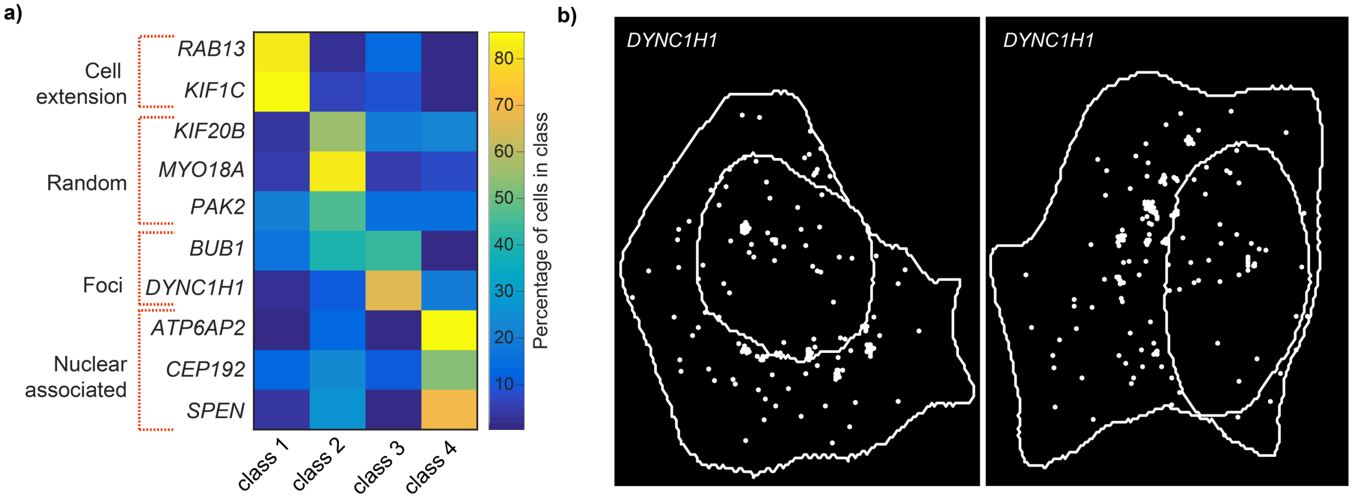

Supplementary Figure 39. Unsupervised clustering of experimental data. a) *Heatmap of the distribution of cells of each gene in the 4 classes displayed in Fig S38c.* ***b)*** *Examples of cells with DYNC1H1 showing mRNA foci close to the nuclear envelope.*

5.4. HIERARCHICAL CLUSTERING OF EXPERIMENTAL DATA

In order to further inspect the variability and complexity of the data, we turned to hierarchical clustering (Matlab function *clustergram*, linkage method *ward*). This approach has two interesting differences compared to k-means: (1) the number of classes is not specified, (2) the visualization as a dendrogram provides a direct visual feedback of the similarity of the clusters and which features are shared for cells that are grouped together. The dendrogram below (Fig S40) shows the analysis results on experimental data. When considering a classification with 4 classes as for k-means, we find a clustering that groups mostly genes belonging to one of the 4 large localization classes (random, foci, cellular extensions, nuclear localization).

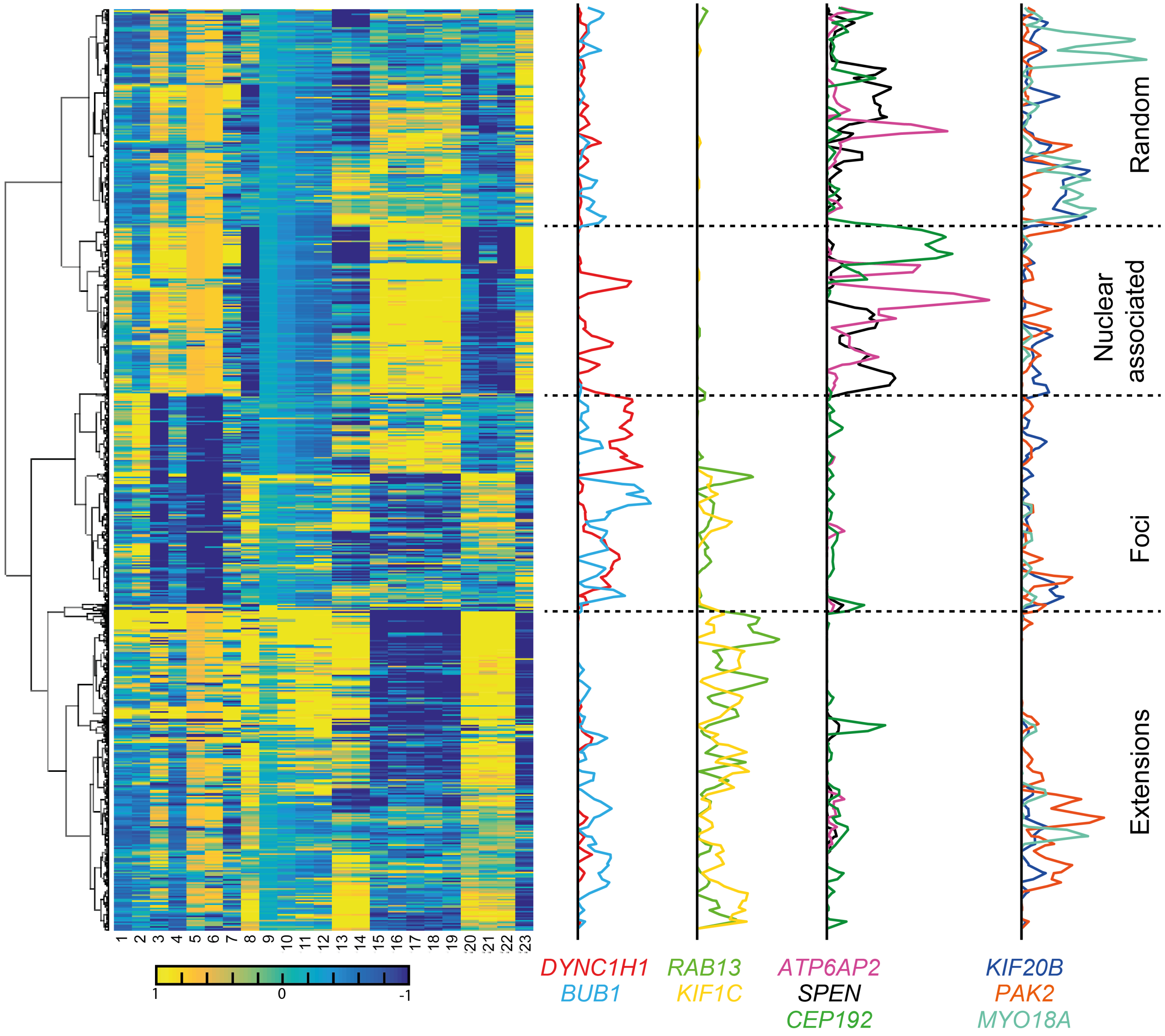

Supplementary Figure 40. Hierarchical clustering of experimental data. *Each cell corresponds to a line, each feature to a column (See list of features with corresponding index in Table S4). The dashed lines indicating the classification with four classes. The line profiles shown on the right are the smoothed enrichment distribution of each gene along the classification results.*

**1**	Ripley: maximum	**13**	Cell height: spearman correlation with Z_{mRNA}
**2**	Ripley: max gradient [0,max]	**14**	Cell height: R^2 with Z_{mRNA}
**3**	Ripley: min gradient [max,end]	**15**	Cell membrane: distance – mean
**4**	Ripley: value at mid-point between center and boundary	**16-19**	Distance membrane: quantile 5%, 10%, 20%, 50%
**5**	Ripley: Spearman correlation between Ripley and radius	**20**	Nucleus: distance – mean
**6**	Ripley: radius of max value	**21**	Cell centroid: distance – mean
**7**	Polarization index	**22**	Nucleus centroid: distance – mean
**8**	Dispersion index	**23**	Ratio: mRNAs inside nucleus/outside nucleus
**9 -12**	Morph opening - enrichment ratio: 15, 30, 45, 60 pixels		

Supplementary Table 4*. List of localization features used in the dendogram in Fig S40.*

However, we can also appreciate variability within each of these four large clusters, where sub-clusters with clear signatures can be seen. For instance, *CEP192* cells form a tight cluster with the typical features for a nuclear localization being present, e.g. small distance to the nuclear centroid (feature 22) or a high percentage of the mRNA in the nucleus (feature 23). The direct interpretability of many of the localization features allows directly inferring the potential origin of differences in the sub-cluster. For instance (Fig S41), the cells in the group labeled 'foci' all carry a typical signature of mRNA foci (mainly features 3, 5, and 6). However, within this group there are some cells that carry the signature of a localization towards the cell membrane in 2D (close to the cell membrane, far from the nucleus; features 13-23), while others carry the signature of a localization towards the nucleus. The latter is strongly enriched with *DYNC1H1*, which we described already before a gene having foci, and occasionally displaying them close the nuclear envelope. This illustrates how localization heterogeneity can occur at the single cell level and is captured by the different localization features.

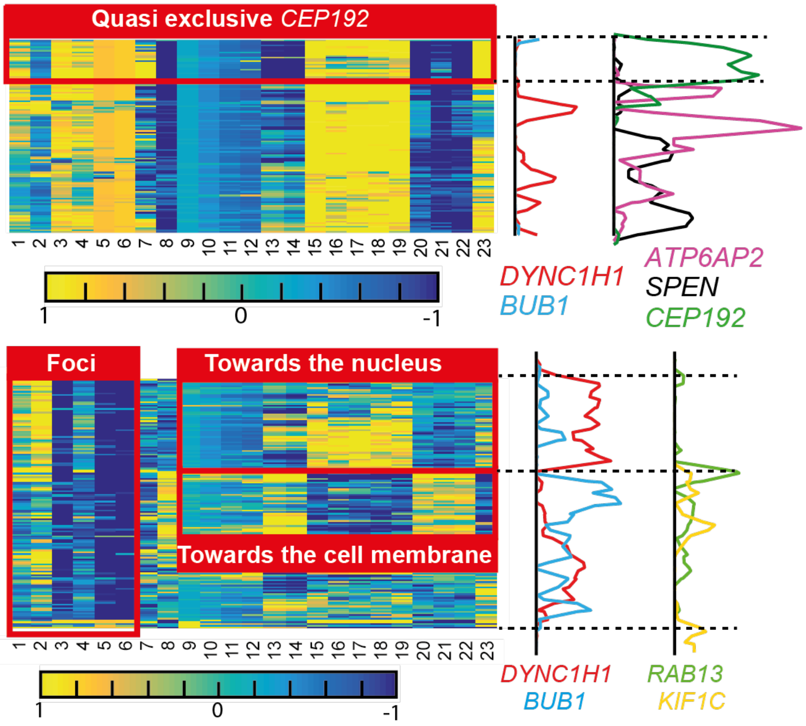

Supplementary Figure 41. Hierarchical clustering of experimental data.

Upper panel *shows cluster corresponding to nuclear associated mRNAs localization. Some CEP192 cells form a sub-cluster carrying the signature of strong intra-nuclear localization.*

Lower panel *shows only the cluster corresponding to mRNA foci. Each red rectangle highlights a group of features that carries a signature of the indicated localization pattern.*

5.5. SUPERVISED CLASSIFICATION OF EXPERIMENTAL DATA

The analysis of experimental data with different unsupervised clustering approaches correctly identified the different localization classes but also revealed complexity in the data that could remain unaccounted for when using these approaches. First, certain localization patterns can be observed only in a few cells compared to the overall cell count (e.g. the *CEP192* cells with strong intranuclear localization). In a large-scale screen this could for instance correspond to rare events related to certain cell cycle phases. Second, all clustering algorithms make some explicit or implicit assumptions on the feature distributions in order to define the clusters. In practice, there is of course no guarantee that these assumptions hold. It is nearly always beneficial to make us of prior knowledge on the biological meaningful patterns to detect. Third, cells can have a mixture of localization patterns, i.e. they can have a predominant localization pattern (such as foci) but with a different localization preference (either towards the nucleus or the cell membrane). These mixed patterns may suggest multiple functions of the encoded proteins or snapshots of a dynamical process of translocation and are therefore interesting to analyze in detail. In this section, we

show how supervised learning can be used to explore these important aspects. In particular, we show that we can learn from simulated data and apply the learned classifier to experimental data. This is a particularly elegant way of addressing the problem of rare localization patterns in a screening application and patterns that are difficult to interpret manually (such as membrane localization).

Evaluating similarity between simulated and experimental data

Supervised classification approaches require training on annotated data. In our case, such annotated training data is not available from experiments and we therefore turned to our simulated data. In order to evaluate the similarity between simulations and experiments, we pooled both data sets and performed a t-SNE analysis. Specifically, we choose the following 5 simulated patterns, which – among the defined localization patterns - describe best the localization patterns observed in the experimental data:

- Random
- Cellular extension
- Foci
- Nuclear envelope 3D
- Nuclear envelope 2D

It must be noted, that the two simulated nuclear patterns (nuclear envelope 2D and 3D) do not correspond exactly to the patterns we observe in the experimental data. This is actually a realistic scenario, when using simulations for supervised learning: it is indeed very likely that some of the classes in real data only approximately correspond to some simulated class, and that conversely, we do not think of all possible classes that can occur when we design the simulations. We have therefore the realistic situation where one pattern (*CEP192*; intra-nuclear) has not been accounted for in the simulations but is reasonably close to one of the simulated classes (nuclear envelope 3D).

Figures S42 shows that the different localization patterns from simulations and experiments populate close regions in the t-SNE plot: overall, we observe thus good agreement between simulations and experiments. However, we also note that the distributions do not coincide. For some patterns the differences are marginal, for other patterns they are more important.

With these considerations and preliminary analyses, we next investigated whether – despite of the differences between simulation and experimental data – we can learn a classifier from the simulated data and apply it to experimental data.

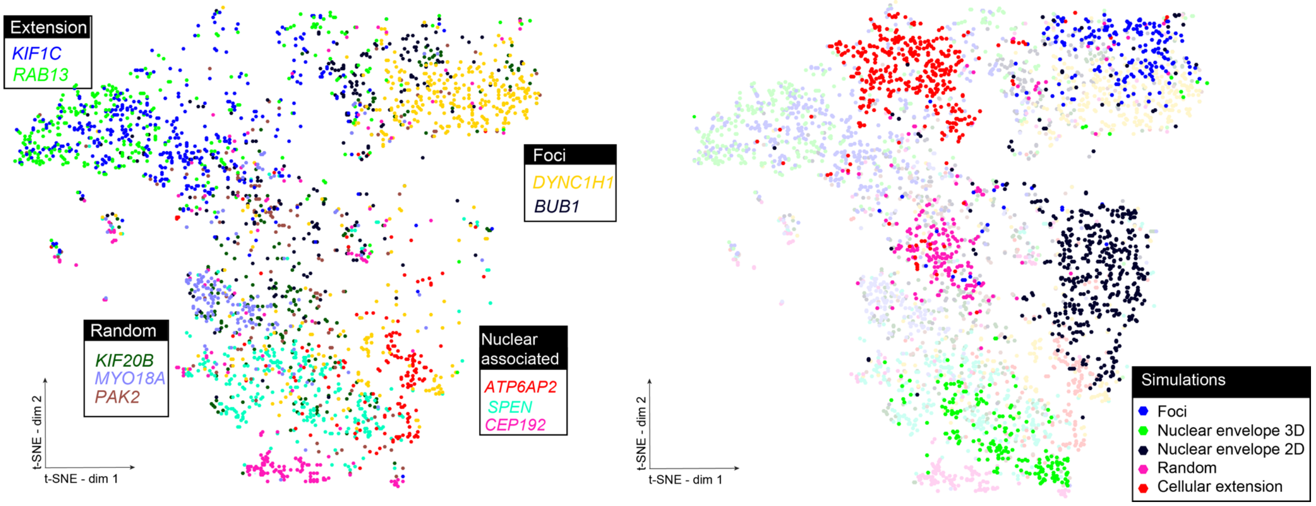

Supplementary Figure 42. Comparison of experimental and simulated data. a) t-SNE projection performed with experimental data and simulations with different localization patterns (mRNA foci, nuclear localization, cellular extension and random). Shown are only the experimental data. b) Same as a), except that experimental data is shown transparent and simulated data is now also shown.

Supervised classification of experimental data

We next explored if it was possible to learn a classifier from simulated data, and then apply it to experimental data. We therefore trained a Random Forest Classifier (RF) with 100 trees (using the MATLAB function *treebagger*) on 5 different localization classes (mRNA foci, nuclear envelope 3D, nuclear envelope 2D, cellular extensions and random). We trained the RF on the full set of localization features, and not on the pre-processed set with a t-SNE projection. This is necessary since the t-SNE projection – in contrast to PCA - is not deterministic, and a classifier trained on t-SNE pre-processed feature space could not be applied to new data. We then applied the trained random forest to experimental data (Fig S43a-b). Interestingly, the assigned localization classes correspond to the manual annotation classes and is comparable to the k-means clustering (compare to Fig S43c). The biggest difference occurs for the gene with a nuclear localization, which are now – per definition - split into two classes, one corresponding to an enrichment inside the nucleus, and one outside.

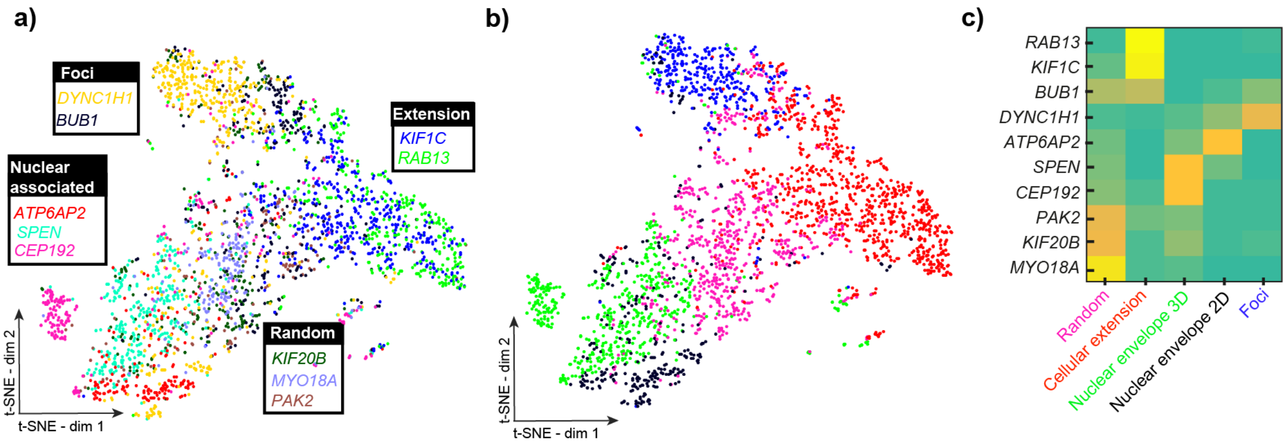

Supplementary Figure 43. Supervised classification of experimental data. a) t-SNE projection of experimental data. Each point is a cell colored according to its gene. b) Clustering results obtained on experimental data. Same t-SNE plot as in a) but data-points are colored according to the clustering results. Color-code for localization classes indicated in c) Please note that clustering was performed on the raw data. c) Heat-map of the posterior probability for each gene to belong to the 5 trained localization classes (calculated as the mean of all cells for a given gene).

Comparison to manually annotated data

We next wanted to investigate whether a classifier trained on simulated data f_{sim} can achieve similar accuracy as a classifier trained on manually annotated data f_{manual}. For this, we manually annotated 500 cells according to their localization pattern (the analysis is necessarily restricted to those patterns for which we actually have real data). Even though we know that there can be errors and inconsistencies in such a manually annotated data set, we hypothesized that this might affect both classifiers f_{sim} and f_{manual}.

It is important to understand that f_{manual} learns a rule from a data set that follows the same distribution of localization features as the benchmark data set, while f_{sim} learns a rule from a data set drawn from a different – albeit similar - distribution. We can therefore not expect f_{sim} to outperform f_{manual}, but we can get an indication on how much the distribution divergence impacts classification results.

All results were obtained by classification of samples that were not used for training. For f_{sim}, the training set consisted in simulated images and for f_{manual}, all reported results are calculated from out-of-bag

classifications. In order to compare the two classifiers, we display the confusion matrices and the overall accuracy (Fig S44a). For each class, we also show precision, recall, specificity, and F1-score (Fig S44b). Finally, we also show the average values of these metrics in Fig S44c.

The results presented in Fig S44 suggest that the two classifiers perform equally well on the manually annotated data set in terms of overall accuracy and all average and class-specific metrics we have calculated. The error distribution is not identical, but the differences are marginal.

We can therefore conclude that a classifier trained on simulated data performs equally well on manually annotated data as a classifier that was trained on manually annotated data.

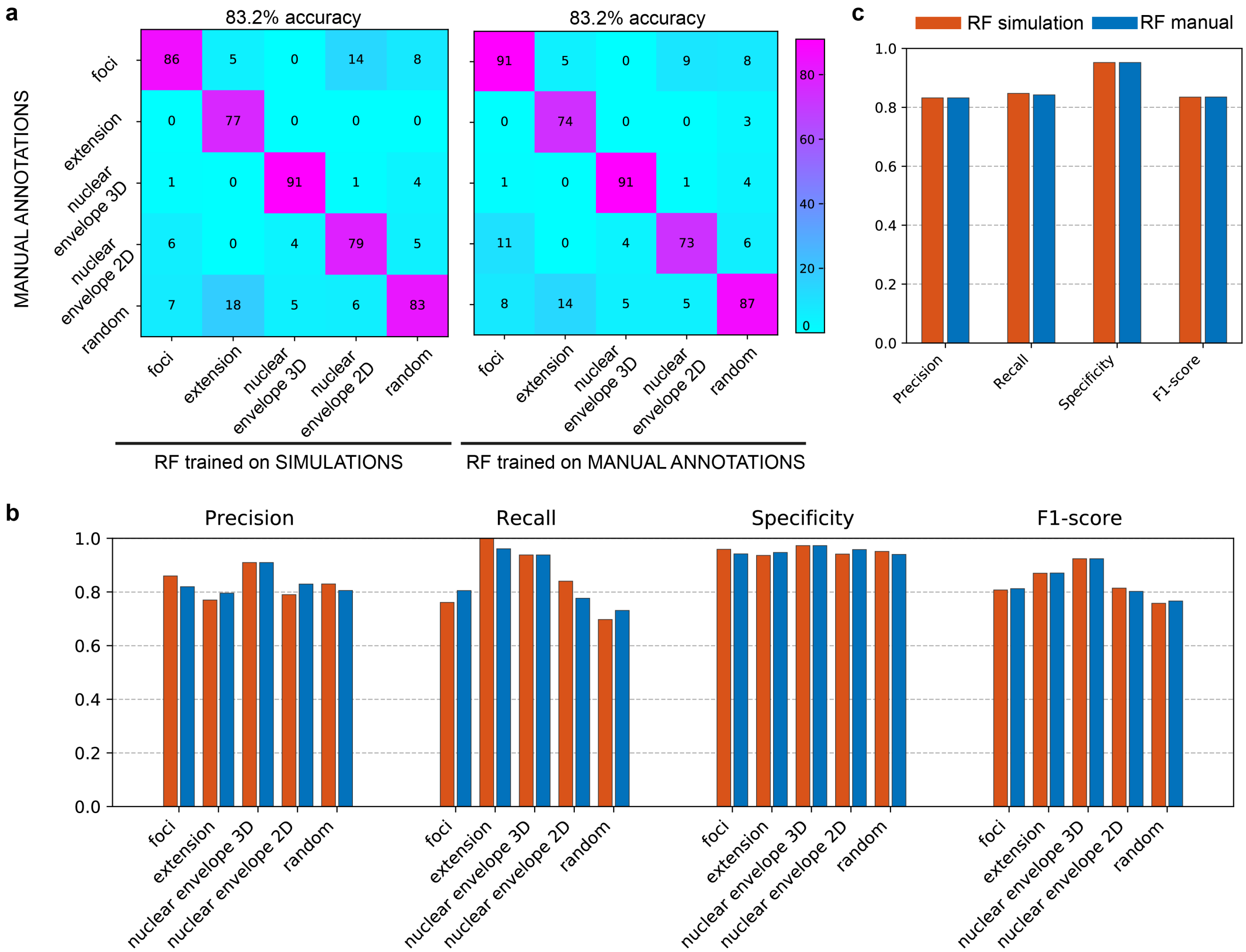

__Supplementary Figure 44.__ Performance comparison of random forest classifiers trained on manually annotated and simulated data. __a)__ Confusion matrices for both classifiers f_{sim} (left) and f_{manual} (right). The overall accuracy is displayed on top of the confusion matrices. __b)__ Precision, recall, specificity and F1-score for individual classes. __c)__ Precision, recall, specificity and F1-score, averaged over all classes.

Description of cells with multiple localization patterns

As before, we can see that for multiple genes different localization classes are enriched at the population level (Fig S43c). However, a random-forest classification provides us with the possibility to further analyze this heterogeneity.

From the Random Forest Classifier, we obtain the posterior probabilities for each cell and each class, i.e. we can assign to each cell a vector of probabilities of belonging to each of the classes given the features describing the spatial distribution of transcripts. A traditional classification approach assigns one class to each cell, by choosing the class of maximal posterior probability (Fig S43c). Alternatively, the entire vector can be used to describe a given cell with a weighted combination of different localizations patterns (Fig S45). These plots reveal that each gene shows a strong enrichment for the class according to its global manual annotation. We also observe varying degrees of heterogeneity, either because different cells correspond to different profiles or because inside each cell, posterior probabilities for different classes are relatively large.

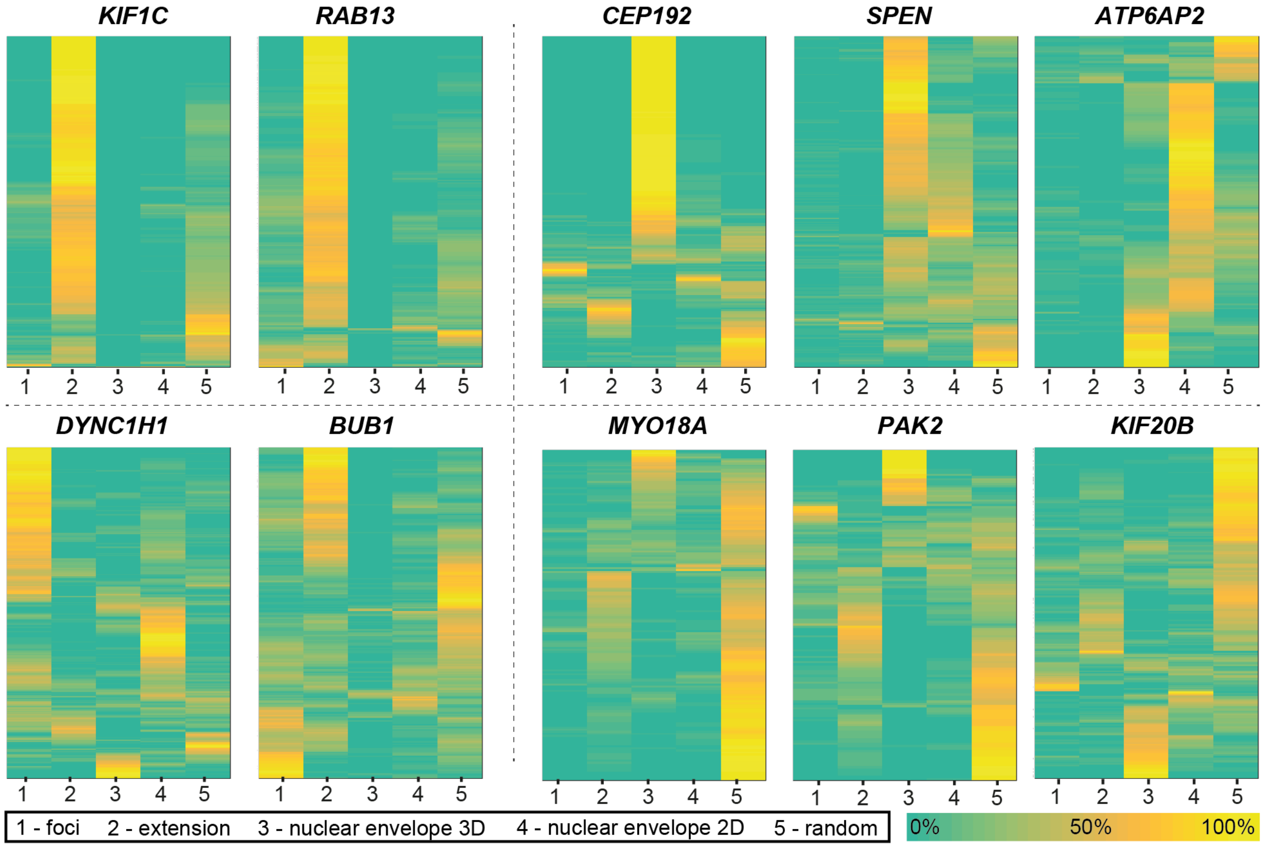

***Supplementary Figure 45. Supervised classification of experimental data**. Plot shows posteriori probabilities for each gene. Rows are individual cells, columns are localization classes. Order of columns is identical for each gene, as indicated in lower part of the figure. Dashed lines separate genes into the different annotated classes.*

- *KIF1C* and *RAB13* are both strongly enriched in the “cellular extension” class. However, *RAB13* shows some cells with foci, while *KIF1C* shows more mixture with random. This corresponds to a manual inspection, where *RAB13* shows a stronger enrichment in the extension than *KIF1C*, leading to foci at the extensions.
- For the nuclear localizations, most *CEP192* cells have been labeled as nuclear envelope 3D pattern. Most of the remaining cells have been assigned to the random pattern, and in small proportions to the other classes. *ATP6AP2* on the contrary is mostly nuclear envelope 2D, while *SPEN* is frequently a mixture of both, at the single cell level. This also corresponds to manual observations, where *SPEN* shows often cells with mRNA in the nucleus or in its close proximity, while *ATP6AP2* shows a distribution of mRNAs, which extends further away from the nucleus.

Heterogeneity in mRNA localization

This analysis revealed that mRNA localization heterogeneity can occur at two different levels

(1) intracellular: within a given cell, e.g. a cell shows a mix of patterns.

(2) population: between different cells in the cell population, e.g. different cells have different patterns

In order to better describe the origin of the observed heterogeneity, we calculate the so-called Gini impurity from the vector of posterior probabilities:

$$I_{\mathrm{G}}(p) = \sum_{i=1}^{N_{\mathrm{class}}} p_i(1 - p_i),$$

where the p_i is the posterior probability of belonging to localization class i. In the extreme cases, the Gini impurity has a minimum value of 0 if there is one class with posterior probability 1 and the other classes have probability 0, and a maximum value of $\frac{N_{\mathrm{class}}-1}{N_{\mathrm{class}}}$ if all classes have the same probability.

To judge population heterogeneity, we calculate I_G on the mean posterior probability for each gene

$$I_{\mathrm{G}}(population) = \sum_{l=1}^{N_{\mathrm{class}}} \overline{p_l}(1 - \overline{p_l}),$$

where, $\overline{p_l} = \frac{1}{N_{\mathrm{cell}}} \sum_{j=1}^{N_{\mathrm{cell}}} p_{i,j}$, $p_{i,j}$ is the probability of the cell j to belong to the localization class i, N_{class} is the number of localization classes, N_{cell} is the number of cells.

To judge intracellular heterogeneity, we calculate I_G for each cell of a gene, and then determine the mean value

$$I_{\mathrm{G}}(intra) = \frac{1}{N_{\mathrm{cell}}} \sum_{j=1}^{N_{\mathrm{cell}}} I_{\mathrm{g}}(cell, j),$$

where $I_{\mathrm{G}}(cell, j) = \frac{1}{N_{\mathrm{class}}} \sum_{i=1}^{N_{\mathrm{class}}} p_{i,j}(1 - p_{i,j})$.

We then plot the two values against each other in a scatter plot (Fig S46). In order to better understand this plot, take the example of *CEP192* versus *MYO18*. Both genes have a similar population impurity. However, *CEP192* has the lower intracellular impurity, this means that individual cells are more homogenous, i.e. they tend to show only one population pattern. Indeed, *CEP192* has predominately cells that are either nuclear envelope 3D or random. This means that the observed heterogeneity for *CEP192* is caused by different cell populations each showing pure, yet different patterns. In the case of *MYO18A*, the heterogeneity results from pattern mixtures in individual cells. Another illustrative example is *KIF1C* and *CEP192*, where the main difference is on the population level. This means that at the level of individual cells both genes are relatively homogeneous. The difference in the population score means that *CEP192* has different patterns in different cells, while for *KIF1C* one pattern is pre-dominant.

Supplementary Figure 46. Heterogeneity of mRNA localization. *Plot shows Gini impurity calculated either for the posteriori probabilities of the random-forest classification.*

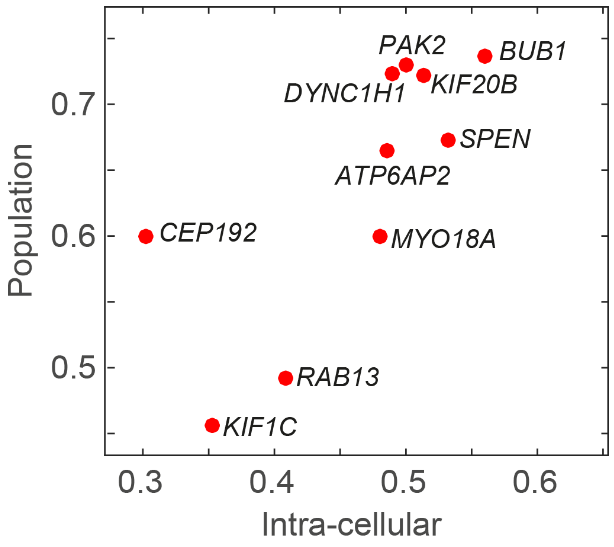

Population and intracellular heterogeneity have different origins and have to be interpreted accordingly. The existence of different localization patterns in different cells for a given gene could be indicative of different biological states. Here the localization pattern could be the approximation of these cellular states. Interpretation of pattern mixtures is more complicated. If the patterns are mutually exclusive (for instance, if a fraction of mRNA localizes at the nuclear envelope and another subset of mRNA at the cell membrane), this suggests diverse co-existing localization patterns and may point to a double function of the encoded protein. If the patterns are not mutually exclusive (for instance organization into foci, where foci can be at different locations in the cell), this rather indicates existence of subclasses, i.e. subtleties inside the defined classes, that have not been defined as separate classes. These subtleties could potentially bare important information, as they might be indicative of different biological functions, e.g. by interaction with different cellular components.

5.6. ANALYSIS OF MRNA LOCALIZATION IN DIFFERENT EXPERIMENTAL CONDITIONS

The presented workflow can also be used to study the impact of perturbation on mRNA localization. In a recent study, we reported that *DYNC1H1* mRNAs aggregate to form foci. We further reported that these foci do not overlap with P-bodies or stress granules, but they are translational factories[22]. To test the role of translation of the formation of foci, we treated cells with Puromycin, a terminator of translation elongation. Visual examination indicates that the foci disappear after a brief treatment with Puromycin (Fig S47a).

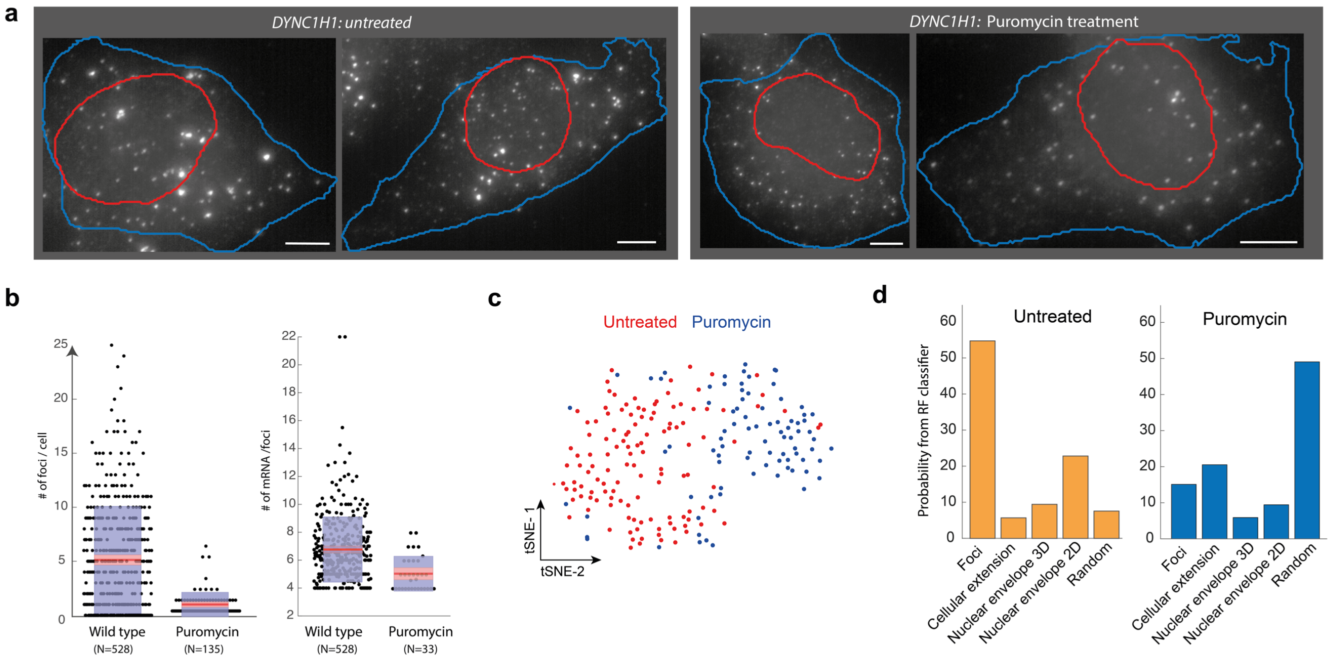

***Supplementary Figure 47**. **(a)** Representative images of DYNC1H1: untreated (left) and after Puromycin treatment (0.1 mg/mL diluted in the culture medium for 30 minutes, right). Shown are maximum intensity projections, and the segmented cell and nuclei. Same intensity scaling was applied to all images to allow direct comparison. Scale bars 5 µm. **(b)** Comparison of foci quantification in DYNC1H1 WT and after Puromycin treatment. Bar plots show average number of foci per cell, and average number of mRNAs per foci. **(c)** t-SNE analysis of DYNC1H1 smFISH data. Each dot is one cell. Color-code according to molecular identify: untreated (red) and after treatment with Puromycin (blue). **(d)** Supervised classification of DYNC1H1 smFISH data. Left histogram shows probability distribution among different localization patterns for untreated cells, right histogram for cells after puromycin treatment.*

Such a change can be quantified directly, by comparing the most impacted localization features. For these data, we used the results of our GMM analysis and calculated the average number of foci per cell, and how many mRNAs are per foci (Fig S47b). We can also analyze these data with the unsupervised and supervised analysis methods used above. Fig S47c shows a t-SNE plot, where untreated and Puromycin treated cells occupy different regions. As an alternative, we analyzed these data with the supervised classification approach in our manuscript (Fig S47d). In agreement with the analysis above, we observe a loss of localization in foci after Puromycin treatment. In summary, this illustrates how the established framework can be used to study perturbation experiments, and it demonstrate that the formation of *DYNC1H1* foci is translation-dependent. These data challenge an established paradigm, which states that RNA localization is translation-independent.

Supplementary References

1. Naik, A. W., Kangas, J. D., Sullivan, D. P. & Murphy, R. F. Active machine learning-driven experimentation to determine compound effects on protein patterns. *eLife* **5,** e10047 (2016).
2. Coelho, L. P., Shariff, A. & Murphy, R. F. NUCLEAR SEGMENTATION IN MICROSCOPE CELL IMAGES: A HAND-SEGMENTED DATASET AND COMPARISON OF ALGORITHMS. *Proc. IEEE Int. Symp. Biomed. Imaging* **5193098,** 518–521 (2009).
3. Chen, K. H., Boettiger, A. N., Moffitt, J. R., Wang, S. & Zhuang, X. Spatially resolved, highly multiplexed RNA profiling in single cells. *Science* aaa6090 (2015). doi:10.1126/science.aaa6090
4. Shah, S., Lubeck, E., Zhou, W. & Cai, L. In Situ Transcription Profiling of Single Cells Reveals Spatial Organization of Cells in the Mouse Hippocampus. *Neuron* **92,** 342–357 (2016).
5. Battich, N., Stoeger, T. & Pelkmans, L. Image-based transcriptomics in thousands of single human cells at single-molecule resolution. *Nat. Methods* **10,** 1127–1133 (2013).
6. Zhao, T. & Murphy, R. F. Automated learning of generative models for subcellular location: building blocks for systems biology. *Cytom. Part J. Int. Soc. Anal. Cytol.* **71,** 978–990 (2007).
7. Svoboda, D. & Ulman, V. MitoGen: A Framework for Generating 3D Synthetic Time-Lapse Sequences of Cell Populations in Fluorescence Microscopy. *IEEE Trans. Med. Imaging* **36,** 310–321 (2017).
8. Padovan-Merhar, O. *et al.* Single mammalian cells compensate for differences in cellular volume and DNA copy number through independent global transcriptional mechanisms. *Mol. Cell* **58,** 339–352 (2015).

9. Tsanov, N. *et al.* smiFISH and FISH-quant - a flexible single RNA detection approach with super-resolution capability. *Nucleic Acids Res.* **44,** e165 (2016).

10. Held, M. *et al.* CellCognition: time-resolved phenotype annotation in high-throughput live cell imaging. *Nat. Methods* **7,** 747–754 (2010).

11. Mueller, F. *et al.* FISH-quant: automatic counting of transcripts in 3D FISH images. *Nat. Methods* **10,** 277–278 (2013).

12. BIG • PSF Generator. Available at: http://bigwww.epfl.ch/algorithms/psfgenerator/. (Accessed: 23rd July 2015)

13. de Moraes Marim, M., Bo Zhang, Olivo-Marin, J.-C. & Zimmer, C. Improving single particle localization with an empirically calibrated Gaussian kernel. in *Biomedical Imaging: From Nano to Macro, 2008. ISBI 2008. 5th IEEE International Symposium on* 1003–1006 (2008). doi:10.1109/ISBI.2008.4541168

14. Battich, N., Stoeger, T. & Pelkmans, L. Control of Transcript Variability in Single Mammalian Cells. *Cell* **163,** 1596–1610 (2015).

15. Cabili, M. N. *et al.* Localization and abundance analysis of human lncRNAs at single-cell and single-molecule resolution. *Genome Biol.* **16,** 20 (2015).

16. Wu, A. C.-Y. & Rifkin, S. A. Aro: a machine learning approach to identifying single molecules and estimating classification error in fluorescence microscopy images. *BMC Bioinformatics* **16,** 102 (2015).

17. Tinevez, J.-Y. *et al.* TrackMate: An open and extensible platform for single-particle tracking. *Methods San Diego Calif* **115,** 80–90 (2017).

18. Thomann, D., Rines, D. R., Sorger, P. K. & Danuser, G. Automatic fluorescent tag detection in 3D with super-resolution: application to the analysis of chromosome movement. *J. Microsc.* **208,** 49–64 (2002).

19. Jungmann, R. *et al.* Quantitative super-resolution imaging with qPAINT. *Nat. Methods* **13,** 439–442 (2016).

20. Maaten, L. van der & Hinton, G. Visualizing Data using t-SNE. *J. Mach. Learn. Res.* **9,** 2579–2605 (2008).

21. Park, H. Y., Trcek, T., Wells, A. L., Chao, J. A. & Singer, R. H. An Unbiased Analysis Method to Quantify mRNA Localization Reveals Its Correlation with Cell Motility. *Cell Rep.* **1,** 179–184 (2012).

22. Pichon, X. *et al.* Visualization of single endogenous polysomes reveals the dynamics of translation in live human cells. *J. Cell Biol.* **214,** 769–781 (2016).
