# Supplementary material for: A computational framework to study sub-cellular RNA localization
Source: Nat Commun. 2018 Nov 2;9:4584. doi: 10.1038/s41467-018-06868-w (PMC6214940; doi:10.1038/s41467-018-06868-w)
Supplement: Supplementary file 3 — Description of Additional Supplementary Files [file 41467_2018_6868_MOESM3_ESM.pdf]

Description of Additional Supplementary Files

File Name: Supplementary Data 1
Description: Sequences of oligonucleotides for all performed smFISH experiments.
